# The CAZyome of *Phytophthora *spp.: A comprehensive analysis of the gene complement coding for carbohydrate-active enzymes in species of the genus *Phytophthora*

**DOI:** 10.1186/1471-2164-11-525

**Published:** 2010-09-28

**Authors:** Manuel D Ospina-Giraldo, John G Griffith, Emma W Laird, Christina Mingora

**Affiliations:** 1 29 Kunkel, Biology Department, Lafayette College, Easton, PA 18042, USA

## Abstract

**Background:**

Enzymes involved in carbohydrate metabolism include Carbohydrate esterases (CE), Glycoside hydrolases (GH), Glycosyl transferases (GT), and Polysaccharide lyases (PL), commonly referred to as carbohydrate-active enzymes (CAZymes). The CE, GH, and PL superfamilies are also known as cell wall degrading enzymes (CWDE) due to their role in the disintegration of the plant cell wall by bacterial and fungal pathogens. In *Phytophthora infestans*, penetration of the plant cells occurs through a specialized hyphal structure called appressorium; however, it is likely that members of the genus *Phytophthora *also use CWDE for invasive growth because hyphal forces are below the level of tensile strength exhibited by the plant cell wall. Because information regarding the frequency and distribution of CAZyme coding genes in *Phytophthora *is currently unknown, we have scanned the genomes of *P. infestans, P. sojae*, and *P. ramorum *for the presence of CAZyme-coding genes using a homology-based approach and compared the gene collinearity in the three genomes. In addition, we have tested the expression of several genes coding for CE in cultures grown *in vitro*.

**Results:**

We have found that *P. infestans, P. sojae *and *P. ramorum *contain a total of 435, 379, and 310 CAZy homologs; in each genome, most homologs belong to the GH superfamily. Most GH and PL homologs code for enzymes that hydrolyze substances present in the pectin layer forming the middle lamella of the plant cells. In addition, a significant number of CE homologs catalyzing the deacetylation of compounds characteristic of the plant cell cuticle were found. In general, a high degree of gene location conservation was observed, as indicated by the presence of sequential orthologous pairs in the three genomes. Such collinearity was frequently observed among members of the GH superfamily. On the other hand, the CE and PL superfamilies showed less collinearity for some of their putative members. Quantitative PCR experiments revealed that all genes are expressed in *P. infestans *when this pathogen grown *in vitro*. However, the levels of expression vary considerably and are lower than the expression levels observed for the constitutive control.

**Conclusions:**

In conclusion, we have identified a highly complex set of CAZy homologs in the genomes of *P. infestans, P. sojae*, and *P. ramorum*, a significant number of which could play roles critical for pathogenicity, by participating in the degradation of the plant cell wall.

## Background

*Phytophthora infestans*, the causal agent of potato late blight, and as such, a major contributor to the Irish Potato Famine, is believed to cause annual losses worldwide surpassing $6.8 billion. This is a conservative estimate corresponding to the loss of approximately 15% of the global production of potato (20 Mha), valued about $45.4 billion [1]. Currently, no single strategy is successful at controlling late blight; hence, integrated management is presently the most reliable approach for disease control [2]. The apparent resilience of *P. infestans *to typical control measures such as the fungicide metalaxyl [3-7] or new approaches such as the use of resistance genes [8-10] was observed especially after the A2 mating type became more prevalent, facilitating sexual reproduction and recombination, and leading to an increase of the genetic diversity of *P. infestans *populations [11]. The ability to overcome control alternatives that had been successful previously is probably due to *P. infestans' *genome plasticity, as evidenced by the presence of multiple isozyme genotypes in sexual populations [12]. Sequence analysis of the gene coding for glucose 6 phosphate isomerase (GPI) in multiple *P. infestans *isolates revealed a widespread allelic assortment and the gene copy number differed in several isolates [13]. The evolutionary variation of the GPI genetic profile seemed to be concomitant with the emergence of more virulent *P. infestans *races [13]. Evidence indicative of *P. infestans*'s ability to undergo genome-wide changes and adaptations was originally reported by Torto et al [14], who identified a large number of secreted proteins from EST data sets, some of which could influence the infection process and manipulate the host plant response to the pathogen.

The genome of *P. infestans *has been sequenced recently and analyses of the complete sequence (~240 Mb) suggest the presence of two distinctive regions, the largest one (~ 74% of the genome) characterized by the presence of highly repetitive DNA. In contrast, in *P. sojae *and *P. ramorum*, two other major phylogenetic clades of the genus *Phytophthora*, only 39 and 28% of their respective genomes are repeated sequences [15]. The regions with highly repeated content lack conservation in the gene order and their gene density is very low. On the contrary, regions with less repetitive DNA have a high gene density and the gene order is very conserved. In general, a significant portion of the *P. infestans *genome is populated by transposons, and when compared to the other two genomes, the expansion in *P. infestans *can be attributed to the proliferation of these elements and other repeats in areas lacking gene order conservation [15]. An important group of genes found in the repeat-rich regions codes for effectors, which are proteins that can alter the course of disease. This group includes genes formerly known as avirulence genes, such as Avr3a and Avr4. A large collection of effector proteins is characterized by the presence of the RXLR-dEER motif in their sequence and recent analysis indicates that more than 500 putative RXLR-containing genes could be found in the *P. infestans *genome [15]. These effectors are thought to be translocated to the plant cell cytoplasm via haustoria, which are specialized absorbing organs that develop at the tip of the penetration peg, invaginating the host cell plasma membrane [16]. The RXLR motif has been shown to be required for the translocation of these effectors into the plant cell but not for targeting into *P. infestans *haustoria or secretion to the extra-haustorial matrix [17]. A second group of necrosis-inducing effectors, initially identified through bioinformatic approaches [14], was highly represented in the *P. infestans *genome. These cytoplasmic effectors, known as Crinkler or CRN, exist as part of a numerous family of almost 200 members, which in comparison to *P. sojae *and *P. ramorum*, is also considerably expanded [15]. In most cases, effectors are predicted to contain a typical N-terminal signal peptide [14,18].

While the frequency and distribution of effector genes in the *P. infestans *genome has been clearly elucidated, similar characteristics have not been established for most genes involved in specific metabolic processes. For example, carbohydrate-active enzymes (also known as CAZy enzymes or CAZymes) are involved in the biosynthesis and degradation of glycoconjugates, oligo- and polysaccharides. Most importantly, carbohydrate-active enzymes play a central role in the synthesis and breakdown of the plant cell wall. *Phytophthora infestans*, having a cell wall that includes cellulose as a major component presumably requires a complement of CAZymes that can greatly exceed that of fungi. In addition, *P. infestans *CAZymes can effectively function as pathogenicity factors by specifically targeting the carbohydrates of the plant cell wall. Thus far, however, a comprehensive analysis of the CAZyme-coding gene complement in *Phytophthora *is still missing.

Four superfamilies of CAZymes have been recognized: Carbohydrate esterases (CE), Glycoside hydrolases (GH), Glycosyl transferases (GT), and Polysaccharide lyases (PL) [19]. In fungi, there is abundant evidence suggesting that the GH, PL, and CE superfamilies may act as cell wall-degrading enzymes (CWDE), and therefore, play a role in pathogenicity [20,21]. In *P. infestans *exclusively, a few reports also support the notion that CWDE-coding genes exist in its genome [22-25] and that some CWDE produced by this oomycete are actually secreted [26,27]. In this report, we present a description of the CAZyme-coding genes, with emphasis on the CWDE, found in the *P. infestans*, *P. ramorum *and *P. sojae *genomes, and a preliminary analysis of the transcriptional activity of some of these genes in *P. infestans *cultures grown *in vitro*. In addition, we present a comparative analysis of *P. infestans *CAZy gene repertoire with the respective homologs found in the *P. ramorum *and *P. sojae *genomes.

## Methods

### Isolates and culturing

*Phytophthora infestans *isolate FL-01-2, belonging to race US-8 was used in this study. Cultures were grown in Rye Agar A or B (for long-term maintenance or sporulation, respectively) [28], or Pea Broth (for mycelial growth and nucleic acid extraction).

### Nucleic acid manipulation

Genomic DNA and total RNA were extracted from *P. infestans *mycelium grown in Pea Broth [13] for ~ 2 weeks at 22°C in the dark before harvesting. Total RNA was extracted using the RNeasy Plant Mini Kit (Qiagen, Valencia, CA) following the protocol supplied by the manufacturer. Genomic DNA was extracted using the GenElute™ Plant Genomic DNA Miniprep Kit (Sigma, St. Louis, MO), following the instructions provided by the supplier. Genes and fragments of interest were cloned in pCR^®^2.1 TOPO^® ^TA cloning vector (Invitrogen, Carlsbad, CA), according to the manufacturer's instructions. Sequencing reactions were performed by GeneWiz (South Plainfield, NJ). DNA digests, and agarose gel electrophoresis were conducted according to standard procedures [29].

### Reverse transcription-PCR (RT-PCR) and quantitative PCR (qPCR)

All RT-PCR reactions were performed using 1 μg of total RNA treated with RNase-free DNase (Qiagen), SuperScript III reverse transcriptase (Invitrogen, Carlsbad, CA), and gene-specific primers. Control reactions (no template and minus reverse transcriptase) were run to ensure amplification was not due to potentially contaminating DNA. *Phytophthora infestans *actin A mRNA was used as a constitutively expressed reference gene. Primer sequences, amplicon sizes, and other pertinent data are shown in Table 1. qPCR was conducted in an iQ5 Real Time PCR Detection System (Bio-Rad, Hercules, CA) using iQ SYBR Green Supermix (Bio-Rad). Candidates for RT-PCR and qPCR were selected on the basis of significant sequence similarity as determined by BLASTP searches and the lack of EST evidence supporting their expression as indicated by the information available for each gene model. Primer pairs were designed using Primer-Blast, a combination of Primer3 and BLAST [30]. *Phytophthora infestans *genomic DNA (as a template) and the Gradient option of the iQ cycler were used to optimize the amplification efficiency of each primer pair. Melt Curve/Peak analysis, which measures the melting temperature (T*_m_*) of double stranded DNA molecules, was used to determine the number of amplified products and ensure there was no non-specific amplification. Reactions were conducted as follows: 95°C/3 min (one cycle) and 40 two-step cycles consisting of 95°C/10 s (step one) and specific annealing temperature/30 s (step two). All assays were conducted by triplicate. Amplicons were either sequenced directly or cloned prior to sequencing to confirm the expected target had been amplified. Gene expression was evaluated by determining the relative quantity (ΔC_T_) of the mRNA using the gene expression analysis software provided with the iQ5 Cycler.

**Table 1:** Gene-specific primers*

Gene	Forward primer sequence	Reverse primer sequence	**T**_**m**_	Amplicon Size
PITG_08590	CATCTGTCTGGGCAGCGTGT	TTCATCGAGACCCACGAACG	65	190

PITG_08421	ATGGCACAACAGCAATGGGA	GCCCAGCAATACCCAGCTTC	65	190

PITG_08910	TAACGGCAACGAGAATGCGA	CAGTGCTGCTGGATTCGTCC	65	188

PITG_18907	CGTGGTTCGAGTCTTGCGAC	AGCTCGCTGTTCTGCCACAC	64	184

PITG_08914	CGTCAGCTCTACGCACGATCA	TGCTTCTGCCACACAACGC	65	194

PITG_02545	AGGCTCGTGTCTTCGGTTCC	GCCCCTGCTCCTCTGTTGTT	64	190

PITG_08863	CGGCAACAAGAACGCTACCA	TTGTCGTCCCAAGGTGTCCA	64	189

PITG_08912	TCAGCCACACTTGCTACGGC	TGTTCTTGGTAGAGGCCGGG	65	197

PITG_08911	TCTTCGGCAAACTCGCTCAA	CCTTGCGGGTGGATAACGTC	64	199

PITG_07333	GAGCTGGCAAATCAGACGCA	TTCGGGTCGATGTTTTCGTG	65	185

PITG_10850	TCTGTGAAGGCAGGGACGTG	ATAACTGCACCCAGCACGCA	65	182

PITG_02504	AACGGGCAGCAGACCTATCG	GGACACATCAGCATCGGGTG	65	185

PITG_14190	TCCTGTTCAAACTCGTGCTG	TCTGGGAACTTGGGGTGTAG	60	160

PITG_07334	TGCGCTACATGTTCCTTGAC	ATGTGCTCCATCCCATTAGC	60	160

PITG_11976	GCTAAACGGAGATGGACTGC	GCGAAAAAGTAACCGTGGAG	60	111

PITG_03543	TGATCGCAGACAGCTACGTC	GAAGCGCATAGAAGTACCGC	60	174

*Carbohydrate esterase gene sequences from *Phytophthora infestans *were used to design gene-specific primers for RT-PCR and qPCR. Gene names correspond to the descriptors given in assembly 1.0 of the *P. infestans *genome.

### Data mining and bioinformatics analyses

Data mining was conducted using the assembly 1 of the *P. infestans *genome released by the Broad Institute [31] and assembly 1.1 of the *P. sojae *and *P. ramorum *genomes released by the Joint Genome Institute [32,33]. Sequences from *Arabidopsis thaliana, Aspergillus *spp., *Fusarium oxysporum, Magnaporthe grisea, Neurospora crassa, Phytophthora *spp., or *Trichoderma viride *corresponding to all families belonging to the CE, GH, GT and PL superfamilies were retrieved from the CAZy database [34]. Homologous sequences in the *P. infestans, P. sojae*, and *P. ramorum *genomes were obtained by screening the CAZy sequences against the *Phytophthora *spp. genome databases using BLASTP. Matches with a statistical significance threshold (*E *value) < 10^-5 ^were selected. Orthologous search was done using the Phylogenetic Resources for the Interpretation of Genomes, PHRINGE [35], pipeline and the gene mappings between genomes. Potential subcellular localization and the presence of putative signal peptides and other motifs were evaluated with the following algorithms: SecretomeP (non-classical and leaderless secretion of proteins), SignalP (signal peptide and cleavage sites), TargetP (subcellular location of proteins), NetNGlyc (N-linked glycosylation), NetOGlyc (O-GalNAc glycosylation), NetPhos (generic phosphorylation sites), all available at the Center for Biological Sequence Analysis [36], and big-PI Predictor (for Glycosylphosphatidylinositol -GPI lipid anchor- modification site prediction [37].

### Phylogenetic analysis

Sequence alignment was done with Clustal W [38]. No manual adjustments were introduced. Nucleotide distances were estimated by the Kimura 2-parameter model [39] and phylogenetic inference was performed by the distance-based Neighbor-joining (NJ) algorithm [40]. Bootstrap tests with 1,000 replications [41] were conducted to examine the reliability of the interior branches and the validity of the trees obtained. All phylogenetic and molecular evolutionary analyses were conducted using MegAlign (DNASTAR, Madison, WI) and *MEGA *version 4 [42].

## Results

### The CAZyome of *Phytophthora *spp

Scanning of the *P. infestans *genome for the presence of CAZyme-coding gene homologs resulted in a set of 435 sequences. The GH superfamily was the most highly represented, containing 244 homologs, distributed in 34 families. CE, GT, and PL superfamilies had each 49, 83 and 59 homologs, arranged in 8, 22, and 3 families, respectively. For each CAZy superfamily, the number of genes found and the families they belong to are shown on Table 2. In addition, 96 sequences containing carbohydrate-binding modules (a CBM is defined as a non-catalytic, contiguous amino acid sequence within a carbohydrate-active enzyme that has the ability to recognize and bind carbohydrate compounds [43]) were identified. Of the 435 putative homologs found, a total of 345 sequences had *E *values smaller than 10^-10^. A significant number of genes (123) coded for extracellular proteins (all containing a canonical signal peptide) as determined by SignalP. Furthermore, 15 other sequences were predicted to be extracellular by the SecretomeP algorithm and contained non-canonical secretion signals. Eight homologs, all predicted to be extracellular, appeared to contain a signal for GPI lipid anchoring. The largest set of predicted extracellular proteins containing signal peptide (79) or with non-canonical secretion signals (14) belong to the GH superfamily. There were 12 and 33 (19 of them with non-canonical secretion signals) potentially extracellular CE and PL members, respectively. No GT genes were predicted to code for extracellular proteins (see Additional file 1).

**Table 2:** Putative CAZy gene homologs found in the genomes of *P. infestans, P. sojae*, and *P. ramorum*

	*P. infestans*		*P. sojae*		*P. ramorum*	
***P. infestans *CAZy superfamily**	**Families**	**Homologs**	**Families**	**Homologs**	**Families**	**Homologs**

Carbohydrate esterases	8	49	3	35	5	21

Glycoside hydrolases	34	244	25	191	27	167

Glycosyl transferases	22	83	18	99	17	75

Polysaccharide lyases	3	59	3	54	3	47

Total		435 (164)*		379 (196)		310 (194)

*The number of genes for which a canonical or non-canonical secretion signal was identified is shown in parenthesis.

Frequently, the majority of members of a superfamily belonged to specific families. For example, GH families 76 and 81 had 17 members, families 3 and 95 had 20 members, and family 28 contained 21 putative genes. In the GT superfamily, families 41 and 71 had 14 and 15 putative genes, respectively. Family 4 and family 10 of the CE superfamily contained 14 and 15 putative genes, while all but three of the PL genes found belong to families 1 and 3 (18 and 39 putative genes, respectively). Supercontig1.2 appeared to contain the highest number of CAZy members (26), followed by supercontig 4 (24), supercontig 13 (20), supercontig 16 (19), and supercontig 3 (18).

In the genome of *P. sojae *a total of 379 CAZy homologs were identified. The GH superfamily was the most densely populated with 191 homologs, belonging to 25 families. CE, GT, and PL superfamilies had each 35, 99, and 54 homologs, distributed in 3, 18 and 3 families, respectively. In this group, 287 sequences had *E *values < 10^-10^. With regards to the CWDE group (280 sequences), 194 were predicted to code for extracellular proteins, with the vast majority containing a classical signal peptide. Fourteen homologs, two of them without a clear signal peptide, contained a sequence for GPI lipid anchoring (see Additional file 2).

*Phytophthora ramorum *contained 310 CAZy homologs in its genome. The GH repertoire was the most numerous, composed of 167 putative genes distributed in 27 families. CE, GT, and PL superfamilies had each 21, 75, and 47, organized in 5, 17, and 3 families, respectively. A total of 277 putative homologs had *E *values smaller than 10^-10^. Of the 235 CWDE found, 153 were predicted to encode extracellular proteins, with a considerable number of them carrying a signal peptide. Only five homologs, all with signal peptide, contained a signal for GPI lipid anchoring (see Additional file 3).

**Figure 1:**
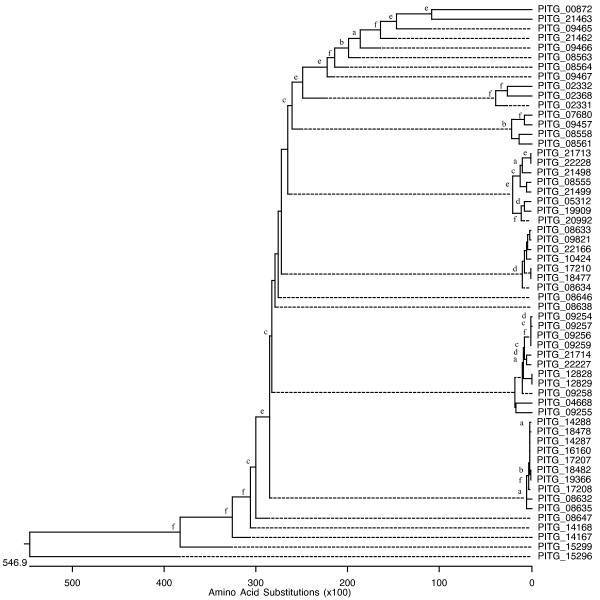
**Evolutionary relatedness of *P. infestans *PL genes**. Phylogenetic relationships among the putative PL genes found in *P. infestans *were inferred using deduced amino acid sequences. The phylogenetic trees were obtained by the Neighbor-joining method. Robustness of the trees was evaluated by a bootstrap with a 1000 replications. Not all number are shown to avoid cluttering in areas containing neighboring clades with short branches; however nodes whose existence is supported by bootstrap values greater than 60, 70, 80, 90, 95, and 99% are marked with the letters a, b, c, d, e, and f, respectively. (Trees with bootstrap numbers are available upon request).

**Figure 2:**
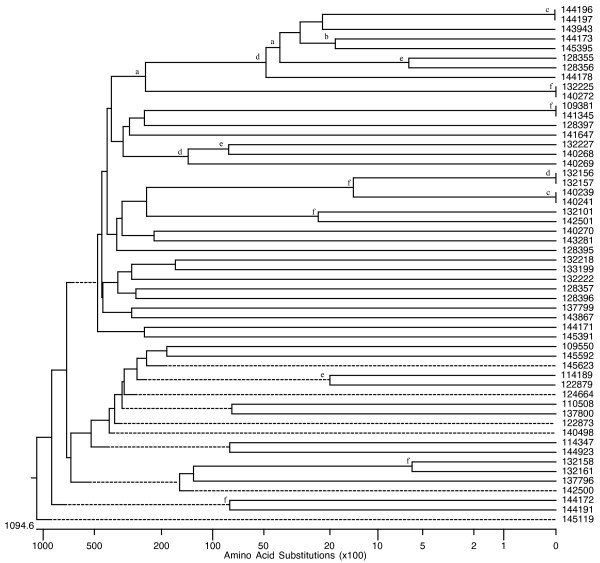
**Evolutionary relatedness of *P. sojae *PL genes**. Phylogenetic relationships among the putative PL genes found in *P. sojae *were inferred using deduced amino acid sequences. The phylogenetic trees were obtained by the Neighbor-joining method. Robustness of the trees was evaluated by a bootstrap with a 1000 replications. Not all number are shown to avoid cluttering in areas containing neighboring clades with short branches; however nodes whose existence is supported by bootstrap values greater than 60, 70, 80, 90, 95, and 99% are marked with the letters a, b, c, d, e, and f, respectively. (Trees with bootstrap numbers are available upon request).

**Figure 3:**
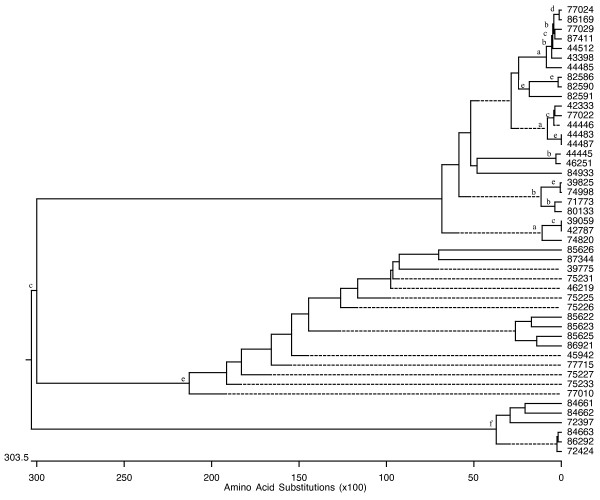
**Evolutionary relatedness of *P. ramorum *PL genes**. Phylogenetic relationships among the putative PL genes found in *P. ramorum *were inferred using deduced amino acid sequences. The phylogenetic trees were obtained by the Neighbor-joining method. Robustness of the trees was evaluated by a bootstrap with a 1000 replications. Not all number are shown to avoid cluttering in areas containing neighboring clades with short branches; however nodes whose existence is supported by bootstrap values greater than 60, 70, 80, 90, 95, and 99% are marked with the letters a, b, c, d, e, and f, respectively. (Trees with bootstrap numbers are available upon request).

Members of each superfamily have undergone multiple gene duplications as revealed by phylogenetic analyses. The phylogenetic trees of all PL genes found in the *P. infestans, P. sojae*, and *P. ramorum *genomes are shown as an example (Figures 1, 2 and 3). A Codon-based Test of Positive Selection, which provides the probability of rejecting the null hypothesis of strict-neutrality (dN = dS. dS and dN are the numbers of synonymous and non-synonymous substitutions per site, respectively) in favor of the alternative hypothesis of positive selection (dN > dS), was conducted using the *P. infestans *PL dataset. Results indicate that there is no evidence to reject the null hypothesis of strict-neutrality (*P *> 0.05).

In all three genomes, CAZy homologs not considered to be extracellular appeared to be delivered mostly to the cytoplasm, the mitochondrion, or the plasma membrane. Interestingly, a few sequences were predicted to target the nucleus. *Ab initio *prediction of intron presence in all three genomes revealed that 61.4% of the *P. infestans *CAZyme-coding genes contain introns. In *P. sojae*, 51.2% of the genes had introns, and in *P. ramorum*, there were 37.7% of intron-containing genes. The number of introns per gene ranged from one to ten and their size generally fluctuated between 40 and 200 bp, although bigger introns appeared to exist (see Additional files 1, 2, 3). A significant number of introns have been validated through expressed sequence tag (EST) comparisons. It is important to note, however, that a large set of introns remains to be confirmed, especially in the cases of genes with more than 3 introns and/or intron size greater than 200 bp. In this case, RT-PCR analysis is required to confirm whether these introns truly exist or are artifacts introduced in the original gene assemblies.

### Gene synteny and collinearity

**Figure 4:**
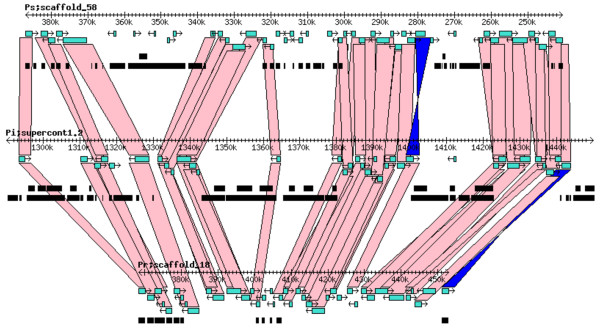
**Correspondence in chromosomal location of CAZy gene homologs among the three *Phytophthora *species**. Rectangles in turquoise represent the CAZy genes in the three genomes; connecting pink bands indicate direct collinear orthologs; blue bands connect inverted collinear orthologs.

**Figure 5:**
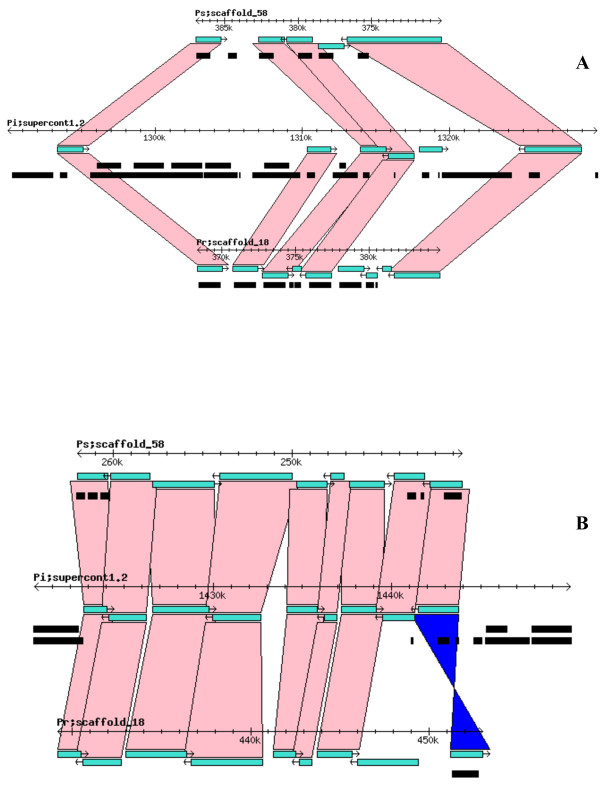
**Gene collinearity frequently observed among members of the GH superfamily**. Two different regions, of approximately 40 and 30 kb respectively, from *P. infestans *supercontig1.2 in which 6 (A) and 9 (B) GH homologs exhibit collinearity among the three genomes are shown.

**Figure 6:**
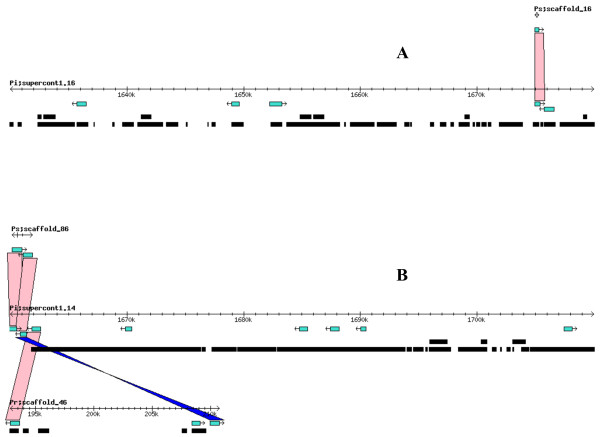
**Members of CE and PL superfamilies showed less collinearity for some of their putative members**. In the case of CE, only one (out of five) gene located in a 50-kb region of supercontig1.16 had an ortholog in a corresponding scaffold in *P. sojae *but not in *P. ramorum *(A). In the case of PL, three (out of eight) genes located in a 50-kb region of supercontig1.14 had orthologs in both *P. sojae *and *P. ramorum *(B).

Syntenic analysis of the *Phytophthora *spp. genomes, based on regions of those supercontigs (known as scaffolds in *P. sojae *and *P. ramorum*) containing the largest number of CAZy homologs revealed, in general, a high degree in location conservation (*i.e. *multiple genes found in a *P. infestans *supercontig had homologs in the same *P. sojae *or *P. ramorum *scaffold. An example of this syntenic relationship is shown in Figure 4. Gene collinearity for a specific CAZy superfamily was also apparent as indicated by the presence of multiple, sequential orthologous pairs when the *P. infestans *genome was compared with either one of the other two genomes (Figure 5a and Figure 5b). Such collinearity was frequently observed among members of the GH superfamily. On the other hand, the CE and PL superfamilies showed less collinearity for some of their putative members (Figure 6a and Figure 6b).

A preliminary search in the *P. sojae, P. ramorum*, and other oomycete genomes for orthologs to the CAZy genes found in the *P. infestans *genome using the Phylogenetic Resources for the Interpretation of Genomes (PHRINGE. http://oomycetes-public.genomeprojectsolutions-databases.com/cgi-bin/clusterSearch.pl) pipeline and the gene mappings between genomes revealed the presence of orthologous genes in the target genomes for a majority of CAZy members (Table 3). On a few occasions, orthologs to the *P. infestans *CAZy genes were found in the *P. sojae *genome only; more rarely, orthologs appear to exist in *P. ramorum *but not in *P. sojae*. Less frequently, potential orthologs could also be identified in the genomes of *Hyaloperonospora arabidopsidis *and *Pythium ultimum. *All, but one, of the CE homologs had orthologs in both *P. sojae *and *P. ramorum *genomes, and a total of 17 putative CE-coding genes appeared to have paralogs within the *P. infestans *genome (see Additional file 4). Two hundred fourteen GH-coding genes had orthologs in both *P. sojae *and *P. ramorum*. Three putative GH-coding genes had an ortholog only in *P. ramorum*, while seven other had an ortholog in *P. sojae *only. The remaining 20 genes did not appear to have orthologs in either genome. In addition, due to duplications in the target genomes, several *P. infestans *genes seemed to have multiple co-orthologs, mostly in the *P. sojae *genome (see Additional file 5). Ninety-five of the GH-coding genes appeared to have at least one paralog in the *P. infestans *genome. Of the GT complement, 72 members had orthologs in both, *P. sojae *and *P. ramorum *genomes while three had orthologs in *P. sojae *only. Only 16 of members of this group appeared to have paralogs in the *P. infestans *genome (see Additional file 6). A total of 56 (out of 59) *P. infestans *PL-coding genes had orthologs either in both, *P. sojae *and *P. ramorum *(53), or in *P. ramorum *only (3) (see Additional file 7). Forty-eight members of this CAZy superfamily exhibited paralogs in *P. infestans *(Table 3).

**Table 3:** Orthologs and paralogs

*P. infestans *CAZy superfamily	Paralogs	Orthologs in *P. sojae *and *P. ramorum*	Orthologs in *P. sojae *only	Orthologs in *P. ramorum *only	*P. infestans *homologs with no match
Carbohydrate esterases	17	48	0	0	1

Glycoside hydrolases	95	214	7	3	20

Glycosyl transferases	16	72	3	0	8

Polysaccharide lyases	48	53	0	3	3

Total	176	387	10	6	32

Number of CAZy paralogs found in *P. infestans *and total number of orthologs found in either *P. sojae *or *P. ramorum *(or both genomes). Orthologs and paralogs were determined using PHRINGE and the gene mappings between genomes available from the Broad Institute.

### Expression analysis of a subset of CE-coding genes

Sixteen putative CE-coding genes for which no EST evidence was available in the databases, were analyzed for expression using cDNA templates derived from total mycelial RNA extracted from *P. infestans *grown *in vitro *(Table 4). RT-PCR experiments allowed us to detect transcripts for seven of the CE-coding genes studied and visual inspection indicated that presumably some of these transcripts were being expressed at different rates. In order to quantify the differences of expression suggested by the RT-PCR assays, we designed real-time qPCR experiments for all genes, including those that did not appear to be expressed *in vitro*. These qPCR assays were performed using three technical replicates for each CE-coding gene and for actin A, the constitutive control; a no-template control reaction was also included. In order to minimize the effect of potential pipetting errors and achieve meaningful results, only trials in which individual replicate values had standard deviation < 0.5 were used to determine the mean threshold cycle (T_c_) for each gene and for subsequent analyses. A comparison between the expression levels of all CE genes and that of the constitutive control actin A revealed that all CE genes were expressed at lower levels than actin A, with relative fold expressions (RFE) fluctuating between 0.00010 and 0.21440. Based on the RFE for each gene, four different groups could be formed: maximum expression (Group 1: RFE > 0.04), medium expression (Group 2: RFE = 0.003 - 0.039), minimal expression (Group 3: RFE = 0.001 - 0.0029), and very low expression (Group 4: RFE < 0.001). Results of the qPCR experiments indicate that transcripts from genes PITG_02545, PITG_03543, and PITG_08912 accumulated at a higher rate than any other transcript, as determined by their threshold cycle and RFE. Four genes (PITG_07734, PITG_08421, PITG_10850, and PITG_11976) had medium levels of expression and another group of four genes (PITG_08863, PITG_08914, PITG_14190, and PITG_18907) showed minimal levels of expression; the remaining 5 genes had expression rates barely above background levels. Overall, the expression profiles, as measured by qPCR, matched those observed by RT-PCR. The only exceptions were PITG_08912 and PITG_03543, which had not been clearly detected by RT-PCR.

**Table 4:** Expression of CE genes

Expression group	Gene	RFE
4	PITG_02504	0.0001 (± 0.0000)

1	PITG_02545	0.0438 (± 0.0025)

1	PITG_03543	0.1224 (± 0.0309)

4	PITG_07333	0.0002 (± 0.0000)

2	PITG_07334	0.0039 (± 0.0008)

2	PITG_08421	0.0035 (± 0.0009)

4	PITG_08590	0.0005 (± 0.0004)

3	PITG_08863	0.0010 (± 0.0003)

4	PITG_08910	0.0003 (± 0.0001)

4	PITG_08911	0.0005 (± 0.0003)

1	PITG_08912	0.1686 (± 0.0432)

3	PITG_08914	0.0006 (± 0.0000)

2	PITG_10850	0.0242 (± 0.0065)

2	PITG_11976	0.0105 (± 0.0007)

3	PITG_14190	0.0015 (± 0.0002)

3	PITG_18907	0.0008 (± 0.0003)

Relative fold expression (RFE) of *P. infestans *CE genes in vegetative non-sporulating mycelium from *in vitro *cultures, compared to expression of actin A. Values correspond to the average of three reactions. The standard deviation is given in parenthesis.

## Discussion

The complexity of carbohydrate metabolism is in part explained by the extensive assortment of carbohydrate compounds and their diverse stereochemistry. Catalysis of the biochemical reactions involving these compounds, therefore, requires a vast array of enzymes, now referred to as "carbohydrate-active enzymes" (CAZymes). Four CAZyme superfamilies of structurally related enzymes that degrade, modify, or create glycosidic bonds are known: Carbohydrate esterases (CE), Glycoside hydrolases (GH), Glycosyl transferases (GT), and Polysaccharide lyases (PL). Since most of these enzymes target carbohydrates that are part of the plant cell wall, they are also referred to as cell wall-degrading enzymes (CWDE). Analysis of the genomes of *P. infestans *and two other *Phytophthora *spp. has revealed that these organisms contain a large multiplicity of CAZymes (Table 2). Most of these enzymes are unequivocally involved in the biochemical pathways aimed at maintaining *Phytophthora *metabolism. The fact that a significant number of these CAZymes contain carbohydrate-binding modules, which allows them to recognize and bind carbohydrate compounds [43], would support this assessment. Moreover, the presence of relatively large complement of CAZy genes with predicted canonical or non-canonical secretion signals, which would enable them to effectively function as CWDE, would seem to indicate that these oomycetes also rely on enzymatic activities to successfully infect and colonize their hosts. Surprisingly, despite its greater number of CAZy homologs, *P. infestans *contains a smaller set of extracellular proteins than *P. sojae *or *P. ramorum*. The GH superfamily, with 115 families currently recognized based on their amino acid sequence [all of which catalyze the hydrolysis of a glycosidic bond between two or more carbohydrates or between a carbohydrate and a non-carbohydrate moiety [44-46]], is the most highly represented in the *P. infestans, P. sojae*, and *P. ramorum *genomes. Given the complexity of carbohydrate biochemistry and the broad range of hydrolytic activities it involves, is not unexpected that all genomes examined exhibit a considerable number of GH members. A number of these genes lack a cellulose-binding domain (CBD), a characteristic that was originally noted in the first GH family 5 gene cloned from a phytopathogenic fungus [47,48]. The CBD anchors the enzyme to crystalline cellulose substrates and it has been suggested that its absence would, therefore, facilitate diffusion of the enzyme through the host cell wall [22]. Within the GH group, the family with the largest number of members was family 28, which is comprised of enzymes with multiple, but related, functions (E.C. 3.2.1.*). These are mostly polygalacturonases (PGs) associated with the hydrolysis of galacturonic acid-based compounds, which are usually found as part of pectate and other galacturonans. PGs are believed to play a major role in the degradation of the plant cell wall by fungi through the hydrolysis of the pectin layer, which facilitates tissue invasion and maceration. Although large families of PG-coding gene have been characterized in *P. cinnamomi *[49] and *P. parasitica *[50] and individual PG-coding genes have been cloned and characterized from *P. infestans *[25], this is the first comprehensive report on this group of genes in *P. infestans*. PG genes are expressed *in planta*, and at least in the case of *P. parasitica*, their expression has been clearly linked to pathogenicity [50]. GH families 3 and 95 had 20 members; giving the biochemical activities of the former (mainly β-glycosidase, which chiefly targets the hydrolysis of terminal, non-reducing β-D-glycosyl residues, releasing β-D-glucose) the number of gene copies is not unusual. In contrast, the relatively high extent of the latter family is puzzling, as its key role is the hydrolysis of fucose derivatives, which in comparison with other carbohydrates are less abundant in the plant or oomycete cell. Intriguingly, fucosterol (a sterol with a fucose moiety) is the most prominent sterol found in oomycetes capable of synthesizing sterols *de novo*; however, so far there is no evidence that *Phytophthora *is among such organisms. Therefore, the presence of this large complement of putative proteins with α-fucosidase activity remains difficult to explain. Finally, GH families 76 and 81, which are involved in the random hydrolysis of (1→6)-α-D-mannosidic linkages in unbranched (1→6)-mannans and the hydrolysis of (1→3)-β-D-glycosidic linkages in (1→3)-β-D-glucans, respectively, were also highly represented. Mannans are found in the fungal cell wall, but more importantly, are present in all lineages of land plants analyzed to date, where they are key constituents of the cell wall and play major roles as carbohydrate storage compounds and in metabolic networks devoted to other cellular processes [51] and β-1,3- and β-1,6-glucans compose the bulk of the oomycete cell wall but are also components of the plant cell. Hence, the presence of multiple members of these families could confer a significant evolutionary advantage to *P. infestans.*

Although the number of putative GT members was considerably large, this was not surprising as the transfer of sugar moieties resulting in the formation of glycosidic bonds during the biosynthesis of disaccharides, oligosaccharides and polysaccharides involves the action of hundreds of different glycosyl transferases [52]. Members of family 41 (one of the two most highly represented GT families in *P. infestans*) catalyze the glycosylation of proteins at asparagine residues while members of family 71 (E.C. 2.4.1*) have, in general, α-mannosyltransferase activity (but more than 200 specific activities can be found within this enzyme class). Perhaps, the most interesting members found within the GT superfamily are the four cellulose synthase genes (GT family 2), which represent a novel class and whose function has been recently characterized in depth [53]. These genes are required for pathogenicity as evidenced by *P. infestans *inability to form functional appressoria when the genes were silenced through RNA interference [53]. Even more intriguing is the presence of a fifth member of the GT family 2 that matches very strongly (*E *value = 0) the putative chitin synthase gene from *Magnaporthe grisea*. Although no direct evidence exists favoring the presence of chitin in *P. infestans *cell wall, the existence of chitin synthase genes has been demonstrated in other oomycetes [54,55], suggesting that chitin is indeed produced in these species. We have cloned this gene from both *P. infestans *and *P. sojae *and preliminary experiments indicate that the gene is expressed in cultures grown *in vitro*. Results from functional characterization assays will be published elsewhere.

For *Phytophthora *pathogenesis, however, the presence of multiple PL and CE putative members is highly significant, as both types of enzymes are involved in the degradation of cell wall components either by cleavage of the polysaccharide chains, which leads to the formation of a double bond at the resulting non-reducing end, or by catalysis of the acyl group removal from substituted saccharides, respectively [56]. All but a very few of the PL genes found belong to families 1 and 3 (18 and 39 putative genes in *P. infestans*, respectively), and almost 40% of these putative genes have canonical or non-canonical secretion signals. Both of these families are involved in the degradation of the plant cell's middle lamella, either by hydrolysis of pectate (families 1 and 3) or its methyl ester, pectin (family 1). Families 4 and 10 of the CE superfamily have the largest numbers of members in *P. infestans*. The putative CE family 4 genes found (14) act on deacetylation of xylans, chitin, and peptidoglycans while the family 10 members (15) are esterases acting on non-carbohydrate substrates. Previous reports indicate that the cutinase gene family has undergone a notable expansion in *P. sojae *and *P. ramorum *[57]. Members of this family (CE family 5) hydrolyze cutin, a polymer of hydroxy fatty acids that are usually C_16 _or C_18 _and contain up to three hydroxy groups. In *P. infestans*, the actual set of genes with true cutin hydrolase activity (E.C. 3.1.1.74) is equal to *P. ramorum's *(4) but relatively small compared to the number of these genes found in *P. sojae *(16). It is worth noting that, so far, no conclusive evidence has been found linking *Phytophthora *pathogenicity with cutinase activity.

Phylogenetic analysis for each superfamily suggests a very active evolutionary history characterized by constant duplications (Figures 1, 2 and 3). Two major clades, in which most PL genes are contained, can be seen in the phylogenetic tree for the PL superfamily in *P. sojae *and *P. ramorum*. An even closer relationship among the PL members can be seen in the phylogenetic analysis results obtained for *P. infestans*. In this species, essentially all PL genes have evolved from a single common ancestor. Genomic comparisons conducted with other oomycete genomes including *P. sojae, P. ramorum, Hyaloperonospora arabidopsidis *and *Pythium ultimum *indicate that homologs for a large number of CAZyme-coding genes exist in all oomycete species studied; however, it is plausible that the fast evolutionary pace shown by the *P. infestans *genome has led to the appearance of a few unique genes for which no homologs have been found elsewhere (Table 3). We used a cut-off value of 10^-5 ^to determine homology by BLASTP; however, even when a smaller cut-ff (10^-10^) was used a large set of potential homologs was found (in *P. infestans *this equals to only 90 less sequences than with the higher cut-off). This would support the validity of the results obtained using this method.

The nature of the plant cell wall, and the fact that cell walls constitute the fundamental tier where plant-pathogen interactions take place would help explain the need for a multiplicity of CAZymes in *Phytophthora*. In the primary cell wall, the cellulose microfibrils and the hemicellulose [this term applies to all glycans extracted from the cell wall, to which the cellulose microfibrils are non-covalently bound, including xyloglucans (XyGs) and glucuronoarabinoxylans (GAXs)], are embedded in a pectin matrix. Both, XyGs and GAXs are composed of an extensive variety of modified and non-modified carbohydrate monomers [58]. As the cell wall is the first barrier that must be breached in order to penetrate and successfully colonize the host, it is plausible that an abundant assortment of enzymes targeting the glycosidic bonds be produced by the pathogen. In addition, these enzymes could also be associated with the necrotrophic phase [59]. The structure and composition of *P. infestans *cell wall is still ill defined; however, it is clear that cellulose is one of its major components (as opposed to chitin, which is the major component of fungal cell walls [60]). Therefore, the overall carbohydrate metabolism and the specific chemical activities needed for pathogenicity would help explain the vast array of genes coding for CAZymes found in these three genomes.

True synteny is loosely defined as a correspondence in the actual chromosomal locations of two gene homologs from two related species [61]. Despite the gross and small-scale chromosomal rearrangements typical of fungal genomes, which can vary by more than an order of magnitude both within and between kingdoms [62], *P. infestans, P. sojae*, and *P. ramorum *appear to have retained many structural similarities in their chromosomes (Figure 4). Previously, extensive collinearity between orthologs from *P. sojae *and *P. ramorum *had been reported [59]. Whereas it is not possible to ascertain the actual level of synteny among the three *Phytophthora *genomes given that individual chromosomes were not sequenced, the presence of multiple, collinear homologs in every scaffold or supercontig from each genome would indicate that, in most cases, they all share a very similar chromosomal arrangement (Figure 5). A few CE and PL genes appear to be the exceptions (Figure 6). Determining the true ortholog of a gene is a challenging task given that sequences evolve at different rates and duplications and losses are fairly common; in some cases, orthologs are 100% identical and in other cases there is no detectable sequence similarity. This makes the use of distance measuring methods insufficient to determine orthology (Jeffrey Boore, pers. comm.). For this reason, in addition to using the gene mappings between genomes, we also looked at the evolutionary trees generated by PHRINGE, which provide the actual evolutionary history of the genes and facilitate the accurate determination of orthology by analyzing gene duplications and losses. Most instances of orthology were validated by both methods; however, in several cases, potential orthologs found in the gene mappings were not validated by PHRINGE and vice versa. Even within PHRINGE, while numerical values (low seed score) could suggest orthology, the phylogenetic tree did not support such relationship. Moreover, there were cases in which seed score was high but the phylogenetic tree appeared to support orthology. Interestingly, in the GH and GT superfamilies, the number of potential orthologs in the target genomes (when present) was usually one, and only in a few situations there appeared to be more than one ortholog for a gene. In contrast, most *P. infestans *CE and PL genes had more than one (usually up to four) orthologs in both *P. sojae *and *P. ramorum *genomes. The overall expansion of these gene families in each species is evidenced by the number of paralogs found, especially within the GH and PL superfamilies, with the latter showing an extremely high percentage of members (81.4%) with paralogous genes (Table 3 and Figures 1, 2 and 3).

For a large number of homologs identified there is EST evidence that suggests they are expressed *in vitro*. However, a considerable group of gene models in all genomes still lacks any evidence of expression. Therefore, we designed specific primers targeting distinctive regions of these genes using a Clustal-based sequence alignment, as a starting point to select the most dissimilar regions, and Primer-Blast. We attempted to design primers that would span an intron, but frequently this was not possible due to the fact that many *P. infestans *genes do not contain introns. In addition, these primers were intended for both RT-PCR and qPCR use, but the more stringent primer design constraints of the latter technique became a limiting factor, making it difficult to design primers that would successfully work in both types of assays. Using RT-PCR, we were able to detect seven of the 16 genes targeted. Because of its greater sensitivity and the need to quantify the differences in expression rates, we evaluated the same genes using qPCR. When determining the mean threshold cycle (T_c_) only trials in which individual replicate values had standard deviation (S_D_) < 0.5 were used; this would minimize the effect of potential pipetting errors. Hence, on several occasions multiple trials were run in order to obtain consistent values that were reliable for further analyses. Because we used equal amounts of starting material for each qPCR experiment, we were able to use the Relative Quantity (ΔC_T_) method of analysis, in which no modification of the data is needed to obtain normalized data. Clearly, in comparison to actin A, all CE genes were expressed at much lower levels than actin A, and the relative quantity and fold expression of the majority of genes was below 0.005. Although all genes could be detected, there were apparent differences in expression as suggested by the RFE and the ΔC_T _method of analysis. Two of the genes that were expressed at the highest rate (PITG_02545 and PITG_08912) belong to CE family 8, whose known activity is pectin methylesterase. The third one (PITG_03543) has pectin acetylesterase activity (family 13). Interestingly, in all genomes studied, there is a considerably high number of CE family 8 gene copies. This result matches our expectations very well as pea seeds constitute the main nutritional component of growth medium and these combined activities would be required for its utilization. The other genes analyzed have various esterase activities but may not be essential for *in vitro *growth. We are in the process of evaluating by qPCR the expression of all CAZyme gene models for which there is no EST evidence or that contain questionable intron size or a number of introns exceeding the usual number typically found in oomycetes.

## Conclusions

In conclusion, we have identified a highly complex set of CAZy homologs in the genomes of *P. infestans, P. sojae*, and *P. ramorum*, a significant number of which could play roles critical for pathogenicity, by participating in the degradation of the plant cell wall. For most of these genes there are homologs in the three species, distributed and organized in patterns that strongly support the existence of synteny and gene collinearity among these species. Preliminary experiments with highly specific gene primers (as shown by the results of the Melt Curve/Peak assays run during qPCR analysis) indicate that all these genes are expressed in cultures grown *in vitro*, albeit at different rates.

## Authors' contributions

MOG conceptualized and designed the research project, acquired and analyzed data, and drafted manuscript. JJG, EWL, and CM carried out homology-based searches and conducted synteny analysis and orthologous gene search. EWL and CM performed expression analyses. All authors read and approved the final manuscript.

## Supplementary Material

Additional file 1 **CAZy genes in *P. infestans***. CAZyme-coding homologs in *P. infestans *organized by their respective CAZy superfamily. ^1^Numbers represent intron sizes; "multiple" refers to the fact that more than 9 introns were present in the gene model. ^2^Cellular localization predicted by SecretomeP, SignalP, or TargetP algorithms. ND, not determined. Click here for file

Additional file 2 **CAZy genes in *P. sojae***. CAZyme-coding homologs in *P. sojae *organized by their respective CAZy superfamily. ^1^Numbers represent intron sizes; "multiple" refers to the fact that more than 9 introns were present in the gene model. ^2^Most likely cellular localization predicted by SecretomeP, SignalP, or TargetP algorithms. ND, not determined. Click here for file

Additional file 3 **CAZy genes in *P. ramorum***. CAZyme-coding homologs in *P. ramorum *organized by their respective CAZy superfamily. ^1^Numbers represent intron sizes; "multiple" refers to the fact that more than 9 introns were present in the gene model. ^2^Most likely cellular localization predicted by SecretomeP, SignalP, or TargetP algorithms. ND, not determined. Click here for file

Additional file 4 ***Phytophthora *CE orthologs**. *Phytophthora *CE orthologs as determined using the Phylogenetic Resources for the Interpretation of Genomes (PHRINGE). All, but one, of the CE homologs had orthologs in both *P. sojae *and *P. ramorum *genomes. In most cases, more than one orthologous gene was found in each of the three *Phytophthora *species. Click here for file

Additional file 5 ***Phytophthora *GH orthologs**. *Phytophthora *GH orthologs as determined using the Phylogenetic Resources for the Interpretation of Genomes (PHRINGE). In most cases, more than one orthologous gene was found in each of the three *Phytophthora *species. Two hundred fourteen GH-coding genes had orthologs in both *P. sojae *and *P. ramorum*. Three putative GH-coding genes had an ortholog only in *P. ramorum*, while seven other had an ortholog in *P. sojae *only. The remaining 20 genes did not appear to have orthologs in either genome. Click here for file

Additional file 6 ***Phytophthora *GT orthologs**. *Phytophthora *GT orthologs as determined using the Phylogenetic Resources for the Interpretation of Genomes (PHRINGE). In most cases, more than one orthologous gene was found in each of the three *Phytophthora *species. A total of 72 members had orthologs in both, *P. sojae *and *P. ramorum *genomes while three had orthologs in *P. sojae *only. Click here for file

Additional file 7 ***Phytophthora *PL orthologs**. *Phytophthora *PL orthologs. *Phytophthora *PL orthologs as determined using the Phylogenetic Resources for the Interpretation of Genomes (PHRINGE). In most cases, more than one orthologous gene was found in each of the three *Phytophthora *species. A total of 56 (out of 59) *P. infestans *PL-coding genes had orthologs either in both, *P. sojae *and *P. ramorum *(53), or in *P. ramorum *only (3). Click here for file
