# Supplementary material for: The CAZyome of Phytophthora spp.: A comprehensive analysis of the gene complement coding for carbohydrate-active enzymes in species of the genus Phytophthora
Source: BMC Genomics. 2010 Sep 28;11:525. doi: 10.1186/1471-2164-11-525 (PMC2997016; doi:10.1186/1471-2164-11-525)
Supplement: Additional file 1 — CAZy genes in P. infestans. CAZyme-coding homologs in P. infestans organized by their respective CAZy superfamily. 1Numbers represent intron sizes; "multiple" refers to the fact that more than 9 introns were present in the gene model. 2Cellular localization predicted by SecretomeP, SignalP, or TargetP algorithms. ND, not determined. [file 1471-2164-11-525-S1.PDF]

| CAZy Group | Family | Gene Identifier | E Value | Introns[1] | Predicted Cellular Localization[2] |
|---|---|---|---|---|---|
| Carbohydrate Esterase | 1 | PITG_06891.1 | 0.00E+00 | 62 | cytoplasm; mitochondria |
| Carbohydrate Esterase | 2 | PITG_04874.1 | 1.81E-12 | 70, 70, 70, 70 | extracellular |
| Carbohydrate Esterase | 4 | PITG_01502.1 | 6.74E-15 | 60, 90, 45, 90, 80, 65, 65 | cytoplasm; nucleus |
| Carbohydrate Esterase | 4 | PITG_02274.1 | 2.96E-06 | 60, 50, 50 | cytoplasm |
| Carbohydrate Esterase | 4 | PITG_02483.1 | 5.04E-06 | 60, 95 | plasma membrane |
| Carbohydrate Esterase | 4 | PITG_02607.1 | 2.42E-08 | 70, 80, 70 | cytoplasm |
| Carbohydrate Esterase | 4 | PITG_02899.1 | 7.52E-10 | 55, 90, 55 | cytoplasm |
| Carbohydrate Esterase | 4 | PITG_02902.1 | 7.02E-16 | 90, 95, 295 | cytoplasm |
| Carbohydrate Esterase | 4 | PITG_03463.1 | 2.26E-14 | | cytoplasm; mitochondria |
| Carbohydrate Esterase | 4 | PITG_05542.1 | 3.99E-11 | 80, 70, 80 | cytoplasm; mitochondria |
| Carbohydrate Esterase | 4 | PITG_06969.1 | 9.51E-05 | 90, 80, 50, 50, 95 | extracellular |
| Carbohydrate Esterase | 4 | PITG_08421.1 | 4.56E-07 | 70, 60, 60, 95, 640, 380 | cytoplasm |
| Carbohydrate Esterase | 4 | PITG_08590.1 | 7.26E-31 | 45 | extracellular |
| Carbohydrate Esterase | 4 | PITG_17768.1 | 9.19E-08 | | plasma membrane |
| Carbohydrate Esterase | 4 | PITG_19054.1 | 1.73E-06 | 80, 70, 80 | cytoplasm; mitochondria |
| Carbohydrate Esterase | 4 | PITG_20223.1 | 2.30E-22 | 116 | cytoplasm; mitochondria |
| Carbohydrate Esterase | 5 | PITG_12361.1 | 2.07E-21 | | extracellular |
| Carbohydrate Esterase | 5 | PITG_12401.1 | 3.77E-23 | | extracellular |
| Carbohydrate Esterase | 5 | PITG_12422.1 | 0.00E+00 | | ND |
| Carbohydrate Esterase | 5 | PITG_12832.1 | 3.53E-21 | | extracellular |
| Carbohydrate Esterase | 8 | PITG_00041.1 | 6.83E-11 | 80, 300 | cytoplasm; nucleus |
| Carbohydrate Esterase | 8 | PITG_01029.1 | 5.05E-15 | | extracellular |
| Carbohydrate Esterase | 8 | PITG_02545.1 | 1.24E-13 | | extracellular |
| Carbohydrate Esterase | 8 | PITG_04325.1 | 1.85E-17 | | extracellular |
| Carbohydrate Esterase | 8 | PITG_06560.1 | 2.77E-13 | | extracellular |
| Carbohydrate Esterase | 8 | PITG_08863.1 | 5.22E-12 | 60 | cytoplasm |
| Carbohydrate Esterase | 8 | PITG_08902.1 | 2.42E-17 | | plasma membrane |
| Carbohydrate Esterase | 8 | PITG_08910.1 | 6.37E-18 | 54 | cytoplasm |
| Carbohydrate Esterase | 8 | PITG_08911.1 | 6.88E-18 | 45 | cytoplasm |
| Carbohydrate Esterase | 8 | PITG_08912.1 | 2.61E-25 | | extracellular |
| Carbohydrate Esterase | 8 | PITG_08914.1 | 1.92E-14 | 59 | cytoplasm |
| Carbohydrate Esterase | 8 | PITG_18907.1 | 1.42E-17 | | cytoplasm |
| Carbohydrate Esterase | 10 | PITG_00294.1 | 7.73E-13 | 70, 65 | cytoplasm; mitochondria |
| Carbohydrate Esterase | 10 | PITG_02504.1 | 1.56E-06 | | cytoplasm; mitochondria |
| Carbohydrate Esterase | 10 | PITG_03840.1 | 5.17E-17 | 60, 390, 65, 105 | plasma membrane |
| Carbohydrate Esterase | 10 | PITG_07333.1 | 2.25E-05 | 24 | plasma membrane |
| Carbohydrate Esterase | 10 | PITG_07334.1 | 2.77E-06 | | plasma membrane |
| Carbohydrate Esterase | 10 | PITG_07354.1 | 5.43E-14 | 73 | cytoplasm |

| | | | | | |
|---|---|---|---|---|---|
| Carbohydrate Esterase | 10 | PITG_08056.1 | 1.71E-28 | 70,105,70,70,60 | cytoplasm; mitochondria |
| Carbohydrate Esterase | 10 | PITG_10850.1 | 9.37E-07 | 75, 75 | cytoplasm; mitochondria |
| Carbohydrate Esterase | 10 | PITG_10932.1 | 2.61E-08 | 60, 110, 80, 80, 70, 60 | plasma membrane |
| Carbohydrate Esterase | 10 | PITG_14190.1 | 6.72E-05 | | plasma membrane |
| Carbohydrate Esterase | 10 | PITG_14194.1 | 1.60E-06 | | cytoplasm; mitochondria |
| Carbohydrate Esterase | 10 | PITG_14206.1 | 6.08E-06 | | cytoplasm; mitochondria |
| Carbohydrate Esterase | 10 | PITG_14215.1 | 7.14E-05 | | cytoplasm; nucleus |
| Carbohydrate Esterase | 10 | PITG_14598.1 | 1.58E-05 | | cytoplasm; mitochondria |
| Carbohydrate Esterase | 10 | PITG_16930.1 | 0.00E+00 | 40, 190, 75, 290 | plasma membrane |
| Carbohydrate Esterase | 11 | PITG_11976.1 | 1.41E-24 | | cytoplasm; mitochondria |
| Carbohydrate Esterase | 13 | PITG_03543.1 | 6.94E-05 | 76 | extracellular |
| Glycoside Hydrolase | 1 | PITG_21554.1 | 2.50E-08 | 90, 70, 50 | cytoplasm; mitochondria |
| Glycoside Hydrolase | 2 | PITG_01471.1 | 0.00E+00 | 50, 70, 70, 73, 150, 40 | cytoplasm |
| Glycoside Hydrolase | 3 | PITG_02103.1 | 2.19E-22 | | extracellular |
| Glycoside Hydrolase | 3 | PITG_03135.1 | 4.71E-25 | 50, 50, 60 | extracellular |
| Glycoside Hydrolase | 3 | PITG_03136.1 | 2.86E-22 | | extracellular |
| Glycoside Hydrolase | 3 | PITG_03140.1 | 8.89E-24 | 52 | cytoplasm |
| Glycoside Hydrolase | 3 | PITG_03141.1 | 1.28E-22 | 102, 62, 270, 540, 600, 70, 62 | extracellular* |
| Glycoside Hydrolase | 3 | PITG_03213.1 | 7.29E-18 | 230, 36, 250 | cytoplasm; mitochondria |
| Glycoside Hydrolase | 3 | PITG_03411.1 | 3.74E-22 | 87 | cytoplasm |
| Glycoside Hydrolase | 3 | PITG_05066.1 | 1.89E-15 | 48, 61 | cytoplasm |
| Glycoside Hydrolase | 3 | PITG_06562.1 | 6.52E-08 | 80 | cytoplasm |
| Glycoside Hydrolase | 3 | PITG_06563.1 | 4.15E-05 | 47 | cytoplasm |
| Glycoside Hydrolase | 3 | PITG_08027.1 | 1.16E-15 | 76, 48 | cytoplasm; mitochondria |
| Glycoside Hydrolase | 3 | PITG_15478.1 | 1.68E-06 | 120, 170 | plasma membrane |
| Glycoside Hydrolase | 3 | PITG_15905.1 | 2.35E-16 | 84 | extracellular |
| Glycoside Hydrolase | 3 | PITG_16507.1 | 2.73E-14 | 41,54 43 | cytoplasm |
| Glycoside Hydrolase | 3 | PITG_16558.1 | 2.43E-13 | 125, 28, 67 | cytoplasm |
| Glycoside Hydrolase | 3 | PITG_17546.1 | 1.79E-24 | 50, 70 | cytoplasm; mitochondria |
| Glycoside Hydrolase | 3 | PITG_18069.1 | 8.06E-17 | 222, 80 | cytoplasm; mitochondria |
| Glycoside Hydrolase | 3 | PITG_20676.1 | 2.19E-22 | | extracellular |
| Glycoside Hydrolase | 3 | PITG_21433.1 | 8.22E-11 | 92 | cytoplasm |
| Glycoside Hydrolase | 3 | PITG_22095.1 | 1.68E-22 | 150, 64 | cytoplasm; mitochondria |
| Glycoside Hydrolase | 5 | PITG_05011.1 | 0.00E+00 | 67 | cytoplasm; mitochondria |
| Glycoside Hydrolase | 5 | PITG_08610.1 | 0.00E+00 | 69, 72, 75 | cytoplasm; mitochondria |
| Glycoside Hydrolase | 5 | PITG_08611.1 | 0.00E+00 | | cytoplasm; nucleus |
| Glycoside Hydrolase | 5 | PITG_08612.1 | 0.00E+00 | 65 | plasma membrane |

| | | | | | |
|---|---|---|---|---|---|
| Glycoside Hydrolase | 5 | PITG_08613.1 | 0.00E+00 | | cytoplasm |
| Glycoside Hydrolase | 5 | PITG_08998.1 | 7.44E-33 | | extracellular |
| Glycoside Hydrolase | 5 | PITG_09872.1 | 0.00E+00 | | plasma membrane |
| Glycoside Hydrolase | 5 | PITG_11993.1 | 4.82E-32 | | extracellular* |
| Glycoside Hydrolase | 5 | PITG_15576.1 | 8.01E-11 | 60, 85 | cytoplasm; mitochondria |
| Glycoside Hydrolase | 5 | PITG_16486.1 | 1.91E-12 | | ND |
| Glycoside Hydrolase | 5 | PITG_16487.1 | 0.00E+00 | 73 | extracellular |
| Glycoside Hydrolase | 6 | PITG_04077.1 | 4.40E-06 | | extracellular |
| Glycoside Hydrolase | 6 | PITG_12344.1 | 4.37E-22 | 55 | extracellular* |
| Glycoside Hydrolase | 6 | PITG_18332.1 | 5.35E-20 | | extracellular* |
| Glycoside Hydrolase | 6 | PITG_18333.1 | 3.36E-14 | 85, 90 | extracellular |
| Glycoside Hydrolase | 6 | PITG_18335.1 | 8.82E-23 | | extracellular |
| Glycoside Hydrolase | 6 | PITG_18336.1 | 2.40E-20 | 138 | extracellular |
| Glycoside Hydrolase | 6 | PITG_18337.1 | 1.01E-18 | 70 | cytoplasm |
| Glycoside Hydrolase | 6 | PITG_18338.1 | 3.24E-17 | 140, 30 | extracellular |
| Glycoside Hydrolase | 7 | PITG_06788.1 | 0.00E+00 | 600, 400 | cytoplasm; nucleus |
| Glycoside Hydrolase | 7 | PITG_10204.1 | 0.00E+00 | | extracellular |
| Glycoside Hydrolase | 7 | PITG_10714.1 | 1.27E-37 | 300, 400, 120, 40 | cytoplasm; mitochondria |
| Glycoside Hydrolase | 10 | PITG_04272.1 | 1.80E-10 | 60, 70, 80 | cytoplasm |
| Glycoside Hydrolase | 10 | PITG_04273.1 | 9.85E-17 | | cytoplasm |
| Glycoside Hydrolase | 10 | PITG_17054.1 | 5.74E-41 | 60 80 | extracellular |
| Glycoside Hydrolase | 10 | PITG_17055.1 | 0.00E+00 | 60, 70, 120 | extracellular |
| Glycoside Hydrolase | 12 | PITG_06962.1 | 0.00E+00 | 500 | cytoplasm; mitochondria |
| Glycoside Hydrolase | 12 | PITG_08944.1 | 0.00E+00 | | extracellular |
| Glycoside Hydrolase | 12 | PITG_13560.1 | 2.52E-43 | 40, 38 | extracellular |
| Glycoside Hydrolase | 12 | PITG_14060.1 | 0.00E+00 | 46 | extracellular |
| Glycoside Hydrolase | 12 | PITG_16985.1 | 1.43E-38 | 105, 95 | cytoplasm; nucleus |
| Glycoside Hydrolase | 12 | PITG_16991.1 | 0.00E+00 | | extracellular |
| Glycoside Hydrolase | 12 | PITG_16992.1 | 0.00E+00 | | extracellular |
| Glycoside Hydrolase | 12 | PITG_16995.1 | 0.00E+00 | 400, 550 | ND |
| Glycoside Hydrolase | 12 | PITG_21986.1 | 2.37E-25 | | extracellular |
| Glycoside Hydrolase | 12 | PITG_22202.1 | 2.37E-25 | | extracellular |
| Glycoside Hydrolase | 17 | PITG_00219.1 | 1.76E-11 | 110 | extracellular* |
| Glycoside Hydrolase | 17 | PITG_01604.1 | 1.18E-07 | 120, 80 | cytoplasm |
| Glycoside Hydrolase | 17 | PITG_01605.1 | 9.98E-07 | | extracellular |
| Glycoside Hydrolase | 17 | PITG_04123.1 | 4.19E-05 | | extracellular |
| Glycoside Hydrolase | 17 | PITG_04124.1 | 9.34E-05 | 62 | extracellular |
| Glycoside Hydrolase | 17 | PITG_04131.1 | 2.01E-07 | 300, 400, 300 | ND |
| Glycoside Hydrolase | 17 | PITG_04135.1 | 1.54E-07 | | extracellular |

| | | | | | |
|---|---|---|---|---|---|
| Glycoside Hydrolase | 17 | PITG_04141.1 | 1.44E-05 | | extracellular |
| Glycoside Hydrolase | 17 | PITG_04158.1 | 5.85E-07 | | extracellular |
| Glycoside Hydrolase | 17 | PITG_10028.1 | 5.64E-18 | 70 | cytoplasm |
| Glycoside Hydrolase | 17 | PITG_15980.1 | 1.30E-14 | | extracellular |
| Glycoside Hydrolase | 17 | PITG_18327.1 | 1.44E-05 | 65 | cytoplasm; mitochondria |
| Glycoside Hydrolase | 18 | PITG_01283.1 | 5.80E-12 | Multiple | cytoplasm; nucleus |
| Glycoside Hydrolase | 18 | PITG_02901.1 | 7.84E-09 | 62 | cytoplasm |
| Glycoside Hydrolase | 18 | PITG_06756.1 | 8.66E-08 | 66 | cytoplasm; mitochondria |
| Glycoside Hydrolase | 18 | PITG_11523.1 | 3.52E-09 | 64 | cytoplasm; mitochondria |
| Glycoside Hydrolase | 18 | PITG_12785.1 | 1.06E-05 | 64 | cytoplasm; mitochondria |
| Glycoside Hydrolase | 18 | PITG_20319.1 | 1.38E-21 | | cytoplasm |
| Glycoside Hydrolase | 18 | PITG_20320.1 | 2.98E-08 | | cytoplasm; mitochondria |
| Glycoside Hydrolase | 18 | PITG_20321.1 | 4.01E-21 | | cytoplasm |
| Glycoside Hydrolase | 18 | PITG_20325.1 | 4.29E-15 | 44 | cytoplasm; mitochondria |
| Glycoside Hydrolase | 19 | PITG_15644.1 | 1.54E-10 | 210, 285, 40, 120, 185 | extracellular |
| Glycoside Hydrolase | 19 | PITG_17945.1 | 9.64E-21 | | extracellular |
| Glycoside Hydrolase | 19 | PITG_17947.1 | 9.64E-21 | | extracellular |
| Glycoside Hydrolase | 28 | PITG_10255.1 | 2.75E-28 | 87 | extracellular |
| Glycoside Hydrolase | 28 | PITG_10880.1 | 1.42E-16 | 80, 70, 60 | cytoplasm |
| Glycoside Hydrolase | 28 | PITG_16822.1 | 2.75E-28 | 78 | extracellular |
| Glycoside Hydrolase | 28 | PITG_16824.1 | 2.75E-28 | | extracellular |
| Glycoside Hydrolase | 28 | PITG_16825.1 | 5.74E-26 | 65, 60 | extracellular |
| Glycoside Hydrolase | 28 | PITG_17899.1 | 8.59E-14 | 75 | cytoplasm |
| Glycoside Hydrolase | 28 | PITG_19455.1 | 2.85E-17 | 50, 95 | extracellular |
| Glycoside Hydrolase | 28 | PITG_19619.1 | 2.18E-17 | 30, 130 | extracellular |
| Glycoside Hydrolase | 28 | PITG_19620.1 | 8.55E-30 | 600 | extracellular |
| Glycoside Hydrolase | 28 | PITG_19623.1 | 1.37E-27 | 51 | extracellular |
| Glycoside Hydrolase | 28 | PITG_19624.1 | 7.49E-26 | | extracellular |
| Glycoside Hydrolase | 28 | PITG_19625.1 | 6.13E-28 | 108 | cytoplasm |
| Glycoside Hydrolase | 28 | PITG_19634.1 | 5.94E-23 | | cytoplasm |
| Glycoside Hydrolase | 28 | PITG_19636.1 | 1.85E-08 | | cytoplasm |
| Glycoside Hydrolase | 28 | PITG_19637.1 | 1.91E-21 | 100, 60 | cytoplasm; nucleus |
| Glycoside Hydrolase | 28 | PITG_19649.1 | 2.40E-32 | 120 | cytoplasm |
| Glycoside Hydrolase | 28 | PITG_19653.1 | 6.34E-25 | | cytoplasm; mitochondria |
| Glycoside Hydrolase | 28 | PITG_19985.1 | 1.72E-30 | 115 | cytoplasm |
| Glycoside Hydrolase | 28 | PITG_21245.1 | 1.12E-29 | 600 | extracellular |
| Glycoside Hydrolase | 28 | PITG_21247.1 | 1.51E-26 | | extracellular |
| Glycoside Hydrolase | 28 | PITG_21925.1 | 1.32E-22 | 35 | cytoplasm; mitochondria |
| Glycoside Hydrolase | 30 | PITG_04207.1 | 2.03E-12 | 60, 180,50 | cytoplasm; mitochondria |

| | | | | | |
|---|---|---|---|---|---|
| Glycoside Hydrolase | 30 | PITG_04254.1 | 3.46E-12 | 110, 650, 120, 300 | cytoplasm |
| Glycoside Hydrolase | 30 | PITG_04255.1 | 3.69E-22 | | extracellular |
| Glycoside Hydrolase | 30 | PITG_08000.1 | 9.09E-21 | | peroxisome |
| Glycoside Hydrolase | 30 | PITG_08001.1 | 6.52E-19 | | cytoplasm; mitochondria |
| Glycoside Hydrolase | 30 | PITG_08002.1 | 1.36E-24 | 40, 30 | plasma membrane |
| Glycoside Hydrolase | 30 | PITG_08004.1 | 3.12E-21 | 88 | plasma membrane |
| Glycoside Hydrolase | 30 | PITG_08191.1 | 5.00E-11 | | cytoplasm |
| Glycoside Hydrolase | 30 | PITG_09101.1 | 6.98E-13 | | cytoplasm; nucleus |
| Glycoside Hydrolase | 30 | PITG_17500.1 | 3.12E-21 | | extracellular |
| Glycoside Hydrolase | 30 | PITG_17501.1 | 2.92E-19 | | extracellular |
| Glycoside Hydrolase | 30 | PITG_17506.1 | 1.78E-08 | 64 | extracellular |
| Glycoside Hydrolase | 30 | PITG_17507.1 | 1.01E-19 | 56 | extracellular |
| Glycoside Hydrolase | 30 | PITG_17508.1 | 1.31E-19 | | extracellular |
| Glycoside Hydrolase | 30 | PITG_17509.1 | 1.19E-04 | 60 | cytoplasm |
| Glycoside Hydrolase | 30 | PITG_19939.1 | 2.39E-21 | 410 | cytoplasm; mitochondria |
| Glycoside Hydrolase | 31 | PITG_10846.1 | 0.00E+00 | 216 | extracellular |
| Glycoside Hydrolase | 31 | PITG_11112.1 | 2.33E-20 | | cytoplasm |
| Glycoside Hydrolase | 31 | PITG_11712.1 | 1.56E-32 | | extracellular |
| Glycoside Hydrolase | 31 | PITG_13867.1 | 1.23E-29 | | cytoplasm |
| Glycoside Hydrolase | 31 | PITG_13868.1 | 0.00E+00 | 50, 60 | extracellular |
| Glycoside Hydrolase | 31 | PITG_13873.1 | 0.00E+00 | 50 | extracellular |
| Glycoside Hydrolase | 32 | PITG_14237.1 | 7.98E-23 | | extracellular |
| Glycoside Hydrolase | 32 | PITG_14238.1 | 3.97E-14 | | extracellular |
| Glycoside Hydrolase | 32 | PITG_14243.1 | 5.54E-16 | 40, 85 | extracellular |
| Glycoside Hydrolase | 35 | PITG_01138.1 | 2.41E-21 | 66, 65, 69, 107, 56 | plasma membrane |
| Glycoside Hydrolase | 37 | PITG_06808.1 | 1.15E-25 | 52 | extracellular |
| Glycoside Hydrolase | 37 | PITG_06820.1 | 1.71E-29 | | cytoplasm; microbody; nucleus |
| Glycoside Hydrolase | 37 | PITG_21660.1 | 4.51E-30 | | cytoplasm; microbody; nucleus |
| Glycoside Hydrolase | 45 | PITG_11987.1 | 3.86E-05 | 266 | cytoplasm |
| Glycoside Hydrolase | 47 | PITG_01855.1 | 0.00E+00 | 97. 75, 71 | cytoplasm; golgi body |
| Glycoside Hydrolase | 47 | PITG_02266.1 | 4.65E-32 | 16 | cytoplasm; microbody |
| Glycoside Hydrolase | 47 | PITG_07365.1 | 0.00E+00 | 72, 84, 77 | extracellular |
| Glycoside Hydrolase | 47 | PITG_09377.1 | 1.27E-29 | 43 | cytoplasm; nucleus |
| Glycoside Hydrolase | 47 | PITG_10149.1 | 0.00E+00 | 75, 82, 61, 69, 77 | extracellular |
| Glycoside Hydrolase | 47 | PITG_11537.1 | 0.00E+00 | 73, 66, 52, 77 | extracellular |
| Glycoside Hydrolase | 53 | PITG_09906.1 | 1.78E-32 | 61 | cytoplasm; microbody; nucleus |
| Glycoside Hydrolase | 53 | PITG_09907.1 | 7.77E-20 | 77, 47 | extracellular |
| Glycoside Hydrolase | 53 | PITG_09910.1 | 1.36E-32 | | extracellular |
| Glycoside Hydrolase | 54 | PITG_01496.1 | 7.07E-08 | 51 | cytoplasm; nucleus |

| | | | | | |
|---|---|---|---|---|---|
| Glycoside Hydrolase | 63 | PITG_17592.1 | 0.00E+00 | | cytoplasm; microbody; nucleus |
| Glycoside Hydrolase | 63 | PITG_21028.1 | 3.23E-32 | 42 | cytoplasm; microbody; nucleus |
| Glycoside Hydrolase | 71 | PITG_02114.1 | 0.00E+00 | | cytoplasm; mitochondrial matrix space |
| Glycoside Hydrolase | 71 | PITG_08435.1 | 0.00E+00 | | cytoplasm; mitochondrial inner matrix |
| Glycoside Hydrolase | 71 | PITG_10399.1 | 3.97E-13 | | cytoplasm; microbody; nucleus |
| Glycoside Hydrolase | 71 | PITG_20520.1 | 2.75E-14 | 62, 92, 70, 62 | extracellular |
| Glycoside Hydrolase | 72 | PITG_01266.1 | 1.25E-21 | 60 | plasma membrane* |
| Glycoside Hydrolase | 72 | PITG_01267.1 | 1.53E-19 | | cytoplasm; mitochondria* |
| Glycoside Hydrolase | 72 | PITG_01269.1 | 3.64E-21 | 49 | cytoplasm; nucleus |
| Glycoside Hydrolase | 72 | PITG_03425.1 | 6.63E-23 | 27, 65, 79 | extracellular |
| Glycoside Hydrolase | 72 | PITG_08999.1 | 3.21E-06 | | plasma membrane |
| Glycoside Hydrolase | 72 | PITG_14124.1 | 6.42E-18 | | extracellular |
| Glycoside Hydrolase | 72 | PITG_14138.1 | 1.02E-23 | 67, 70, 68 | extracellular |
| Glycoside Hydrolase | 72 | PITG_14139.1 | 1.06E-20 | 50, 45 | extracellular |
| Glycoside Hydrolase | 72 | PITG_14140.1 | 7.58E-19 | 80, 50, 70, 60 | extracellular |
| Glycoside Hydrolase | 72 | PITG_14141.1 | 3.89E-23 | 69. 75 | extracellular |
| Glycoside Hydrolase | 72 | PITG_17496.1 | 2.28E-15 | 89 | cytoplasm |
| Glycoside Hydrolase | 72 | PITG_17497.1 | 2.20E-26 | 96 | cytoplasm; nucleus |
| Glycoside Hydrolase | 72 | PITG_22383.1 | 9.85E-06 | 180, 60, 380 | cytoplasm; nucleus |
| Glycoside Hydrolase | 75 | PITG_13052.1 | 3.91E-05 | 60, 120, 45 | extracellular |
| Glycoside Hydrolase | 76 | PITG_00972.1 | 9.44E-15 | | cytoplasm |
| Glycoside Hydrolase | 76 | PITG_08844.1 | 4.70E-06 | 30, 570 | cytoplasm |
| Glycoside Hydrolase | 76 | PITG_08846.1 | 3.04E-13 | | cytoplasm |
| Glycoside Hydrolase | 76 | PITG_08850.1 | 1.08E-10 | 180, 95, 290 | cytoplasm; mitochondria |
| Glycoside Hydrolase | 76 | PITG_08866.1 | 9.77E-12 | 28 | cytoplasm |
| Glycoside Hydrolase | 76 | PITG_08867.1 | 2.49E-15 | 680, 350, 450 | cytoplasm; mitochondria |
| Glycoside Hydrolase | 76 | PITG_08869.1 | 2.49E-15 | | cytoplasm |
| Glycoside Hydrolase | 76 | PITG_10290.1 | 3.03E-21 | | cytoplasm; mitochondria |
| Glycoside Hydrolase | 76 | PITG_10292.1 | 2.32E-29 | | cytoplasm; mitochondria |
| Glycoside Hydrolase | 76 | PITG_10293.1 | 1.31E-24 | 602 | cytoplasm; mitochondria |
| Glycoside Hydrolase | 76 | PITG_10855.1 | 1.05E-05 | 50 | cytoplasm |
| Glycoside Hydrolase | 76 | PITG_11293.1 | 4.51E-33 | | cytoplasm |
| Glycoside Hydrolase | 76 | PITG_11294.1 | 2.83E-27 | 87 | cytoplasm; mitochondria |
| Glycoside Hydrolase | 76 | PITG_11295.1 | 6.75E-21 | 90, 60 | cytoplasm; mitochondria |
| Glycoside Hydrolase | 76 | PITG_11623.1 | 7.48E-12 | 92 | cytoplasm |
| Glycoside Hydrolase | 76 | PITG_21743.1 | 5.18E-13 | | cytoplasm |
| Glycoside Hydrolase | 76 | PITG_21744.1 | 1.12E-15 | | cytoplasm |
| Glycoside Hydrolase | 78 | PITG_01760.1 | 2.46E-11 | 140, 90, 50 | extracellular |
| Glycoside Hydrolase | 78 | PITG_01765.1 | 3.23E-08 | 50 | cytoplasm |

| | | | | | |
|---|---|---|---|---|---|
| Glycoside Hydrolase | 78 | PITG_05097.1 | 0.00E+00 | 110 | extracellular |
| Glycoside Hydrolase | 78 | PITG_19230.1 | 4.80E-43 | 80, 140, 80, 50 | extracellular |
| Glycoside Hydrolase | 78 | PITG_19561.1 | 4.39E-12 | 90, 110 | extracellular |
| Glycoside Hydrolase | 78 | PITG_22085.1 | 4.39E-12 | | cytoplasm; nucleus |
| Glycoside Hydrolase | 81 | PITG_03508.1 | 1.07E-15 | | extracellular |
| Glycoside Hydrolase | 81 | PITG_03511.1 | 1.70E-32 | | extracellular |
| Glycoside Hydrolase | 81 | PITG_03512.1 | 3.66E-27 | 470, 50 | extracellular |
| Glycoside Hydrolase | 81 | PITG_03533.1 | 5.11E-29 | | extracellular |
| Glycoside Hydrolase | 81 | PITG_03535.1 | 8.98E-42 | 80, 50 | cytoplasm; mitochondria |
| Glycoside Hydrolase | 81 | PITG_03554.1 | 0.00E+00 | 50 | extracellular |
| Glycoside Hydrolase | 81 | PITG_03750.1 | 2.22E-32 | | cytoplasm |
| Glycoside Hydrolase | 81 | PITG_09760.1 | 4.13E-07 | 20, 280 | extracellular |
| Glycoside Hydrolase | 81 | PITG_10999.1 | 4.04E-34 | | extracellular |
| Glycoside Hydrolase | 81 | PITG_11050.1 | 3.09E-42 | | extracellular |
| Glycoside Hydrolase | 81 | PITG_11052.1 | 4.03E-42 | | extracellular |
| Glycoside Hydrolase | 81 | PITG_13322.1 | 7.60E-41 | | plasma membrane |
| Glycoside Hydrolase | 81 | PITG_13566.1 | 2.09E-06 | 30, 170, 540, 110 | cytoplasm |
| Glycoside Hydrolase | 81 | PITG_13567.1 | 1.64E-35 | | extracellular |
| Glycoside Hydrolase | 81 | PITG_13569.1 | 9.62E-36 | 570 | extracellular |
| Glycoside Hydrolase | 81 | PITG_13571.1 | 2.62E-33 | 175, 575 | extracellular |
| Glycoside Hydrolase | 81 | PITG_14173.1 | 1.10E-39 | 70, 70 | cytoplasm; mitochondria |
| Glycoside Hydrolase | 85 | PITG_06864.1 | 0.00E+00 | 60, 70, 70, 60 | cytoplasm |
| Glycoside Hydrolase | 89 | PITG_10027.1 | 0.00E+00 | 70 | cytoplasm |
| Glycoside Hydrolase | 89 | PITG_10050.1 | 0.00E+00 | 60, 70 | peroxisome |
| Glycoside Hydrolase | 92 | PITG_03465.1 | 2.82E-07 | | cytoplasm; mitochondria |
| Glycoside Hydrolase | 92 | PITG_05939.1 | 1.82E-14 | | cytoplasm; mitochondria |
| Glycoside Hydrolase | 92 | PITG_12819.1 | 3.56E-10 | 80 | cytoplasm |
| Glycoside Hydrolase | 92 | PITG_15640.1 | 3.01E-09 | | cytoplasm |
| Glycoside Hydrolase | 93 | PITG_04121.1 | 7.60E-11 | | cytoplasm |
| Glycoside Hydrolase | 93 | PITG_04983.1 | 7.86E-08 | 90, 120 | cytoplasm |
| Glycoside Hydrolase | 93 | PITG_04989.1 | 2.00E-11 | | cytoplasm |
| Glycoside Hydrolase | 93 | PITG_05373.1 | 3.64E-13 | | cytoplasm |
| Glycoside Hydrolase | 93 | PITG_08468.1 | 4.17E-09 | | cytoplasm |
| Glycoside Hydrolase | 93 | PITG_08842.1 | 1.10E-09 | | cytoplasm |
| Glycoside Hydrolase | 93 | PITG_08843.1 | 3.77E-10 | | cytoplasm |
| Glycoside Hydrolase | 93 | PITG_08845.1 | 1.10E-09 | 45 | cytoplasm |
| Glycoside Hydrolase | 93 | PITG_09003.1 | 4.17E-09 | | cytoplasm; mitochondria |
| Glycoside Hydrolase | 93 | PITG_17045.1 | 9.29E-09 | 320, 50 | cytoplasm |
| Glycoside Hydrolase | 93 | PITG_17049.1 | 2.53E-06 | 105, 590 | plasma membrane |

| | | | | | |
|---|---|---|---|---|---|
| Glycoside Hydrolase | 93 | PITG_19162.1 | 0.00E+00 | | cytoplasm |
| Glycoside Hydrolase | 95 | PITG_01391.1 | 5.48E-29 | | ND |
| Glycoside Hydrolase | 95 | PITG_01395.1 | 0.00E+00 | | ND |
| Glycoside Hydrolase | 95 | PITG_01396.1 | 0.00E+00 | | ND |
| Glycoside Hydrolase | 95 | PITG_01397.1 | 0.00E+00 | | ND |
| Glycoside Hydrolase | 95 | PITG_01398.1 | 0.00E+00 | | ND |
| Glycoside Hydrolase | 95 | PITG_01399.1 | 8.70E-43 | | ND |
| Glycoside Hydrolase | 95 | PITG_01430.1 | 0.00E+00 | | ND |
| Glycoside Hydrolase | 95 | PITG_01431.1 | 0.00E+00 | | ND |
| Glycoside Hydrolase | 95 | PITG_01462.1 | 0.00E+00 | | ND |
| Glycoside Hydrolase | 95 | PITG_01484.1 | 0.00E+00 | | ND |
| Glycoside Hydrolase | 95 | PITG_03460.1 | 4.33E-34 | | ND |
| Glycoside Hydrolase | 95 | PITG_05705.1 | 3.33E-18 | 90, 110, 310. 180, 190, 180, 180 | cytoplasm; mitochondria |
| Glycoside Hydrolase | 95 | PITG_09791.1 | 0.00E+00 | | ND |
| Glycoside Hydrolase | 95 | PITG_15790.1 | 0.00E+00 | | ND |
| Glycoside Hydrolase | 95 | PITG_15791.1 | 0.00E+00 | | cytoplasm |
| Glycoside Hydrolase | 95 | PITG_18208.1 | 0.00E+00 | | ND |
| Glycoside Hydrolase | 95 | PITG_18209.1 | 0.00E+00 | | ND |
| Glycoside Hydrolase | 95 | PITG_18230.1 | 0.00E+00 | | ND |
| Glycoside Hydrolase | 95 | PITG_18231.1 | 0.00E+00 | | cytoplasm |
| Glycoside Hydrolase | 95 | PITG_20005.1 | 6.71E-19 | | ND |
| Glycoside Hydrolase | 105 | PITG_11632.1 | 5.66E-36 | | extracellular |
| Glycoside Hydrolase | 105 | PITG_19782.1 | 5.12E-37 | | extracellular |
| Glycosyl Transferases | 1 | PITG_07104.1 | 8.09E-06 | 63 | not extracellular |
| Glycosyl Transferases | 1 | PITG_09296.1 | 6.83E-13 | 51, 110 | not extracellular |
| Glycosyl Transferases | 1 | PITG_09367.1 | 1.05E-13 | 600 | not extracellular |
| Glycosyl Transferases | 1 | PITG_09412.1 | 2.77E-14 | 220, 78 | not extracellular |
| Glycosyl Transferases | 1 | PITG_09413.1 | 3.51E-09 | 180 | not extracellular |
| Glycosyl Transferases | 1 | PITG_11723.1 | 2.28E-08 | 55, 150 61, 85, 85 | not extracellular |
| Glycosyl Transferases | 1 | PITG_18106.1 | 9.56E-07 | 180, 61, 73, 60, 61, 81, 450 | not extracellular |
| Glycosyl Transferases | 1 | PITG_21507.1 | 1.80E-13 | 168, 250 | not extracellular |
| Glycosyl Transferases | 1 | PITG_22471.1 | 3.29E-07 | 108 | not extracellular |
| Glycosyl Transferases | 2 | PITG_02050.1 | 0.00E+00 | | not extracellular |
| Glycosyl Transferases | 2 | PITG_09963.1 | 6.33E-07 | 240, 540 | not extracellular |
| Glycosyl Transferases | 2 | PITG_09964.1 | 3.36E-08 | | not extracellular |
| Glycosyl Transferases | 2 | PITG_16984.1 | 1.08E-06 | 77 | not extracellular |
| Glycosyl Transferases | 2 | PITG_17007.1 | 1.23E-10 | 104 | not extracellular |
| Glycosyl Transferases | 8 | PITG_06049.1 | 1.24E-06 | 140 | not extracellular |

| | | | | | |
|---|---|---|---|---|---|
| Glycosyl Transferases | 8 | PITG_18015.1 | 4.40E-12 | | not extracellular |
| Glycosyl Transferases | 19 | PITG_13917.1 | 3.97E-14 | 74, 60, 70, 77 | not extracellular |
| Glycosyl Transferases | 20 | PITG_01869.1 | 0.00E+00 | 340, 73, 63, 70, 80, 66, 52 | not extracellular |
| Glycosyl Transferases | 20 | PITG_02307.1 | 0.00E+00 | 175, 82 | not extracellular |
| Glycosyl Transferases | 20 | PITG_02575.1 | 0.00E+00 | 530, 70, 306, 200 | not extracellular |
| Glycosyl Transferases | 20 | PITG_06828.1 | 0.00E+00 | 64 | not extracellular |
| Glycosyl Transferases | 20 | PITG_09389.1 | 0.00E+00 | 90, 68 | not extracellular |
| Glycosyl Transferases | 20 | PITG_13721.1 | 0.00E+00 | 61, 55 | not extracellular |
| Glycosyl Transferases | 20 | PITG_15882.1 | 0.00E+00 | 62 | not extracellular |
| Glycosyl Transferases | 20 | PITG_20927.1 | 0.00E+00 | 67, 63, 60 80 | not extracellular |
| Glycosyl Transferases | 20 | PITG_21962.1 | 0.00E+00 | 69, 70 65, 85, 75, 76, 52 | not extracellular |
| Glycosyl Transferases | 22 | PITG_18105.1 | 1.11E-40 | Multiple | not extracellular |
| Glycosyl Transferases | 24 | PITG_07201.1 | 0.00E+00 | 67, 65, 46 | not extracellular |
| Glycosyl Transferases | 30 | PITG_21328.1 | 7.99E-17 | 110, 86, 77 60 | not extracellular |
| Glycosyl Transferases | 33 | PITG_11648.1 | 0.00E+00 | 68, 64, 66, 74, 67, 64, 55, 64, 72 | not extracellular |
| Glycosyl Transferases | 33 | PITG_22649.1 | 1.21E-22 | 68, 82 | not extracellular |
| Glycosyl Transferases | 39 | PITG_06984.1 | 7.20E-08 | 64 | not extracellular |
| Glycosyl Transferases | 41 | PITG_00983.1 | 3.09E-32 | 61, 52 | not extracellular |
| Glycosyl Transferases | 41 | PITG_01031.1 | 1.18E-15 | 68, 65 | not extracellular |
| Glycosyl Transferases | 41 | PITG_01416.1 | 4.50E-07 | 71 | not extracellular |
| Glycosyl Transferases | 41 | PITG_02952.1 | 8.46E-14 | 66 | not extracellular |
| Glycosyl Transferases | 41 | PITG_03348.1 | 1.89E-13 | | not extracellular |
| Glycosyl Transferases | 41 | PITG_03587.1 | 1.65E-09 | 61, 269, 79 | not extracellular |
| Glycosyl Transferases | 41 | PITG_04917.1 | 2.92E-06 | 75, 66, 46 | not extracellular |
| Glycosyl Transferases | 41 | PITG_06606.1 | 6.47E-22 | 100, 69, 63, 198 | not extracellular |
| Glycosyl Transferases | 41 | PITG_07702.1 | 1.03E-27 | 148 | not extracellular |
| Glycosyl Transferases | 41 | PITG_10094.1 | 9.07E-08 | 600, 250, 350 62, 62, 300, 60, 65, 62 | not extracellular |
| Glycosyl Transferases | 41 | PITG_10344.1 | 3.09E-32 | 61, 97, 61 | not extracellular |
| Glycosyl Transferases | 41 | PITG_12110.1 | 4.07E-08 | 62, 69 | not extracellular |
| Glycosyl Transferases | 41 | PITG_17013.1 | 1.07E-08 | 91, 68, 123, 76, 81 | not extracellular |
| Glycosyl Transferases | 41 | PITG_17928.1 | 0.00E+00 | 126, 79 | not extracellular |
| Glycosyl Transferases | 48 | PITG_03335.1 | 0.00E+00 | 52 | not extracellular |
| Glycosyl Transferases | 48 | PITG_03473.1 | 0.00E+00 | | not extracellular |
| Glycosyl Transferases | 48 | PITG_05079.1 | 0.00E+00 | | not extracellular |
| Glycosyl Transferases | 48 | PITG_06358.1 | 0.00E+00 | | not extracellular |
| Glycosyl Transferases | 48 | PITG_06428.1 | 0.00E+00 | | not extracellular |

| | | | | | |
|---|---|---|---|---|---|
| Glycosyl Transferases | 48 | PITG_14727.1 | 0.00E+00 | | not extracellular |
| Glycosyl Transferases | 50 | PITG_17126.1 | 5.77E-30 | 78, 186, 60, 79, 85, 165 | not extracellular |
| Glycosyl Transferases | 55 | PITG_05763.1 | 5.31E-20 | 143 | not extracellular |
| Glycosyl Transferases | 55 | PITG_06491.1 | 6.07E-16 | 56, 57, 62, 58 | not extracellular |
| Glycosyl Transferases | 55 | PITG_10728.1 | 3.01E-23 | 380, 320 | not extracellular |
| Glycosyl Transferases | 55 | PITG_11363.1 | 2.63E-19 | | not extracellular |
| Glycosyl Transferases | 57 | PITG_05541.1 | 4.57E-31 | | not extracellular |
| Glycosyl Transferases | 57 | PITG_13291.1 | 8.60E-38 | | not extracellular |
| Glycosyl Transferases | 58 | PITG_02432.1 | 4.49E-33 | 63 | not extracellular |
| Glycosyl Transferases | 59 | PITG_03060.1 | 8.48E-36 | 67, 62, 71, 182, 96, 67, 34 | not extracellular |
| Glycosyl Transferases | 59 | PITG_21941.1 | 4.37E-16 | 67, 62, 71, 95 | not extracellular |
| Glycosyl Transferases | 61 | PITG_14473.1 | 8.75E-06 | 43, 53 | not extracellular |
| Glycosyl Transferases | 61 | PITG_14483.1 | 4.20E-08 | | not extracellular |
| Glycosyl Transferases | 62 | PITG_11398.1 | 1.16E-07 | 354 | not extracellular |
| Glycosyl Transferases | 66 | PITG_00632.1 | 0.00E+00 | 71, 108 | not extracellular |
| Glycosyl Transferases | 66 | PITG_04610.1 | 0.00E+00 | 62, 87, 69 | not extracellular |
| Glycosyl Transferases | 71 | PITG_02291.1 | 3.44E-06 | | not extracellular |
| Glycosyl Transferases | 71 | PITG_02294.1 | 4.35E-09 | 580, 91 | not extracellular |
| Glycosyl Transferases | 71 | PITG_17661.1 | 2.64E-06 | 61 | not extracellular |
| Glycosyl Transferases | 71 | PITG_17663.1 | 2.64E-06 | | not extracellular |
| Glycosyl Transferases | 71 | PITG_17664.1 | 8.76E-10 | 130 | not extracellular |
| Glycosyl Transferases | 71 | PITG_18255.1 | 4.35E-09 | | not extracellular |
| Glycosyl Transferases | 71 | PITG_18258.1 | 6.71E-10 | | not extracellular |
| Glycosyl Transferases | 71 | PITG_18266.1 | 1.55E-06 | 73, 58 | not extracellular |
| Glycosyl Transferases | 71 | PITG_18270.1 | 1.07E-07 | 106, 80 | not extracellular |
| Glycosyl Transferases | 71 | PITG_18271.1 | 4.64E-11 | | not extracellular |
| Glycosyl Transferases | 71 | PITG_18272.1 | 3.68E-08 | 160 | not extracellular |
| Glycosyl Transferases | 71 | PITG_18275.1 | 6.71E-10 | | not extracellular |
| Glycosyl Transferases | 71 | PITG_18276.1 | 4.35E-09 | 76 | not extracellular |
| Glycosyl Transferases | 71 | PITG_18277.1 | 7.67E-06 | 118 | not extracellular |
| Glycosyl Transferases | 71 | PITG_18279.1 | 2.64E-06 | 136, 161 | not extracellular |
| Glycosyl Transferases | 76 | PITG_13172.1 | 6.84E-27 | 66 | not extracellular |
| Polysaccharide Lyase | 1 | PITG_00872.1 | 0.00E+00 | | extracellular |
| Polysaccharide Lyase | 1 | PITG_05312.1 | 2.50075E-13 | 167, 281, 39, 43 | cytoplasm; nucleus |
| Polysaccharide Lyase | 1 | PITG_07680.1 | 6.3152E-41 | | extracellular |
| Polysaccharide Lyase | 1 | PITG_08555.1 | 0.00E+00 | 72 | extracellular |
| Polysaccharide Lyase | 1 | PITG_08558.1 | 0.00E+00 | 46, 98, 51, 43 | extracellular |
| Polysaccharide Lyase | 1 | PITG_08561.1 | 1.4E-45 | 71 | extracellular |

| | | | | | |
|---|---|---|---|---|---|
| Polysaccharide Lyase | 1 | PITG_08563.1 | 8.248E-41 | 167, 111 | extracellular |
| Polysaccharide Lyase | 1 | PITG_08564.1 | 2.03156E-39 | 138 | extracellular |
| Polysaccharide Lyase | 1 | PITG_09457.1 | 0.00E+00 | 53 | extracellular |
| Polysaccharide Lyase | 1 | PITG_09465.1 | 0.00E+00 | 46, 91 | extracellular |
| Polysaccharide Lyase | 1 | PITG_09466.1 | 1.41344E-24 | | cytoplasm |
| Polysaccharide Lyase | 1 | PITG_09467.1 | 1.30613E-21 | | extracellular |
| Polysaccharide Lyase | 1 | PITG_19909.1 | 1.79216E-11 | 144 | cytoplasm; mitochondria |
| Polysaccharide Lyase | 1 | PITG_20992.1 | 6.15956E-12 | 149 | cytoplasm; mitochondria |
| Polysaccharide Lyase | 1 | PITG_21462.1 | 0.00E+00 | 47, 91 | extracellular |
| Polysaccharide Lyase | 1 | PITG_21463.1 | 0.00E+00 | | extracellular |
| Polysaccharide Lyase | 1 | PITG_21498.1 | 1.85459E-08 | | extracellular |
| Polysaccharide Lyase | 1 | PITG_21499.1 | 2.56993E-34 | | extracellular |
| Polysaccharide Lyase | 3 | PITG_04668.1 | 4.55351E-26 | 98 | extracellular |
| Polysaccharide Lyase | 3 | PITG_08632.1 | 1.15527E-37 | | cytoplasm |
| Polysaccharide Lyase | 3 | PITG_08633.1 | 2.30022E-25 | 98 | extracellular |
| Polysaccharide Lyase | 3 | PITG_08634.1 | 1.19552E-34 | 41 | cytoplasm; nucleus |
| Polysaccharide Lyase | 3 | PITG_08635.1 | 2.57965E-29 | 78 | cytoplasm; mitochondrial matrix space |
| Polysaccharide Lyase | 3 | PITG_08638.1 | 1.50883E-37 | 84 | cytoplasm; nucleus |
| Polysaccharide Lyase | 3 | PITG_08646.1 | 7.23617E-40 | 57 | cytoplasm; nucleus |
| Polysaccharide Lyase | 3 | PITG_08647.1 | 1.78233E-38 | | cytoplasm; nucleus |
| Polysaccharide Lyase | 3 | PITG_09254.1 | 0.00E+00 | | extracellular |
| Polysaccharide Lyase | 3 | PITG_09255.1 | 9.47266E-32 | 64, 60 | cytoplasm; nucleus |
| Polysaccharide Lyase | 3 | PITG_09256.1 | 0.00E+00 | | cytoplasm |
| Polysaccharide Lyase | 3 | PITG_09257.1 | 0.00E+00 | 47 | extracellular |
| Polysaccharide Lyase | 3 | PITG_09258.1 | 1.51233E-29 | 84 | cytoplasm; nucleus |
| Polysaccharide Lyase | 3 | PITG_09259.1 | 0.00E+00 | 44 | cytoplasm; mitochondria |
| Polysaccharide Lyase | 3 | PITG_09821.1 | 3.92058E-21 | 98 | cytoplasm; mitochondrial matrix space |
| Polysaccharide Lyase | 3 | PITG_10424.1 | 7E-45 | 572 | extracellular |
| Polysaccharide Lyase | 3 | PITG_12828.1 | 0.00E+00 | | extracellular |
| Polysaccharide Lyase | 3 | PITG_12829.1 | 0.00E+00 | | extracellular |
| Polysaccharide Lyase | 3 | PITG_14167.1 | 0.00E+00 | | plasma membrane |
| Polysaccharide Lyase | 3 | PITG_14168.1 | 1.4E-45 | | extracellular |
| Polysaccharide Lyase | 3 | PITG_14287.1 | 0.00E+00 | 166 | extracellular |
| Polysaccharide Lyase | 3 | PITG_14288.1 | 1.4E-45 | 83, 166 | cytoplasm; mitochondria |
| Polysaccharide Lyase | 3 | PITG_15296.1 | 9.14E-43 | 580, 403, 338, 639, 572, 134 | extracellular |
| Polysaccharide Lyase | 3 | PITG_15299.1 | 2.66334E-34 | 48 | extracellular |
| Polysaccharide Lyase | 3 | PITG_16160.1 | 0.00E+00 | 166 | extracellular |
| Polysaccharide Lyase | 3 | PITG_17207.1 | 0.00E+00 | 201 | extracellular |
| Polysaccharide Lyase | 3 | PITG_17208.1 | 1.97059E-37 | | cytoplasm; nucleus |

| | | | | | |
|---|---|---|---|---|---|
| Polysaccharide Lyase | 3 | PITG_17210.1 | 2.11029E-31 | 44 | cytoplasm; microbody; nucleus |
| Polysaccharide Lyase | 3 | PITG_18477.1 | 5.74686E-29 | 44 | cytoplasm; nucleus |
| Polysaccharide Lyase | 3 | PITG_18478.1 | 1.3188E-41 | 158, 42 | cytoplasm; microbody; nucleus |
| Polysaccharide Lyase | 3 | PITG_18482.1 | 0.00E+00 | 201 | extracellular |
| Polysaccharide Lyase | 3 | PITG_19366.1 | 0.00E+00 | 211 | extracellular |
| Polysaccharide Lyase | 3 | PITG_21713.1 | 3.04725E-30 | | extracellular |
| Polysaccharide Lyase | 3 | PITG_21714.1 | 0.00E+00 | 79 | cytoplasm; mitochondrial matrix space |
| Polysaccharide Lyase | 3 | PITG_22166.1 | 8.56766E-33 | 95 | cytoplasm; microbody; nucleus |
| Polysaccharide Lyase | 3 | PITG_22227.1 | 0.00E+00 | | cytoplasm; nucleus |
| Polysaccharide Lyase | 3 | PITG_22228.1 | 8.86614E-30 | | extracellular |
| Polysaccharide Lyase | 3 | PITG_22632.1 | 1.2856E-22 | 41, 46 | cytoplasm; mitochondrial matrix space |
| Polysaccharide Lyase | 4 | PITG_02331.1 | 3.42646E-33 | 72 | cytoplasm; microbody |
| Polysaccharide Lyase | 4 | PITG_02332.1 | 0.00E+00 | 42 | extracellular |
| Polysaccharide Lyase | 4 | PITG_02368.1 | 0.00E+00 | 42, 52, 69 | extracellular |
