# Supplementary material for: The CAZyome of Phytophthora spp.: A comprehensive analysis of the gene complement coding for carbohydrate-active enzymes in species of the genus Phytophthora
Source: BMC Genomics. 2010 Sep 28;11:525. doi: 10.1186/1471-2164-11-525 (PMC2997016; doi:10.1186/1471-2164-11-525)
Supplement: Additional file 2 — CAZy genes in P. sojae. CAZyme-coding homologs in P. sojae organized by their respective CAZy superfamily. 1Numbers represent intron sizes; "multiple" refers to the fact that more than 9 introns were present in the gene model. 2Most likely cellular localization predicted by SecretomeP, SignalP, or TargetP algorithms. ND, not determined. [file 1471-2164-11-525-S2.PDF]

CAZy Group	Family	Gene Identifier	E Value	Introns[1]	Predicted Cellular Localization[2]
Carbohydrate Esterases	2	142493	1.00E-08	Multiple	cytoplasm; nucleus
Carbohydrate Esterases	5	109740	3.00E-07	46	extracellular
Carbohydrate Esterases	5	109741	5.00E-07		extracellular
Carbohydrate Esterases	5	127974	2.00E-06		extracellular
Carbohydrate Esterases	5	127975	2.00E-06		extracellular
Carbohydrate Esterases	5	127976	2.00E-06		extracellular
Carbohydrate Esterases	5	127977	2.00E-06		extracellular
Carbohydrate Esterases	5	127978	4.00E-07		extracellular
Carbohydrate Esterases	5	127979	8.00E-07		extracellular
Carbohydrate Esterases	5	127980	8.00E-07		extracellular
Carbohydrate Esterases	5	127981	5.00E-06		extracellular
Carbohydrate Esterases	5	127982	3.00E-07		extracellular
Carbohydrate Esterases	5	127983	5.00E-07		extracellular
Carbohydrate Esterases	5	143279	1.00E-06		extracellular
Carbohydrate Esterases	5	143280	1.00E-06		extracellular
Carbohydrate Esterases	8	108139	1.00E-37		extracellular
Carbohydrate Esterases	8	108149	1.00E-13		cytoplasm; nucleus
Carbohydrate Esterases	8	117650	1.00E-31	37	extracellular
Carbohydrate Esterases	8	127156	1.00E-09		extracellular
Carbohydrate Esterases	8	127157	1.00E-15		extracellular
Carbohydrate Esterases	8	127162	9.00E-07		cytoplasm; mitochondria
Carbohydrate Esterases	8	128733	1.00E-07		extracellular
Carbohydrate Esterases	8	128784	3.00E-08		extracellular
Carbohydrate Esterases	8	131634	6.00E-14	307, 238	cytoplasm; mitochondria
Carbohydrate Esterases	8	131657	5.00E-06	44	extracellular
Carbohydrate Esterases	8	133883	8.00E-07	63	extracellular
Carbohydrate Esterases	8	134353	4.00E-09		extracellular
Carbohydrate Esterases	8	134354	7.00E-10		extracellular
Carbohydrate Esterases	8	134355	6.00E-11	25, 149	extracellular
Carbohydrate Esterases	8	143742	3.00E-07		extracellular
Carbohydrate Esterases	8	143743	1.00E-13		extracellular
Carbohydrate Esterases	8	144374	4.00E-06		extracellular
Carbohydrate Esterases	8	144376	1.00E-37		extracellular
Carbohydrate Esterases	8	144377	5.00E-20	110	extracellular
Carbohydrate Esterases	8	144380	2.00E-21		cytoplasm; nucleus
Glycoside Hydrolase	1	108504	1.00E-91		cytoplasm
Glycoside Hydrolase	1	108505	5.00E-82		cytoplasm

Glycoside Hydrolase	1	108507	4.00E-84		cytoplasm
Glycoside Hydrolase	1	135936	2.00E-34		extracellular
Glycoside Hydrolase	1	136756	7.00E-33	391, 79	cytoplasm
Glycoside Hydrolase	1	136757	8.00E-24	26, 79	extracellular
Glycoside Hydrolase	1	136981	6.00E-43	198, 243	cytoplasm; mitochondria
Glycoside Hydrolase	1	137835	6.00E-43		extracellular
Glycoside Hydrolase	1	137885	9.00E-40		extracellular
Glycoside Hydrolase	1	137886	1.00E-36		extracellular
Glycoside Hydrolase	1	137928	2.00E-19	303, 278, 73, 78, 109	cytoplasm; nucleus
Glycoside Hydrolase	1	137929	5.00E-43		extracellular
Glycoside Hydrolase	1	137930	2.00E-36		extracellular
Glycoside Hydrolase	1	137931	2.00E-29		cytoplasm; mitochondria
Glycoside Hydrolase	1	137933	1.00E-44		extracellular
Glycoside Hydrolase	1	143481	2.00E-25	183	plama membrane
Glycoside Hydrolase	1	143830	9.00E-13	31, 35	extracellular
Glycoside Hydrolase	1	144887	1.00E-29	31, 161, 170, 155, 102	cytoplasm; mitochondria
Glycoside Hydrolase	1	144981	3.00E-12	127	extracellular
Glycoside Hydrolase	3	131843	2.00E-09	52, 85	extracellular
Glycoside Hydrolase	3	131848	2.00E-09	52, 85	extracellular
Glycoside Hydrolase	3	131903	3.00E-08		extracellular
Glycoside Hydrolase	3	133642	6.00E-06		extracellular
Glycoside Hydrolase	3	133710	5.00E-07		extracellular
Glycoside Hydrolase	3	133713	6.00E-06		extracellular*
Glycoside Hydrolase	3	134112	3.00E-56	125, 20	extracellular
Glycoside Hydrolase	3	135218	3.00E-10		extracellular
Glycoside Hydrolase	3	137272	2.00E-10	81	extracellular
Glycoside Hydrolase	3	138254	6.00E-87		extracellular
Glycoside Hydrolase	3	144049	7.00E-59		extracellular
Glycoside Hydrolase	3	144555	1.00E-65	89	extracellular*
Glycoside Hydrolase	3	144556	3.00E-07	87	cytoplasm; nucleus
Glycoside Hydrolase	3	144643	4.00E-36	31818	extracellular
Glycoside Hydrolase	3	145004	3.00E-09	52	extracellular
Glycoside Hydrolase	3	145259	2.00E-06	4998, 145, 69	extracellular
Glycoside Hydrolase	5	129514	8.00E-96	19, 191, 247	cytoskeleton
Glycoside Hydrolase	5	129708	1.00E-11	164, 63	extracellular
Glycoside Hydrolase	5	131561	4.00E-16		cytoplasm
Glycoside Hydrolase	5	132075	1.00E-37		plama membrane
Glycoside Hydrolase	5	132177	1.00E-153	77	plama membrane

Glycoside Hydrolase	5	132179	1.00E-136	76	cytoplasm; nucleus
Glycoside Hydrolase	5	132180	1.00E-162	83, 81, 72, 77	cytoplasm; mitochondria
Glycoside Hydrolase	5	135931	1.00E-159	259, 194, 204,80, 77	cytoplasm; mitochondria
Glycoside Hydrolase	5	135932	0.00E+00	96, 93	plama membrane
Glycoside Hydrolase	5	136107	1.00E-62	70	cytoplasm; mitochondria
Glycoside Hydrolase	5	144753	5.00E-17	68	extracellular*
Glycoside Hydrolase	5	145627	5.00E-47	41	extracellular
Glycoside Hydrolase	6	131166	4.00E-07		extracellular
Glycoside Hydrolase	6	132586	5.00E-11		extracellular
Glycoside Hydrolase	6	132587	3.00E-10		extracellular
Glycoside Hydrolase	6	132588	6.00E-08	125	cytoplasm; nucleus
Glycoside Hydrolase	6	132597	1.00E-10	726	extracellular
Glycoside Hydrolase	6	132598	4.00E-07		extracellular*
Glycoside Hydrolase	6	140501	1.00E-10		extracellular
Glycoside Hydrolase	6	144321	2.00E-10	98	extracellular
Glycoside Hydrolase	7	108489	7.00E-32		cytoplasm
Glycoside Hydrolase	7	108490	1.00E-44	257	cytoplasm; mitochondria
Glycoside Hydrolase	7	108678	1.00E-51	31	cytoskeleton
Glycoside Hydrolase	7	108891	3.00E-42	48	extracellular
Glycoside Hydrolase	7	109096	3.00E-59	24	extracellular
Glycoside Hydrolase	7	135666	3.00E-43	73, 31,119	cytoplasm; nucleus
Glycoside Hydrolase	7	135667	8.00E-49	88, 49	extracellular
Glycoside Hydrolase	10	108296	2.00E-27	71	extracellular
Glycoside Hydrolase	10	131015	2.00E-22	77, 102	extracellular
Glycoside Hydrolase	10	131028	5.00E-11	41, 19	extracellular
Glycoside Hydrolase	10	131360	1.00E-06		extracellular
Glycoside Hydrolase	10	140368	3.00E-27	75, 81	extracellular
Glycoside Hydrolase	10	140369	7.00E-27	79, 74	extracellular
Glycoside Hydrolase	12	109280	2.00E-17		extracellular
Glycoside Hydrolase	12	109281	2.00E-20		extracellular
Glycoside Hydrolase	12	109681	2.00E-42		extracellular
Glycoside Hydrolase	12	109713	2.00E-14		cytoplasm
Glycoside Hydrolase	12	119627	2.00E-36		extracellular
Glycoside Hydrolase	12	126103	5.00E-11		extracellular
Glycoside Hydrolase	12	138787	5.00E-21		extracellular
Glycoside Hydrolase	12	140289	3.00E-20	73, 145	extracellular
Glycoside Hydrolase	12	140297	1.00E-14	168, 37, 23	cytoplasm
Glycoside Hydrolase	12	140300	2.00E-14		extracellular

Glycoside Hydrolase	12	140301	4.00E-21		extracellular
Glycoside Hydrolase	12	140302	4.00E-21		extracellular
Glycoside Hydrolase	12	140303	3.00E-14		extracellular
Glycoside Hydrolase	12	144813	2.00E-31	44	extracellular
Glycoside Hydrolase	17	108444	2.00E-55		extracellular*
Glycoside Hydrolase	17	128780	1.00E-64		extracellular
Glycoside Hydrolase	17	128781	1.00E-64		extracellular
Glycoside Hydrolase	17	128802	5.00E-50	23	cytoplasm
Glycoside Hydrolase	17	138913	7.00E-33		extracellular
Glycoside Hydrolase	17	138916	1.00E-28	48	extracellular
Glycoside Hydrolase	17	141907	1.00E-68	70, 36, 139	extracellular
Glycoside Hydrolase	17	141908	1.00E-139	82	cytoplasm
Glycoside Hydrolase	17	141909	4.00E-68	63, 64	extracellular
Glycoside Hydrolase	17	144000	2.00E-37	53	extracellular
Glycoside Hydrolase	19	142416	8.00E-23		extracellular
Glycoside Hydrolase	28	108848	3.00E-25		extracellular
Glycoside Hydrolase	28	108849	7.00E-32		extracellular
Glycoside Hydrolase	28	108850	7.00E-21		extracellular
Glycoside Hydrolase	28	108851	1.00E-19	37	extracellular
Glycoside Hydrolase	28	108985	1.00E-21	26	extracellular
Glycoside Hydrolase	28	109521	2.00E-12	85	extracellular
Glycoside Hydrolase	28	109537	3.00E-18	48, 68, 97	extracellular
Glycoside Hydrolase	28	109634	8.00E-16	26	extracellular
Glycoside Hydrolase	28	109695	4.00E-20	53, 115, 34, 21	extracellular
Glycoside Hydrolase	28	116769	7.00E-21	243, 17	extracellular
Glycoside Hydrolase	28	116776	5.00E-21	81, 285	extracellular
Glycoside Hydrolase	28	127373	8.00E-09		extracellular
Glycoside Hydrolase	28	135139	2.00E-13	94	extracellular
Glycoside Hydrolase	28	135140	5.00E-17	36, 82, 23	extracellular
Glycoside Hydrolase	28	135142	1.00E-14	65, 2500	cytoplasm; nucleus
Glycoside Hydrolase	28	135143	5.00E-19	56	cytoplasm
Glycoside Hydrolase	28	135145	2.00E-22	152, 50	extracellular
Glycoside Hydrolase	28	135147	7.00E-32		extracellular
Glycoside Hydrolase	28	135149	7.00E-32		extracellular
Glycoside Hydrolase	28	135150	9.00E-18	32, 20, 29, 556, 91	extracellular
Glycoside Hydrolase	28	135151	9.00E-18		extracellular
Glycoside Hydrolase	28	135156	2.00E-20	41, 115, 46	extracellular
Glycoside Hydrolase	28	144067	2.00E-16	678, 20, 28, 289	extracellular

Glycoside Hydrolase	28	145625	6.00E-21		extracellular
Glycoside Hydrolase	30	108522	1.00E-09		extracellular
Glycoside Hydrolase	30	130485	4.00E-07	39, 34	cytoplasm
Glycoside Hydrolase	30	130491	3.00E-09	35	extracellular
Glycoside Hydrolase	30	130492	5.00E-09		extracellular
Glycoside Hydrolase	30	130493	2.00E-11		extracellular
Glycoside Hydrolase	30	130494	3.00E-08		extracellular
Glycoside Hydrolase	30	130496	3.00E-06	87	extracellular
Glycoside Hydrolase	30	130499	3.00E-10		extracellular
Glycoside Hydrolase	30	135397	2.00E-06	152, 44	cytoplasm; nucleus; microbody
Glycoside Hydrolase	30	138213	4.00E-10		plama membrane
Glycoside Hydrolase	30	138214	4.00E-06	43, 121	cytoplasm; mitochondrial matrix space
Glycoside Hydrolase	30	138217	1.00E-07		cytoplasm; mitochondria
Glycoside Hydrolase	31	108542	6.00E-17	33, 72, 57	extracellular
Glycoside Hydrolase	31	130344	2.00E-18		extracellular
Glycoside Hydrolase	31	130345	1.00E-13		extracellular
Glycoside Hydrolase	31	141207	1.00E-126	136, 75, 45, 52	extracellular
Glycoside Hydrolase	31	142617	9.00E-07	92, 426, 78, 294, 24, 261, 148, 98	plama membrane
Glycoside Hydrolase	31	143202	1.00E-128	268, 62	extracellular
Glycoside Hydrolase	32	137802	9.00E-19		extracellular
Glycoside Hydrolase	32	137803	5.00E-19		extracellular
Glycoside Hydrolase	32	137804	2.00E-15	42, 44, 35	extracellular
Glycoside Hydrolase	35	155253	7.00E-16	42, 24, 148, 93, 72, 383, 21, 147, 60	cytoplasm; mitochondria
Glycoside Hydrolase	37	108895	5.00E-21		peroxisome
Glycoside Hydrolase	37	135687	1.00E-19		extracellular
Glycoside Hydrolase	47	108279	1.00E-25	45, 108	cytoplasm
Glycoside Hydrolase	47	117121	5.00E-45	74, 81, 75	cytoplasm
Glycoside Hydrolase	47	118482	7.00E-56	65, 62, 58	cytoplasm
Glycoside Hydrolase	47	123276	4.00E-37	80, 86, 84, 70, 82	cytoplasm; mitochondria
Glycoside Hydrolase	47	141218	4.00E-17	57, 159	extracellular
Glycoside Hydrolase	53	131878	5.00E-47	68	extracellular
Glycoside Hydrolase	53	131879	3.00E-46	53	extracellular
Glycoside Hydrolase	53	131880	5.00E-33	68, 38, 32	extracellular
Glycoside Hydrolase	53	131883	2.00E-54	69	extracellular
Glycoside Hydrolase	54	108620	1.00E-99	19	extracellular
Glycoside Hydrolase	54	132315	1.00E-106		extracellular
Glycoside Hydrolase	63	136691	2.00E-81		cytoplasm; mitochondria
Glycoside Hydrolase	72	128950	3.00E-12	480, 20, 35	extracellular*

Glycoside Hydrolase	72	128951	1.00E-09		extracellular*
Glycoside Hydrolase	72	128952	2.00E-11	78, 78	extracellular*
Glycoside Hydrolase	72	128955	3.00E-08	70, 95	extracellular
Glycoside Hydrolase	72	130504	2.00E-07		extracellular*
Glycoside Hydrolase	72	130505	1.00E-07	53	cytoplasm
Glycoside Hydrolase	72	138391	6.00E-07	50, 92, 68, 69	extracellular*
Glycoside Hydrolase	72	138398	4.00E-11	87, 81, 61, 74, 126	extracellular*
Glycoside Hydrolase	72	138399	4.00E-11	419, 87, 81, 61, 74, 69	plama membrane*
Glycoside Hydrolase	72	142342	2.00E-09	103, 65, 74, 80	extracellular*
Glycoside Hydrolase	72	157826	9.00E-11	Multiple	plama membrane
Glycoside Hydrolase	72	157827	1.00E-10	66, 65, 64, 76, 80	extracellular*
Glycoside Hydrolase	81	133043	2.00E-50	111	cytoplasm
Glycoside Hydrolase	81	134766	2.00E-51	66, 43	cytoplasm
Glycoside Hydrolase	81	134769	1.00E-22	321, 103	cytoplasm; mitochondria
Glycoside Hydrolase	81	134784	2.00E-61		extracellular
Glycoside Hydrolase	81	134785	3.00E-59		extracellular
Glycoside Hydrolase	81	136482	8.00E-37		cytoplasm
Glycoside Hydrolase	81	136653	2.00E-64		extracellular
Glycoside Hydrolase	81	136654	2.00E-64		extracellular
Glycoside Hydrolase	81	137093	2.00E-58		cytoplasm
Glycoside Hydrolase	81	138216	9.00E-31	76, 128	cytoplasm; mitochondria
Glycoside Hydrolase	81	140360	2.00E-33	75	extracellular
Glycoside Hydrolase	81	142277	2.00E-60		cytoplasm; mitochondria
Glycoside Hydrolase	81	142344	1.00E-38	94, 465, 47, 153, 45, 50, 54	cytoplasm; mitochondria
Glycoside Hydrolase	81	143889	7.00E-31	375, 465, 47, 153, 45, 96, 41, 91, 20	cytoplasm
Glycoside Hydrolase	81	144045	1.00E-24		cytoplasm
Glycoside Hydrolase	81	144699	4.00E-50	20, 35, 41	extracellular
Glycoside Hydrolase	81	144768	2.00E-51	66, 89	cytoplasm; nucleus
Glycoside Hydrolase	81	144920	2.00E-59	169	peroxisome
Glycoside Hydrolase	81	144926	2.00E-41	713, 33, 804, 95, 54	cytoplasm
Glycoside Hydrolase	81	145612	4.00E-58	319	extracellular
Glycoside Hydrolase	85	135747	4.00E-50	207, 83, 381, 57 29	plama membrane
Glycoside Hydrolase	89	131764	1.00E-139	79, 259, 75, 66	extracellular
Glycoside Hydrolase	89	131783	1.00E-137	84, 67	extracellular
Glycoside Hydrolase	95	108754	7.00E-30		cytoplasm
Glycoside Hydrolase	95	144153	4.00E-46		cytoplasm; mitochondria
Glycoside Hydrolase	105	127158	9.00E-35	300, 92	extracellular
Glycosyl Transferases	8	140071	1.00E-08		not extracellular

Glycosyl Transferases	20	120536	1.00E-77		not extracellular
Glycosyl Transferases	20	128418	8.00E-69		not extracellular
Glycosyl Transferases	20	130468	7.00E-70	58, 183, 23	not extracellular
Glycosyl Transferases	20	135706	5.00E-50		not extracellular
Glycosyl Transferases	20	137445	6.00E-41	75, 223, 156, 88, 66, 68, 63	not extracellular
Glycosyl Transferases	20	141232	9.00E-35	75	not extracellular
Glycosyl Transferases	20	142040	2.00E-46	65, 61, 202	not extracellular
Glycosyl Transferases	20	144346	3.00E-72	Multiple	not extracellular
Glycosyl Transferases	22	135928	5.00E-71		not extracellular
Glycosyl Transferases	22	137236	4.00E-22	73, 189, 68, 78, 75, 71, 214, 79, 69	not extracellular
Glycosyl Transferases	24	124281	9.00E-21		not extracellular
Glycosyl Transferases	1	137754	9.00E-25	Multiple	not extracellular
Glycosyl Transferases	1	137758	6.00E-47		not extracellular
Glycosyl Transferases	1	141158	2.00E-50	70, 193	not extracellular
Glycosyl Transferases	1	141208	9.00E-06	91, 243, 21	not extracellular
Glycosyl Transferases	1	141209	9.00E-39	28, 209	not extracellular
Glycosyl Transferases	1	141210	4.00E-30	28, 95, 602, 91, 243	not extracellular
Glycosyl Transferases	1	142345	2.00E-44	Multiple	not extracellular
Glycosyl Transferases	1	142346	1.00E-51	62	not extracellular
Glycosyl Transferases	1	145323	4.00E-24	28	not extracellular
Glycosyl Transferases	1	156256	1.00E-10	Multiple	not extracellular
Glycosyl Transferases	1	156310	8.00E-07	Multiple	not extracellular
Glycosyl Transferases	1	157557	9.00E-09	Multiple	not extracellular
Glycosyl Transferases	1	158733	7.00E-18	30, 89, 71	not extracellular
Glycosyl Transferases	2	108186	7.00E-17	39, 31	not extracellular
Glycosyl Transferases	2	127507	5.00E-11		not extracellular
Glycosyl Transferases	2	128905	6.00E-06	145, 68	not extracellular
Glycosyl Transferases	2	129945	6.00E-13	78, 54, 77	not extracellular
Glycosyl Transferases	2	130188	2.00E-10	88	not extracellular
Glycosyl Transferases	2	131831	3.00E-12		not extracellular
Glycosyl Transferases	2	131832	0.00E+00	141	not extracellular
Glycosyl Transferases	2	134349	2.00E-15		not extracellular
Glycosyl Transferases	2	136540	1.00E-21		not extracellular
Glycosyl Transferases	2	137085	2.00E-10	71	not extracellular
Glycosyl Transferases	2	137843	2.00E-14		not extracellular
Glycosyl Transferases	2	138206	5.00E-20	73, 121, 102	not extracellular
Glycosyl Transferases	2	140288	7.00E-10	94	not extracellular
Glycosyl Transferases	2	140880	1.00E-11	59	not extracellular

Glycosyl Transferases	2	141887	2.00E-20	101	not extracellular
Glycosyl Transferases	2	142577	1.00E-23		not extracellular
Glycosyl Transferases	2	142762	2.00E-17	98, 129	not extracellular
Glycosyl Transferases	2	143614	7.00E-86		not extracellular
Glycosyl Transferases	2	143645	5.00E-13		not extracellular
Glycosyl Transferases	2	155246	3.00E-73	77, 201, 88, 73, 70, 76, 65, 68, 142	not extracellular
Glycosyl Transferases	2	155337	9.00E-08	84, 42, 54, 171, 27, 56	not extracellular
Glycosyl Transferases	2	155779	7.00E-11	27, 51, 60, 70, 56, 183, 111	not extracellular
Glycosyl Transferases	2	156906	5.00E-25	54, 183, 30, 172, 251, 81, 65	not extracellular
Glycosyl Transferases	2	157622	4.00E-07	86, 51, 69, 33, 36, 321, 99, 36	not extracellular
Glycosyl Transferases	2	158132	1.00E-158	Multiple	not extracellular
Glycosyl Transferases	4	108303	2.00E-09		not extracellular
Glycosyl Transferases	4	108450	4.00E-55		not extracellular
Glycosyl Transferases	32	137358	8.00E-06		not extracellular
Glycosyl Transferases	32	141323	6.00E-08		not extracellular
Glycosyl Transferases	32	158858	3.00E-06	Multiple	not extracellular
Glycosyl Transferases	33	113542	1.00E-77	88, 77, 119, 85, 74	not extracellular
Glycosyl Transferases	41	138310	5.00E-30	56, 64	not extracellular
Glycosyl Transferases	41	140093	5.00E-09		not extracellular
Glycosyl Transferases	41	143341	1.00E-115	103	not extracellular
Glycosyl Transferases	41	155230	8.00E-25	36, 39, 171, 69, 51, 54	not extracellular
Glycosyl Transferases	48	130870	1.00E-06		not extracellular
Glycosyl Transferases	48	134121	5.00E-87	85	not extracellular
Glycosyl Transferases	48	134122	4.00E-90	64, 134	not extracellular
Glycosyl Transferases	48	134234	6.00E-27		not extracellular
Glycosyl Transferases	48	134235	2.00E-90		not extracellular
Glycosyl Transferases	48	134329	2.00E-71		not extracellular
Glycosyl Transferases	48	139198	1.00E-79		not extracellular
Glycosyl Transferases	48	141900	1.00E-65		not extracellular
Glycosyl Transferases	48	143054	9.00E-80		not extracellular
Glycosyl Transferases	57	108422	3.00E-23	70, 64, 69, 71, 66, 85, 133	not extracellular
Glycosyl Transferases	57	137050	8.00E-92		not extracellular
Glycosyl Transferases	58	110975	5.00E-41	75	not extracellular
Glycosyl Transferases	59	139395	5.00E-30	62, 65, 72, 67, 67, 65, 215	not extracellular
Glycosyl Transferases	62	126985	5.00E-07		not extracellular
Glycosyl Transferases	62	141320	2.00E-10	77, 101	not extracellular
Glycosyl Transferases	62	141329	1.00E-17	69	not extracellular
Glycosyl Transferases	62	144528	6.00E-07		not extracellular

Glycosyl Transferases	66	116260	1.00E-164	67, 71, 52, 30	not extracellular
Glycosyl Transferases	66	140975	0.00E+00	89	not extracellular
Glycosyl Transferases	71	128426	2.00E-09		not extracellular
Glycosyl Transferases	71	130782	4.00E-09		not extracellular
Glycosyl Transferases	71	130783	5.00E-12		not extracellular
Glycosyl Transferases	71	130784	1.00E-10		not extracellular
Glycosyl Transferases	71	130785	6.00E-11		not extracellular
Glycosyl Transferases	71	130787	5.00E-06	218, 35	not extracellular
Glycosyl Transferases	71	130788	4.00E-12		not extracellular
Glycosyl Transferases	71	130790	1.00E-12	39, 150, 240	not extracellular
Glycosyl Transferases	71	130791	2.00E-12		not extracellular
Glycosyl Transferases	71	130792	3.00E-09		not extracellular
Glycosyl Transferases	71	130794	2.00E-11		not extracellular
Glycosyl Transferases	71	130799	1.00E-09	19, 148, 138	not extracellular
Glycosyl Transferases	71	130800	3.00E-10		not extracellular
Glycosyl Transferases	71	130801	4.00E-11		not extracellular
Glycosyl Transferases	71	130803	3.00E-12		not extracellular
Glycosyl Transferases	71	130858	8.00E-08		not extracellular
Glycosyl Transferases	71	130863	5.00E-09		not extracellular
Glycosyl Transferases	71	139447	5.00E-10		not extracellular
Glycosyl Transferases	71	144840	2.00E-09		not extracellular
GlycosylTransferase	76	155373	5.00E-19	143, 30	not extracellular
Polysaccharide Lyase	1	124664	9.00E-25		cytoplasm
Polysaccharide Lyase	1	128355	7.00E-69	60	cytoplasm; mitochondria
Polysaccharide Lyase	1	128356	7.00E-53	28	extracellular
Polysaccharide Lyase	1	132218	2.00E-60		extracellular
Polysaccharide Lyase	1	132222	6.00E-65	40	extracellular
Polysaccharide Lyase	1	132225	1.00E-68		extracellular
Polysaccharide Lyase	1	132227	2.00E-54		extracellular
Polysaccharide Lyase	1	133199	2.00E-55		extracellular
Polysaccharide Lyase	1	140268	7.00E-64		extracellular
Polysaccharide Lyase	1	140269	2.00E-36	111, 78, 31	extracellular
Polysaccharide Lyase	1	140270	7.00E-64	89, 64	endoplasmic reticulum
Polysaccharide Lyase	1	140272	1.00E-68		extracellular
Polysaccharide Lyase	1	141647	4.00E-68		extracellular
Polysaccharide Lyase	1	143943	8.00E-55		extracellular
Polysaccharide Lyase	1	144171	9.00E-15		cytoplasm; nucleus
Polysaccharide Lyase	1	144172	4.00E-31	249	extracellular

Polysaccharide Lyase	1	144173	6.00E-42	44	extracellular
Polysaccharide Lyase	1	144178	1.00E-45	94	extracellular
Polysaccharide Lyase	1	144191	9.00E-56		extracellular
Polysaccharide Lyase	1	144196	8.00E-52		extracellular
Polysaccharide Lyase	1	144197	8.00E-52		extracellular
Polysaccharide Lyase	1	145119	3.00E-40		cytoplasm; nucleus
Polysaccharide Lyase	1	145391	5.00E-56		cytoplasm; nucleus
Polysaccharide Lyase	1	145395	3.00E-49		extracellular
Polysaccharide Lyase	1	145592	1.00E-51		cytoplasm; mitochondrial matrix space
Polysaccharide Lyase	3	109381	6.00E-28		extracellular
Polysaccharide Lyase	3	109550	5.00E-24		cytoplasm
Polysaccharide Lyase	3	110508	4.00E-26		cytoplasm; nucleus
Polysaccharide Lyase	3	114189	2.00E-33		cytoplasm
Polysaccharide Lyase	3	114347	7.00E-25		cytoplasm
Polysaccharide Lyase	3	122873	3.00E-15		cytoplasm
Polysaccharide Lyase	3	122879	1.00E-27	693	cytoplasm
Polysaccharide Lyase	3	132101	7.00E-32	Multiple	extracellular
Polysaccharide Lyase	3	132156	1.00E-39		extracellular
Polysaccharide Lyase	3	132157	1.00E-39		extracellular
Polysaccharide Lyase	3	132158	2.00E-37		extracellular
Polysaccharide Lyase	3	132161	4.00E-38		extracellular
Polysaccharide Lyase	3	137796	2.00E-22	22	extracellular
Polysaccharide Lyase	3	137799	1.00E-30	21	cytoplasm
Polysaccharide Lyase	3	137800	2.00E-17	26, 31	extracellular
Polysaccharide Lyase	3	140239	6.00E-36	23	extracellular
Polysaccharide Lyase	3	140241	6.00E-36	1335	extracellular
Polysaccharide Lyase	3	140498	1.00E-09		cytoplasm; mitochondrial matrix space
Polysaccharide Lyase	3	141345	6.00E-28		extracellular
Polysaccharide Lyase	3	142500	2.00E-30	3544	extracellular
Polysaccharide Lyase	3	142501	4.00E-37		extracellular
Polysaccharide Lyase	3	143281	5.00E-24		extracellular
Polysaccharide Lyase	3	144923	2.00E-36	1517	cytoplasm
Polysaccharide Lyase	3	145623	6.00E-36		cytoplasm
Polysaccharide Lyase	4	128367	1.00E-35		extracellular
Polysaccharide Lyase	4	128395	5.00E-27	573	cytoplasm
Polysaccharide Lyase	4	128396	2.00E-45		extracellular
Polysaccharide Lyase	4	128397	6.00E-06	157, 325	cytoplasm; nucleus
Polysaccharide Lyase	4	143867	8.00E-13	113, 216, 110, 237, 112, 803, 339, 52, 39	cytoplasm; mitochondrial matrix space
