# Supplementary material for: The CAZyome of Phytophthora spp.: A comprehensive analysis of the gene complement coding for carbohydrate-active enzymes in species of the genus Phytophthora
Source: BMC Genomics. 2010 Sep 28;11:525. doi: 10.1186/1471-2164-11-525 (PMC2997016; doi:10.1186/1471-2164-11-525)
Supplement: Additional file 3 — CAZy genes in P. ramorum. CAZyme-coding homologs in P. ramorum organized by their respective CAZy superfamily. 1Numbers represent intron sizes; "multiple" refers to the fact that more than 9 introns were present in the gene model. 2Most likely cellular localization predicted by SecretomeP, SignalP, or TargetP algorithms. ND, not determined. [file 1471-2164-11-525-S3.PDF]

CAZy Group	Family	Gene Identifier	E Value	Introns[1]	Predicted Cellular Localization[2]
Carbohydrate Esterase	1	73355	2.00E-46		extracelllular
Carbohydrate Esterase	4	71737	4.00E-72		cytoplasm; mitochondria
Carbohydrate Esterase	4	83860	3.00E-35	133	extracelllular
Carbohydrate Esterase	5	47458	1.00E-14		extracelllular
Carbohydrate Esterase	5	71624	1.00E-12	34	extracelllular
Carbohydrate Esterase	5	74999	4.00E-22		extracelllular
Carbohydrate Esterase	5	78496	1.00E-20		extracelllular
Carbohydrate Esterase	8	71191	7.00E-12		cytoplasm
Carbohydrate Esterase	8	71818	1.00E-07		extracelllular
Carbohydrate Esterase	8	72026	2.00E-08		extracelllular
Carbohydrate Esterase	8	72192	4.00E-11		extracelllular
Carbohydrate Esterase	8	72340	1.00E-11		cytoplasm
Carbohydrate Esterase	8	72362	1.00E-07		extracelllular
Carbohydrate Esterase	8	72489	1.00E-10		extracelllular
Carbohydrate Esterase	8	72490	1.00E-07		extracelllular
Carbohydrate Esterase	8	74392	9.00E-12		extracelllular
Carbohydrate Esterase	8	79718	3.00E-12		extracelllular
Carbohydrate Esterase	8	81929	1.00E-10		extracelllular
Carbohydrate Esterase	8	87810	3.00E-15		cytoplasm
Carbohydrate Esterase	8	87811	2.00E-29		extracelllular
Carbohydrate Esterase	10	76241	3.00E-12	63, 79	cytoplasm; mitochondria
Glycoside Hydrolase	1	52810	3.00E-44		cytoplasm
Glycoside Hydrolase	1	71118	1.00E-85		cytoplasm
Glycoside Hydrolase	1	76490	2.00E-45		extracelllular
Glycoside Hydrolase	1	76491	1.00E-42		plasma membrane
Glycoside Hydrolase	1	76494	4.00E-39		extracelllular
Glycoside Hydrolase	1	76495	2.00E-44		extracelllular
Glycoside Hydrolase	1	82341	7.00E-38		extracelllular
Glycoside Hydrolase	1	94521	4.00E-30	414, 35	ND
Glycoside Hydrolase	2	94534	4.00E-69	82,73,80,36,62,231,360	extracelllular
Glycoside Hydrolase	3	73436	7.00E-14		extracelllular
Glycoside Hydrolase	3	73438	3.00E-17	75	cytoplasm
Glycoside Hydrolase	3	73439	6.00E-17		extracelllular
Glycoside Hydrolase	3	73444	1.00E-12	220	cytoplasm; mitochondria
Glycoside Hydrolase	3	73446	3.00E-17	75	cytoplasm
Glycoside Hydrolase	3	74161	1.00E-10	21, 65, 243, 126, 20	cytoplasm; mitochondria
Glycoside Hydrolase	3	76113	2.00E-11		cytoplasm

Glycoside Hydrolase	3	77835	1.00E-13	67	extracelllular
Glycoside Hydrolase	3	78229	1.00E-14		extracelllular
Glycoside Hydrolase	3	78230	2.00E-18		extracelllular
Glycoside Hydrolase	3	78231	4.00E-18		extracelllular
Glycoside Hydrolase	3	78232	4.00E-08		extracelllular
Glycoside Hydrolase	3	78295	6.00E-09		extracelllular
Glycoside Hydrolase	3	78296	9.00E-21		extracelllular
Glycoside Hydrolase	3	78297	9.00E-21		extracelllular
Glycoside Hydrolase	3	79076	5.00E-17		extracelllular
Glycoside Hydrolase	3	79077	5.00E-17		extracelllular
Glycoside Hydrolase	3	81321	9.00E-15		cytoplasm; mitochondria
Glycoside Hydrolase	3	83174	6.00E-10		extracelllular
Glycoside Hydrolase	3	86458	8.00E-11	1027	extracelllular
Glycoside Hydrolase	3	86891	5.00E-10	61	extracelllular
Glycoside Hydrolase	3	86917	5.00E-10	914	extracelllular
Glycoside Hydrolase	3	86922	9.00E-21		extracelllular
Glycoside Hydrolase	5	73539	3.00E-70	79	cytoplasm; mitochondria
Glycoside Hydrolase	5	76972	1.00E-39		plasma membrane
Glycoside Hydrolase	5	78128	7.00E-12	62	extracelllular*
Glycoside Hydrolase	5	79037	0.00E+00	71	plasma membrane
Glycoside Hydrolase	5	79038	0.00E+00	66,68	cytoplasm
Glycoside Hydrolase	5	80684	5.00E-16		extracelllular
Glycoside Hydrolase	5	83874	1.00E-161	73, 49,59	plasma membrane
Glycoside Hydrolase	5	83875	1.00E-167		cytoplasm; mitochondria
Glycoside Hydrolase	5	83876	1.00E-113	78, 304	cytoplasm
Glycoside Hydrolase	5	86054	3.00E-47	296, 70	cytoplasm
Glycoside Hydrolase	5	87384	1.00E-131	296, 1076	cytoplasm
Glycoside Hydrolase	6	74236	2.00E-08		extracelllular
Glycoside Hydrolase	6	75965	4.00E-11		extracelllular
Glycoside Hydrolase	6	75966	8.00E-10		extracelllular
Glycoside Hydrolase	6	75967	2.00E-10		extracelllular
Glycoside Hydrolase	6	75969	3.00E-11		extracelllular
Glycoside Hydrolase	6	75973	4.00E-07	57, 88	extracelllular*
Glycoside Hydrolase	6	80136	6.00E-09	187,66	extracelllular
Glycoside Hydrolase	6	84834	7.00E-08		extracelllular
Glycoside Hydrolase	6	86772	4.00E-11		extracelllular
Glycoside Hydrolase	6	93598	3.00E-07	24, 455, 30, 185	extracelllular
Glycoside Hydrolase	7	53490	3.00E-55		extracelllular

Glycoside Hydrolase	7	71345	7.00E-60	196	extracellular
Glycoside Hydrolase	7	72059	3.00E-57		extracelllular
Glycoside Hydrolase	7	72061	5.00E-45	48	cytoplasm
Glycoside Hydrolase	10	74308	7.00E-40		extracelllular
Glycoside Hydrolase	10	74319	1.00E-34	74, 71	extracelllular
Glycoside Hydrolase	10	83132	8.00E-30	70, 76	extracelllular
Glycoside Hydrolase	10	83134	1.00E-30	70, 69	extracelllular
Glycoside Hydrolase	10	84765	5.00E-17	74, 80, 165, 52	cytoplasm
Glycoside Hydrolase	10	85409	2.00E-35	70, 74, 80	extracelllular
Glycoside Hydrolase	10	93440	2.00E-18	159, 241	cytoplasm
Glycoside Hydrolase	12	49047	2.00E-95		cytoplasm
Glycoside Hydrolase	12	52767	1.00E-122		cytoplasm
Glycoside Hydrolase	12	72030	1.00E-144		extracelllular
Glycoside Hydrolase	12	72080	6.00E-70		extracelllular
Glycoside Hydrolase	12	72081	4.00E-68		extracelllular
Glycoside Hydrolase	12	79939	9.00E-85		extracelllular
Glycoside Hydrolase	12	83138	9.00E-51		extracelllular
Glycoside Hydrolase	12	83139	1.00E-81		extracelllular
Glycoside Hydrolase	17	72319	9.00E-24		extracelllular
Glycoside Hydrolase	17	72578	1.00E-06		extracelllular
Glycoside Hydrolase	17	72940	6.00E-26		cytoplasm
Glycoside Hydrolase	17	72941	9.00E-17		extracelllular
Glycoside Hydrolase	17	72942	9.00E-17		extracelllular
Glycoside Hydrolase	17	81925	2.00E-17		extracelllular
Glycoside Hydrolase	17	85261	2.00E-22		extracelllular
Glycoside Hydrolase	17	87481	6.00E-14	1462	extracelllular
Glycoside Hydrolase	19	71328	2.00E-19	48	extracelllular
Glycoside Hydrolase	28	40980	5.00E-15		cytoplasm
Glycoside Hydrolase	28	41820	2.00E-20		extracelllular
Glycoside Hydrolase	28	41921	9.00E-39		cytoplasm
Glycoside Hydrolase	28	48642	6.00E-16		cytoplasm
Glycoside Hydrolase	28	54493	1.00E-20		extracelllular
Glycoside Hydrolase	28	54527	3.00E-19		cytoplasm
Glycoside Hydrolase	28	72130	5.00E-27	58, 60	extracelllular
Glycoside Hydrolase	28	72132	7.00E-21		extracelllular
Glycoside Hydrolase	28	72133	1.00E-17	124	cytoplasm
Glycoside Hydrolase	28	72134	3.00E-20		extracelllular
Glycoside Hydrolase	28	72135	7.00E-17		extracelllular

Glycoside Hydrolase	28	83562	3.00E-21	62	extracelllular
Glycoside Hydrolase	28	83564	3.00E-21		extracelllular
Glycoside Hydrolase	28	83565	3.00E-21		extracelllular
Glycoside Hydrolase	28	83568	1.00E-20		extracelllular
Glycoside Hydrolase	28	83569	2.00E-20	140	extracelllular
Glycoside Hydrolase	28	83573	2.00E-85	58	extracelllular
Glycoside Hydrolase	28	83574	5.00E-17	64, 31	extracelllular
Glycoside Hydrolase	30	51732	1.00E-170		lysosome
Glycoside Hydrolase	30	51749	0.00E+00		lysosome
Glycoside Hydrolase	30	51757	0.00E+00		lysosome
Glycoside Hydrolase	30	52439	1.00E-152		lysosome
Glycoside Hydrolase	30	81296	2.00E-59		lysosome
Glycoside Hydrolase	30	81297	9.00E-57		lysosome
Glycoside Hydrolase	30	81298	2.00E-06	125	lysosome
Glycoside Hydrolase	30	81300	9.00E-57		lysosome
Glycoside Hydrolase	30	81639	1.00E-149		lysosome
Glycoside Hydrolase	30	81640	1.00E-149		lysosome
Glycoside Hydrolase	30	81642	0.00E+00		lysosome
Glycoside Hydrolase	31	73317	4.00E-31		cytoplasm; mitochondria
Glycoside Hydrolase	31	76244	1.00E-50		plasma membrane
Glycoside Hydrolase	31	76755	1.00E-122		extracelllular
Glycoside Hydrolase	31	76757	1.00E-121		extracelllular
Glycoside Hydrolase	32	82583	2.00E-35		extracelllular
Glycoside Hydrolase	32	82584	4.00E-36		extracelllular
Glycoside Hydrolase	35	96749	1.00E-13	124, 42, 55, 64, 64, 597, 57	cytoplasm; mitochondria
Glycoside Hydrolase	37	82922	2.00E-62	124, 315	extracelllular
Glycoside Hydrolase	37	82934	6.00E-62		cytoplasm
Glycoside Hydrolase	47	40131	1.00E-08		cytoplasm; mitochondria
Glycoside Hydrolase	47	45104	3.00E-27	69, 171, 67, 80	cytoplasm
Glycoside Hydrolase	47	45108	4.00E-27	67,79, 69, 153	cytoplasm
Glycoside Hydrolase	47	49663	2.00E-32		cytoplasm
Glycoside Hydrolase	47	50011	4.00E-50	93, 79, 68	cytoplasm
Glycoside Hydrolase	47	53914	1.00E-37	61, 72 77	cytoplasm; mitochondria
Glycoside Hydrolase	53	76938	4.00E-20	537	extracelllular
Glycoside Hydrolase	53	76939	3.00E-07	58	extracelllular
Glycoside Hydrolase	53	76940	4.00E-29	85	extracelllular
Glycoside Hydrolase	53	76942	4.00E-29	85	extracelllular
Glycoside Hydrolase	53	76943	2.00E-27	67	extracelllular

Glycoside Hydrolase	53	76944	1.00E-25	64	extracelllular
Glycoside Hydrolase	54	72148	1.00E-113	26	extracelllular
Glycoside Hydrolase	63	54464	1.00E-18		cytoplasm; mitochondria
Glycoside Hydrolase	63	81129	7.00E-13	71	cytoplasm
Glycoside Hydrolase	63	84565	2.00E-82		cytoplasm; mitochondria
Glycoside Hydrolase	72	81632	5.00E-08		extracelllular
Glycoside Hydrolase	72	81633	5.00E-08		extracelllular
Glycoside Hydrolase	72	83721	1.00E-09	Multiple	extracelllular*
Glycoside Hydrolase	78	73895	3.00E-06		extracelllular
Glycoside Hydrolase	78	73896	2.00E-08	92	extracelllular
Glycoside Hydrolase	81	74498	7.00E-68		cytoplasm; mitochondria
Glycoside Hydrolase	81	74531	3.00E-65		extracelllular
Glycoside Hydrolase	81	74534	3.00E-65		extracelllular
Glycoside Hydrolase	81	75292	6.00E-59		cytoplasm
Glycoside Hydrolase	81	81354	3.00E-57		cytoplasm; mitochondria
Glycoside Hydrolase	81	81355	3.00E-62	125	extracelllular*
Glycoside Hydrolase	81	81368	2.00E-66	341, 445, 77, 73, 94, 119, 88	extracelllular
Glycoside Hydrolase	81	81371	1.00E-67		cytoplasm
Glycoside Hydrolase	81	81385	6.00E-66		extracelllular
Glycoside Hydrolase	81	83712	2.00E-51	31, 76, 70	cytoplasm; nucleus
Glycoside Hydrolase	81	84172	1.00E-59	205, 72, 77	cytoplasm
Glycoside Hydrolase	81	85960	4.00E-67		cytoplasm; mitochondria
Glycoside Hydrolase	81	86971	3.00E-38	301, 73	extracelllular*
Glycoside Hydrolase	85	79858	1.00E-70	77, 63	cytoplasm
Glycoside Hydrolase	89	80249	1.00E-140	68, 82, 386, 70	extracelllular
Glycoside Hydrolase	89	95635	1.00E-131	48, 324, 57, 60	extracelllular
Glycoside Hydrolase	95	71121	6.00E-89		cytoplasm
Glycoside Hydrolase	95	71383	3.00E-28		cytoplasm
Glycoside Hydrolase	95	73653	1.00E-82		cytoplasm
Glycoside Hydrolase	95	75725	2.00E-44		cytoplasm; mitochondria
Glycoside Hydrolase	95	76492	1.00E-36		plasma membrane
Glycoside Hydrolase	95	76529	2.00E-47		extracelllular
Glycoside Hydrolase	95	79042	3.00E-37		extracelllular
Glycoside Hydrolase	95	83519	3.00E-51		extracelllular
Glycoside Hydrolase	95	83520	3.00E-51		extracelllular
Glycoside Hydrolase	95	94544	3.00E-42		plasma membrane
Glycoside Hydrolase	105	83050	2.00E-39		extracelllular
Glycosyl Transferasess	1	40606	1.00E-103		not extracellular

Glycosyl Transferasess	1	79867	6.00E-07	300	not extracellular
Glycosyl Transferasess	1	80101	1.00E-106	66,128, 31, 687, 47	not extracellular
Glycosyl Transferasess	1	84481	1.00E-104		not extracellular
Glycosyl Transferasess	1	84690	1.00E-103	161, 78, 42, 135, 19	not extracellular
Glycosyl Transferasess	1	87277	3.00E-29		not extracellular
Glycosyl Transferasess	1	96916	1.00E-103	114	not extracellular
Glycosyl Transferasess	2	72819	1.00E-08	105, 50, 101, 130	not extracellular
Glycosyl Transferasess	2	73096	5.00E-07	72, 69, 79	not extracellular
Glycosyl Transferasess	2	74004	9.00E-07	151, 80, 170, 72	not extracellular
Glycosyl Transferasess	2	75334	1.00E-07		not extracellular
Glycosyl Transferasess	2	77229	3.00E-07	313, 58	not extracellular
Glycosyl Transferasess	2	81287	5.00E-08	68, 77, 82	not extracellular
Glycosyl Transferasess	2	84041	2.00E-79	68, 120, 30, 52, 70, 67, 118	not extracellular
Glycosyl Transferasess	2	86403	5.00E-08	29	not extracellular
Glycosyl Transferasess	4	72636	5.00E-58	64, 71, 78	not extracellular
Glycosyl Transferasess	20	71457	1.00E-76	70	not extracellular
Glycosyl Transferasess	20	73849	5.00E-40	68, 92, 430, 326, 71	not extracellular
Glycosyl Transferasess	20	77528	5.00E-47	63, 72, 63, 203	not extracellular
Glycosyl Transferasess	20	80488	2.00E-58		not extracellular
Glycosyl Transferasess	20	81662	2.00E-70		not extracellular
Glycosyl Transferasess	20	82938	6.00E-49		not extracellular
Glycosyl Transferasess	20	84498	3.00E-37		not extracellular
Glycosyl Transferasess	20	85749	9.00E-73		not extracellular
Glycosyl Transferasess	22	71676	4.00E-73		not extracellular
Glycosyl Transferasess	24	94673	1.00E-149	48, 105	not extracellular
Glycosyl Transferasess	33	46727	4.00E-72	93, 66, 80, 65, 70	not extracellular
Glycosyl Transferasess	41	74404	1.00E-08	69, 614, 216, 91, 105	not extracellular
Glycosyl Transferasess	41	75562	1.00E-117	64	not extracellular
Glycosyl Transferasess	41	94933	5.00E-18	162, 117, 183	not extracellular
Glycosyl Transferasess	41	96233	2.00E-14	64, 172, 228, 84, 363, 60, 76, 204, 79	not extracellular
Glycosyl Transferasess	48	76056	5.00E-87	210, 81	not extracellular
Glycosyl Transferasess	48	76057	6.00E-93		not extracellular
Glycosyl Transferasess	48	76180	4.00E-87		not extracellular
Glycosyl Transferasess	48	77350	3.00E-75		not extracellular
Glycosyl Transferasess	48	77994	2.00E-62		not extracellular
Glycosyl Transferasess	48	80843	7.00E-81	154	not extracellular
Glycosyl Transferasess	48	82372	1.00E-77		not extracellular
Glycosyl Transferasess	57	43914	2.00E-12	83	not extracellular

Glycosyl Transferasess	57	82508	7.00E-41	25	not extracellular
Glycosyl Transferasess	57	87257	1.00E-33		not extracellular
Glycosyl Transferasess	58	45645	6.00E-44	73, 84	not extracellular
Glycosyl Transferasess	59	77928	1.00E-29	61, 67, 70, 68, 78, 74, 308, 200	not extracellular
Glycosyl Transferasess	61	78730	4.00E-06		not extracellular
Glycosyl Transferasess	61	78738	3.00E-08		not extracellular
Glycosyl Transferasess	62	74802	7.00E-08	65, 482, 82	not extracellular
Glycosyl Transferasess	62	83780	1.00E-10	74	not extracellular
Glycosyl Transferasess	62	93922	4.00E-16	54, 67	not extracellular
Glycosyl Transferasess	66	38947	1.00E-159	88, 71	not extracellular
Glycosyl Transferasess	66	71487	8.00E-21	263	not extracellular
Glycosyl Transferasess	66	72878	0.00E+00	80	not extracellular
Glycosyl Transferasess	71	73611	2.00E-15		not extracellular
Glycosyl Transferasess	71	73613	7.00E-22	22	not extracellular
Glycosyl Transferasess	71	73614	3.00E-15		not extracellular
Glycosyl Transferasess	71	73615	7.00E-24		not extracellular
Glycosyl Transferasess	71	73616	2.00E-24	249	not extracellular
Glycosyl Transferasess	71	73619	2.00E-14	171, 219, 528, 37	not extracellular
Glycosyl Transferasess	71	73621	7.00E-10	146	not extracellular
Glycosyl Transferasess	71	73624	1.00E-11	127	not extracellular
Glycosyl Transferasess	71	73625	2.00E-19	110, 60, 32	not extracellular
Glycosyl Transferasess	71	73626	3.00E-19		not extracellular
Glycosyl Transferasess	71	73627	3.00E-22		not extracellular
Glycosyl Transferasess	71	73628	2.00E-15		not extracellular
Glycosyl Transferasess	71	73629	4.00E-16		not extracellular
Glycosyl Transferasess	71	73630	2.00E-13		not extracellular
Glycosyl Transferasess	71	76613	9.00E-15		not extracellular
Glycosyl Transferasess	71	78112	3.00E-18		not extracellular
Glycosyl Transferasess	71	78113	5.00E-17		not extracellular
Glycosyl Transferasess	71	80481	2.00E-12	314	not extracellular
Glycosyl Transferasess	71	81914	1.00E-11		not extracellular
Glycosyl Transferasess	71	82055	6.00E-07	69	not extracellular
Glycosyl Transferasess	71	93659	1.00E-24	27	not extracellular
Glycosyl Transferasess	71	95021	4.00E-14		not extracellular
Glycosyl Transferasess	71	95737	7.00E-10	21bp, 80	not extracellular
Glycosyl Transferasess	76	94555	7.00E-12	203, 225	not extracellular
Polysaccharide Lyase	1	39775	7.00E-56		extracelllular
Polysaccharide Lyase	1	45942	1.00E-67		extracelllular

Polysaccharide Lyase	1	46219	2.00E-55		extracelllular
Polysaccharide Lyase	1	75225	1.00E-52		extracelllular
Polysaccharide Lyase	1	75226	3.00E-53		extracelllular
Polysaccharide Lyase	1	75227	9.00E-66		extracelllular
Polysaccharide Lyase	1	75231	6.00E-55		extracelllular
Polysaccharide Lyase	1	75233	6.00E-12		ND
Polysaccharide Lyase	1	77010	4.00E-67		extracelllular
Polysaccharide Lyase	1	77715	3.00E-72		extracelllular
Polysaccharide Lyase	1	85622	4.00E-60		extracelllular
Polysaccharide Lyase	1	85623	2.00E-64		extracelllular
Polysaccharide Lyase	1	85625	5.00E-37	192, 63, 221, 21	cytoplasm; mitochondria
Polysaccharide Lyase	1	85626	9.00E-52	65	extracelllular
Polysaccharide Lyase	1	86921	5.00E-65		cytoplasm
Polysaccharide Lyase	1	87344	7.00E-36	1733	extracelllular
Polysaccharide Lyase	3	39059	9.00E-45		cytoplasm
Polysaccharide Lyase	3	39825	2.00E-49		ND
Polysaccharide Lyase	3	42333	2.00E-37		extracelllular
Polysaccharide Lyase	3	42787	9.00E-45		cytoplasm
Polysaccharide Lyase	3	43398	7.00E-41		extracelllular
Polysaccharide Lyase	3	44445	4.00E-47		cytoplasm
Polysaccharide Lyase	3	44446	2.00E-42		extracelllular
Polysaccharide Lyase	3	44483	1.00E-36		extracelllular
Polysaccharide Lyase	3	44485	1.00E-41		cytoplasm
Polysaccharide Lyase	3	44487	1.00E-36		extracelllular
Polysaccharide Lyase	3	44512	4.00E-40		extracelllular
Polysaccharide Lyase	3	46251	2.00E-50		extracelllular
Polysaccharide Lyase	3	71773	2.00E-53		extracelllular
Polysaccharide Lyase	3	74820	2.00E-41		extracelllular
Polysaccharide Lyase	3	74998	2.00E-49		extracelllular
Polysaccharide Lyase	3	77022	8.00E-43		extracelllular
Polysaccharide Lyase	3	77024	4.00E-42	97	extracelllular
Polysaccharide Lyase	3	77029	4.00E-40		extracelllular
Polysaccharide Lyase	3	80133	2.00E-53		extracelllular
Polysaccharide Lyase	3	82586	5.00E-42		extracelllular
Polysaccharide Lyase	3	82590	2.00E-42		extracelllular
Polysaccharide Lyase	3	82591	1.00E-42	137, 681, 313, 152, 76	extracelllular
Polysaccharide Lyase	3	84933	4.00E-13		extracelllular
Polysaccharide Lyase	3	86169	4.00E-42	645	extracelllular

Polysaccharide Lyase	3	87411	2.00E-41	extracelllular
Polysaccharide Lyase	4	72397	2.00E-44	extracelllular
Polysaccharide Lyase	4	72424	3.00E-15	cytoplasm
Polysaccharide Lyase	4	84661	3.00E-45	extracelllular
Polysaccharide Lyase	4	84662	2.00E-46	extracelllular
Polysaccharide Lyase	4	84663	8.00E-53	extracelllular
Polysaccharide Lyase	4	86292	8.00E-53	extracelllular
