# Supplementary material for: The CAZyome of Phytophthora spp.: A comprehensive analysis of the gene complement coding for carbohydrate-active enzymes in species of the genus Phytophthora
Source: BMC Genomics. 2010 Sep 28;11:525. doi: 10.1186/1471-2164-11-525 (PMC2997016; doi:10.1186/1471-2164-11-525)
Supplement: Additional file 4 — Phytophthora CE orthologs. Phytophthora CE orthologs as determined using the Phylogenetic Resources for the Interpretation of Genomes (PHRINGE). All, but one, of the CE homologs had orthologs in both P. sojae and P. ramorum genomes. In most cases, more than one orthologous gene was found in each of the three Phytophthora species. [file 1471-2164-11-525-S4.PDF]

CARBOHYDRATE ESTERASES

Gene Identifier	Matching Gene	Source Organism	Length	Difference in Length	Distance Score
PITG_00041	Pra72340	Phytophthora ramorum	321	204	0.474671
PITG_00041	Pra79718	Phytophthora ramorum	349	232	0.487266
PITG_00041	Pra71191	Phytophthora ramorum	316	199	0.523225
PITG_00041	Pra81929	Phytophthora ramorum	345	228	0.531919
PITG_00041	Pra87811	Phytophthora ramorum	275	158	0.555488
PITG_00041	Pso131634	Phytophthora sojae	235	118	0.394364
PITG_00041	Pso127157	Phytophthora sojae	350	233	0.49204
PITG_00041	Pso134354	Phytophthora sojae	343	226	0.508644
PITG_00041	Pso131657	Phytophthora sojae	335	218	0.563016
PITG_00041	Pso144377	Phytophthora sojae	307	190	0.575866
PITG_01029	Pra72026	Phytophthora ramorum	344	5	0.293667
PITG_01029	Pra72192	Phytophthora ramorum	344	5	0.368627
PITG_01029	Pra79718	Phytophthora ramorum	349	10	0.399008
PITG_01029	Pra87811	Phytophthora ramorum	275	64	0.441061
PITG_01029	Pra74392	Phytophthora ramorum	344	5	0.443716
PITG_01029	Pso127162	Phytophthora sojae	245	94	0.305112
PITG_01029	Pso144376	Phytophthora sojae	343	4	0.311193
PITG_01029	Pso127156	Phytophthora sojae	333	6	0.334138
PITG_01029	Pso108139	Phytophthora sojae	340	1	0.357637
PITG_01029	Pso127157	Phytophthora sojae	350	11	0.361979
PITG_01502	Pra96173	Phytophthora ramorum	1222	61	0.094782
PITG_01502	Pso127449	Phytophthora sojae	1222	61	0.090948
PITG_02274	Pra49673	Phytophthora ramorum	263	30	0.164627
PITG_02274	Pso108278	Phytophthora sojae	144	149	0.03442
PITG_02274	Pso128451	Phytophthora sojae	134	159	0.086938
PITG_02274	Pso128450	Phytophthora sojae	295	2	0.323619
PITG_02483	Pra77612	Phytophthora ramorum	338	1	0.067447
PITG_02483	Pra75138	Phytophthora ramorum	338	1	0.483942
PITG_02483	Pso128282	Phytophthora sojae	338	1	0.073185
PITG_02483	Pso133206	Phytophthora sojae	338	1	0.519232
PITG_02504	Pra71533	Phytophthora ramorum	131	83	0.187686
PITG_02504	Pso128255	Phytophthora sojae	356	142	0.979683
PITG_02545	Pra72026	Phytophthora ramorum	344	5	0.336422
PITG_02545	Pra72192	Phytophthora ramorum	344	5	0.430611
PITG_02545	Pra87810	Phytophthora ramorum	147	192	0.471067
PITG_02545	Pra79718	Phytophthora ramorum	349	10	0.481958
PITG_02545	Pra72340	Phytophthora ramorum	321	18	0.4865

PITG_02545	Pso127162	Phytophthora sojae	245	94	0.320244
PITG_02545	Pso127156	Phytophthora sojae	333	6	0.335581
PITG_02545	Pso144376	Phytophthora sojae	343	4	0.348446
PITG_02545	Pso108139	Phytophthora sojae	340	1	0.362937
PITG_02545	Pso127157	Phytophthora sojae	350	11	0.411897
PITG_02607	Pra94820	Phytophthora ramorum	290	0	0.033407
PITG_02607	Pra51464	Phytophthora ramorum	254	36	0.794309
PITG_02607	Pso109610	Phytophthora sojae	288	2	0.062838
PITG_02607	Pso120335	Phytophthora sojae	260	30	0.75546
PITG_02607	Pso143153	Phytophthora sojae	422	132	0.884286
PITG_02899	Pra85068	Phytophthora ramorum	238	10	0.278376
PITG_02899	Pra86913	Phytophthora ramorum	280	32	0.284006
PITG_02899	Pra80529	Phytophthora ramorum	280	32	0.35286
PITG_02899	Pra80534	Phytophthora ramorum	280	32	0.479702
PITG_02899	Pso134972	Phytophthora sojae	276	28	0.332945
PITG_02899	Pso136282	Phytophthora sojae	229	19	0.373772
PITG_02899	Pso136281	Phytophthora sojae	321	73	0.45661
PITG_02899	Pso136285	Phytophthora sojae	3150	2902	0.487032
PITG_02902	Pra86913	Phytophthora ramorum	280	21	0.208008
PITG_02902	Pra85068	Phytophthora ramorum	238	21	0.283301
PITG_02902	Pra80529	Phytophthora ramorum	280	21	0.419915
PITG_02902	Pra80534	Phytophthora ramorum	280	21	0.548058
PITG_02902	Pso136285	Phytophthora sojae	3150	2891	0.215272
PITG_02902	Pso134972	Phytophthora sojae	276	17	0.223483
PITG_02902	Pso136281	Phytophthora sojae	321	62	0.519787
PITG_02902	Pso136282	Phytophthora sojae	229	30	0.632538
PITG_03463	Pra71382	Phytophthora ramorum	283	15	0.054175
PITG_03463	Pso108753	Phytophthora sojae	283	15	0.043775
PITG_03543	Pra81374	Phytophthora ramorum	459	37	0.187728
PITG_03543	Pra85150	Phytophthora ramorum	176	246	0.695076
PITG_03543	Pra84523	Phytophthora ramorum	532	110	0.777288
PITG_03543	Pra73609	Phytophthora ramorum	418	4	0.789237
PITG_03543	Pra84522	Phytophthora ramorum	489	67	0.826283
PITG_03543	Pso134789	Phytophthora sojae	459	37	0.188394
PITG_03543	Pso134790	Phytophthora sojae	480	58	0.396012
PITG_03543	Pso134786	Phytophthora sojae	203	219	0.49663
PITG_03543	Pso138874	Phytophthora sojae	591	169	0.762738
PITG_03543	Pso138875	Phytophthora sojae	478	56	0.787561
PITG_03840	Pra94134	Phytophthora ramorum	1458	642	0.186751

PITG_03840	Pso157453	Phytophthora sojae	1540	724	0.183748
PITG_04325	Pra72340	Phytophthora ramorum	321	30	0.226622
PITG_04325	Pra71191	Phytophthora ramorum	316	35	0.245814
PITG_04325	Pra74392	Phytophthora ramorum	344	7	0.256062
PITG_04325	Pra72192	Phytophthora ramorum	344	7	0.454255
PITG_04325	Pra72026	Phytophthora ramorum	344	7	0.471536
PITG_04325	Pso133883	Phytophthora sojae	343	8	0.241589
PITG_04325	Pso128784	Phytophthora sojae	345	6	0.513121
PITG_04325	Pso127162	Phytophthora sojae	245	106	0.51886
PITG_04325	Pso127156	Phytophthora sojae	333	18	0.5209
PITG_04325	Pso128733	Phytophthora sojae	344	7	0.525905
PITG_04874	Pra75509	Phytophthora ramorum	2054	1761	0.071575
PITG_04874	Pso142493	Phytophthora sojae	1534	1241	0.059884
PITG_05542	Pra71082	Phytophthora ramorum	257	0	0.060341
PITG_05542	Pso108423	Phytophthora sojae	257	0	0.04471
PITG_06560	Pra81929	Phytophthora ramorum	345	1	0.142292
PITG_06560	Pra72340	Phytophthora ramorum	321	25	0.409737
PITG_06560	Pra71191	Phytophthora ramorum	316	30	0.436016
PITG_06560	Pra74392	Phytophthora ramorum	344	2	0.437557
PITG_06560	Pra72192	Phytophthora ramorum	344	2	0.464037
PITG_06560	Pso128784	Phytophthora sojae	345	1	0.138457
PITG_06560	Pso128733	Phytophthora sojae	344	2	0.178544
PITG_06560	Pso133883	Phytophthora sojae	343	3	0.485507
PITG_06560	Pso127156	Phytophthora sojae	333	13	0.503801
PITG_06560	Pso134353	Phytophthora sojae	294	52	0.508749
PITG_06891	Pra71745	Phytophthora ramorum	283	0	0.057551
PITG_06891	Pso109641	Phytophthora sojae	283	0	0.046716
PITG_06969	Pra95562	Phytophthora ramorum	514	255	0.079303
PITG_06969	Pso157895	Phytophthora sojae	471	212	0.147136
PITG_07333	Pra77171	Phytophthora ramorum	429	67	0.185093
PITG_07333	Pra74165	Phytophthora ramorum	426	64	0.633395
PITG_07333	Pra77173	Phytophthora ramorum	421	59	0.690005
PITG_07333	Pra77172	Phytophthora ramorum	425	63	0.705977
PITG_07333	Pso129280	Phytophthora sojae	427	65	0.219121
PITG_07333	Pso129286	Phytophthora sojae	174	188	0.421481
PITG_07333	Pso128517	Phytophthora sojae	382	20	0.63785
PITG_07333	Pso129288	Phytophthora sojae	195	167	0.677615
PITG_07333	Pso129296	Phytophthora sojae	426	64	0.723295
PITG_07334	Pra77173	Phytophthora ramorum	421	98	0.132334

PITG_07334	Pra74165	Phytophthora ramorum	426	103	0.210083
PITG_07334	Pra77172	Phytophthora ramorum	425	102	0.351306
PITG_07334	Pra77171	Phytophthora ramorum	429	106	0.540818
PITG_07334	Pso129286	Phytophthora sojae	174	149	0.129127
PITG_07334	Pso129296	Phytophthora sojae	426	103	0.143262
PITG_07334	Pso129288	Phytophthora sojae	195	128	0.257342
PITG_07334	Pso128517	Phytophthora sojae	382	59	0.327574
PITG_07334	Pso129285	Phytophthora sojae	103	220	0.376661
PITG_07354	Pra95920	Phytophthora ramorum	767	22	0.170729
PITG_07354	Pso141725	Phytophthora sojae	789	0	0.161748
PITG_08056	Pra84015	Phytophthora ramorum	723	346	0.232466
PITG_08056	Pso143035	Phytophthora sojae	756	379	0.208733
PITG_08421	Pra51464	Phytophthora ramorum	254	61	0.086885
PITG_08421	Pra94820	Phytophthora ramorum	290	25	0.877879
PITG_08421	Pso120335	Phytophthora sojae	260	55	0.128508
PITG_08421	Pso109610	Phytophthora sojae	288	27	0.898434
PITG_08590	Pra83860	Phytophthora ramorum	326	45	0.218583
PITG_08590	Pso132197	Phytophthora sojae	317	36	0.225423
PITG_08863	Pra71818	Phytophthora ramorum	349	118	0.352789
PITG_08863	Pra72490	Phytophthora ramorum	349	118	0.352789
PITG_08863	Pra72362	Phytophthora ramorum	349	118	0.352789
PITG_08863	Pra87811	Phytophthora ramorum	275	44	0.354196
PITG_08863	Pra72192	Phytophthora ramorum	344	113	0.423074
PITG_08863	Pso143743	Phytophthora sojae	348	117	0.463532
PITG_08863	Pso144376	Phytophthora sojae	343	112	0.482979
PITG_08863	Pso128784	Phytophthora sojae	345	114	0.514082
PITG_08863	Pso134354	Phytophthora sojae	343	112	0.522277
PITG_08863	Pso131657	Phytophthora sojae	335	104	0.530157
PITG_08902	Pra41915	Phytophthora ramorum	166	736	0.162809
PITG_08902	Pra87810	Phytophthora ramorum	147	755	0.36164
PITG_08902	Pra39077	Phytophthora ramorum	150	752	0.39394
PITG_08902	Pra54706	Phytophthora ramorum	132	770	0.45963
PITG_08902	Pra39874	Phytophthora ramorum	132	770	0.488106
PITG_08902	Pso144380	Phytophthora sojae	171	731	0.382591
PITG_08902	Pso119010	Phytophthora sojae	105	797	0.41541
PITG_08902	Pso109960	Phytophthora sojae	264	638	0.443296
PITG_08902	Pso117650	Phytophthora sojae	202	700	0.603303
PITG_08902	Pso127162	Phytophthora sojae	245	657	0.631757
PITG_08910	Pra87811	Phytophthora ramorum	275	40	0.311549

PITG_08910	Pra72362	Phytophthora ramorum	349	114	0.349807
PITG_08910	Pra71818	Phytophthora ramorum	349	114	0.349807
PITG_08910	Pra72490	Phytophthora ramorum	349	114	0.349807
PITG_08910	Pra72192	Phytophthora ramorum	344	109	0.367536
PITG_08910	Pso134353	Phytophthora sojae	294	59	0.403825
PITG_08910	Pso144380	Phytophthora sojae	171	64	0.422516
PITG_08910	Pso134354	Phytophthora sojae	343	108	0.423274
PITG_08910	Pso127156	Phytophthora sojae	333	98	0.424821
PITG_08910	Pso143743	Phytophthora sojae	348	113	0.427524
PITG_08911	Pra87810	Phytophthora ramorum	147	47	0.326213
PITG_08911	Pra71818	Phytophthora ramorum	349	155	0.366722
PITG_08911	Pra72490	Phytophthora ramorum	349	155	0.366722
PITG_08911	Pra72362	Phytophthora ramorum	349	155	0.366722
PITG_08911	Pra72489	Phytophthora ramorum	349	155	0.378788
PITG_08911	Pso134354	Phytophthora sojae	343	149	0.450325
PITG_08911	Pso143743	Phytophthora sojae	348	154	0.451314
PITG_08911	Pso133883	Phytophthora sojae	343	149	0.485162
PITG_08911	Pso128784	Phytophthora sojae	345	151	0.493632
PITG_08911	Pso128733	Phytophthora sojae	344	150	0.498792
PITG_08912	Pra87811	Phytophthora ramorum	275	77	0.224831
PITG_08912	Pra72490	Phytophthora ramorum	349	3	0.236094
PITG_08912	Pra72362	Phytophthora ramorum	349	3	0.236094
PITG_08912	Pra71818	Phytophthora ramorum	349	3	0.236094
PITG_08912	Pra87810	Phytophthora ramorum	147	205	0.271591
PITG_08912	Pso144380	Phytophthora sojae	171	181	0.349647
PITG_08912	Pso134354	Phytophthora sojae	343	9	0.454317
PITG_08912	Pso143743	Phytophthora sojae	348	4	0.454897
PITG_08912	Pso137540	Phytophthora sojae	114	238	0.471697
PITG_08912	Pso131657	Phytophthora sojae	335	17	0.472016
PITG_08914	Pra87811	Phytophthora ramorum	275	5	0.210934
PITG_08914	Pra71818	Phytophthora ramorum	349	79	0.27036
PITG_08914	Pra72490	Phytophthora ramorum	349	79	0.27036
PITG_08914	Pra72362	Phytophthora ramorum	349	79	0.27036
PITG_08914	Pra72489	Phytophthora ramorum	349	79	0.355817
PITG_08914	Pso134353	Phytophthora sojae	294	24	0.452239
PITG_08914	Pso144376	Phytophthora sojae	343	73	0.474387
PITG_08914	Pso143743	Phytophthora sojae	348	78	0.474895
PITG_08914	Pso134354	Phytophthora sojae	343	73	0.479568
PITG_08914	Pso128784	Phytophthora sojae	345	75	0.492685

PITG_10850	Pra76241	Phytophthora ramorum	712	2	0.088088
PITG_10850	Pso141459	Phytophthora sojae	695	19	0.058412
PITG_10932	Pra74448	Phytophthora ramorum	809	21	0.145262
PITG_10932	Pso140047	Phytophthora sojae	1018	230	0.115973
PITG_11976	Pra71585	Phytophthora ramorum	309	5	0.230376
PITG_11976	Pso108511	Phytophthora sojae	317	3	0.183691
PITG_12361	Pra47458	Phytophthora ramorum	216	0	0.181544
PITG_12361	Pra71624	Phytophthora ramorum	225	9	0.182989
PITG_12361	Pra74999	Phytophthora ramorum	218	2	0.211657
PITG_12361	Pra78496	Phytophthora ramorum	215	1	0.236389
PITG_12361	Pso127975	Phytophthora sojae	218	2	0.149617
PITG_12361	Pso127977	Phytophthora sojae	218	2	0.149617
PITG_12361	Pso127974	Phytophthora sojae	218	2	0.218211
PITG_12361	Pso127976	Phytophthora sojae	218	2	0.218211
PITG_12361	Pso127983	Phytophthora sojae	215	1	0.24147
PITG_12401	Pra47458	Phytophthora ramorum	216	0	0.199437
PITG_12401	Pra71624	Phytophthora ramorum	225	9	0.200965
PITG_12401	Pra74999	Phytophthora ramorum	218	2	0.228968
PITG_12401	Pra78496	Phytophthora ramorum	215	1	0.255647
PITG_12401	Pso127975	Phytophthora sojae	218	2	0.166507
PITG_12401	Pso127977	Phytophthora sojae	218	2	0.166507
PITG_12401	Pso127974	Phytophthora sojae	218	2	0.236933
PITG_12401	Pso127976	Phytophthora sojae	218	2	0.236933
PITG_12401	Pso127983	Phytophthora sojae	215	1	0.261203
PITG_12832	Pra74999	Phytophthora ramorum	218	0	0.18761
PITG_12832	Pra47458	Phytophthora ramorum	216	2	0.263358
PITG_12832	Pra71624	Phytophthora ramorum	225	7	0.271062
PITG_12832	Pra78496	Phytophthora ramorum	215	3	0.303502
PITG_12832	Pso143280	Phytophthora sojae	227	9	0.179155
PITG_12832	Pso143279	Phytophthora sojae	218	0	0.179383
PITG_12832	Pso127983	Phytophthora sojae	215	3	0.250491
PITG_12832	Pso127977	Phytophthora sojae	218	0	0.263395
PITG_12832	Pso127975	Phytophthora sojae	218	0	0.263395
PITG_14190	Pra83701	Phytophthora ramorum	444	1	0.191963
PITG_14190	Pra85399	Phytophthora ramorum	444	1	0.195549
PITG_14190	Pso138444	Phytophthora sojae	495	52	0.192886
PITG_14194	Pra96650	Phytophthora ramorum	518	66	0.283753
PITG_14194	Pra82571	Phytophthora ramorum	376	76	0.710236
PITG_14194	Pra82570	Phytophthora ramorum	439	13	0.92147

PITG_14194	Pra82574	Phytophthora ramorum	434	18	0.979629
PITG_14194	Pso138453	Phytophthora sojae	741	289	0.352109
PITG_14194	Pso138458	Phytophthora sojae	248	204	0.464592
PITG_14194	Pso137820	Phytophthora sojae	432	20	0.662769
PITG_14194	Pso138469	Phytophthora sojae	251	201	0.690985
PITG_14194	Pso138457	Phytophthora sojae	395	57	0.826659
PITG_14206	Pra96650	Phytophthora ramorum	518	126	0.495876
PITG_14206	Pra82571	Phytophthora ramorum	376	16	0.874667
PITG_14206	Pra82574	Phytophthora ramorum	434	42	0.899718
PITG_14206	Pso138458	Phytophthora sojae	248	144	0.456247
PITG_14206	Pso138453	Phytophthora sojae	741	349	0.545416
PITG_14206	Pso138469	Phytophthora sojae	251	141	0.657262
PITG_14206	Pso137820	Phytophthora sojae	432	40	0.787231
PITG_14206	Pso137815	Phytophthora sojae	427	35	0.812699
PITG_14215	Pra82570	Phytophthora ramorum	439	124	0.298383
PITG_14215	Pra82574	Phytophthora ramorum	434	119	0.965783
PITG_14215	Pso137819	Phytophthora sojae	470	155	0.331355
PITG_14215	Pso137820	Phytophthora sojae	432	117	0.939119
PITG_14598	Pra71277	Phytophthora ramorum	749	4	0.074242
PITG_14598	Pso108684	Phytophthora sojae	750	3	0.070865
PITG_16930	Pra75381	Phytophthora ramorum	567	139	0.175444
PITG_16930	Pso139251	Phytophthora sojae	589	117	0.164055
PITG_17257	Pra94872	Phytophthora ramorum	695	19	0.121517
PITG_17257	Pso142894	Phytophthora sojae	1139	463	0.157388
PITG_17768	Pra76021	Phytophthora ramorum	1412	1128	0.154129
PITG_17768	Pso143395	Phytophthora sojae	285	1	0.146486
PITG_17768	Pso143396	Phytophthora sojae	344	60	0.608545
PITG_18907	Pra72362	Phytophthora ramorum	349	18	0.349548
PITG_18907	Pra71818	Phytophthora ramorum	349	18	0.349548
PITG_18907	Pra72490	Phytophthora ramorum	349	18	0.349548
PITG_18907	Pra87811	Phytophthora ramorum	275	56	0.360166
PITG_18907	Pra87810	Phytophthora ramorum	147	184	0.36164
PITG_18907	Pso144380	Phytophthora sojae	171	160	0.382591
PITG_18907	Pso134353	Phytophthora sojae	294	37	0.411585
PITG_18907	Pso134354	Phytophthora sojae	343	12	0.417918
PITG_18907	Pso128784	Phytophthora sojae	345	14	0.429326
PITG_18907	Pso127156	Phytophthora sojae	333	2	0.434832
PITG_19054	Pra83395	Phytophthora ramorum	318	104	0.102816
PITG_19054	Pra51464	Phytophthora ramorum	254	168	0.797625

PITG_19054	Pra94820	Phytophthora ramorum	290	132	0.93522
PITG_19054	Pso143153	Phytophthora sojae	422	0	0.093028
PITG_19054	Pso120335	Phytophthora sojae	260	162	0.819728
PITG_19054	Pso109610	Phytophthora sojae	288	134	0.946132
PITG_20223	Pra71737	Phytophthora ramorum	309	191	0.195607
PITG_20223	Pso108341	Phytophthora sojae	312	194	0.189079
