# Supplementary material for: The CAZyome of Phytophthora spp.: A comprehensive analysis of the gene complement coding for carbohydrate-active enzymes in species of the genus Phytophthora
Source: BMC Genomics. 2010 Sep 28;11:525. doi: 10.1186/1471-2164-11-525 (PMC2997016; doi:10.1186/1471-2164-11-525)
Supplement: Additional file 5 — Phytophthora GH orthologs. Phytophthora GH orthologs as determined using the Phylogenetic Resources for the Interpretation of Genomes (PHRINGE). In most cases, more than one orthologous gene was found in each of the three Phytophthora species. Two hundred fourteen GH-coding genes had orthologs in both P. sojae and P. ramorum. Three putative GH-coding genes had an ortholog only in P. ramorum, while seven other had an ortholog in P. sojae only. The remaining 20 genes did not appear to have orthologs in either genome. [file 1471-2164-11-525-S5.PDF]

GLYCOSIDE HYDROLASES

Gene Identifier	Matching Gene	Source Organism	Length	Difference in Length	Distance Score
PITG_00219	Pra72578	Phytophthora ramorum	405	48	0.134721
PITG_00219	Pso108444	Phytophthora sojae	403	46	0.151459
PITG_00972.1	Pra72210	Phytophthora ramorum	402	53	0.10727
PITG_00972.1	Pra84285	Phytophthora ramorum	413	42	0.247106
PITG_00972.1	Pso132858	Phytophthora sojae	413	42	0.07653
PITG_00972.1	Pso138265	Phytophthora sojae	413	42	0.135099
PITG_00972.1	Pso132859	Phytophthora sojae	413	42	0.146507
PITG_00972.1	Pso109125	Phytophthora sojae	393	62	0.256807
PITG_01267	Pra76381	Phytophthora ramorum	506	33	0.126871
PITG_01267	Pso128951	Phytophthora sojae	503	30	0.130119
PITG_01269	Pra76382	Phytophthora ramorum	497	181	0.193659
PITG_01269	Pso128950	Phytophthora sojae	483	167	0.210392
PITG_01283	Pra42174	Phytophthora ramorum	394	1914	0.016879
PITG_01283	Pra71412	Phytophthora ramorum	447	1861	0.021342
PITG_01283	Pra76398	Phytophthora ramorum	1336	972	0.158706
PITG_01283	Pra71413	Phytophthora ramorum	325	1983	0.297601
PITG_01283	Pso108317	Phytophthora sojae	447	1861	0.027789
PITG_01283	Pso128935	Phytophthora sojae	1359	949	0.143452
PITG_01283	Pso108316	Phytophthora sojae	330	1978	0.262424
PITG_01391.1	Pra76490	Phytophthora ramorum	571	19	0.178526
PITG_01391.1	Pra39549	Phytophthora ramorum	52	500	0.241328
PITG_01391.1	Pso137933	Phytophthora sojae	570	18	0.188997
PITG_01395.1	Pra76490	Phytophthora ramorum	571	1	0.116938
PITG_01395.1	Pra39549	Phytophthora ramorum	52	520	0.214664
PITG_01395.1	Pso137933	Phytophthora sojae	570	2	0.119264
PITG_01396.1	Pra76491	Phytophthora ramorum	573	36	0.139732
PITG_01396.1	Pra39549	Phytophthora ramorum	52	485	0.190458
PITG_01397.1	Pra76492	Phytophthora ramorum	576	1	0.183735
PITG_01397.1	Pso137931	Phytophthora sojae	579	4	0.167337
PITG_01397.1	Pso137928	Phytophthora sojae	1868	1293	0.289852
PITG_01398.1	Pra76494	Phytophthora ramorum	584	1	0.148938
PITG_01398.1	Pra76495	Phytophthora ramorum	581	2	0.278936
PITG_01398.1	Pra39549	Phytophthora ramorum	52	531	0.296255
PITG_01398.1	Pso137930	Phytophthora sojae	587	4	0.134776

PITG_01398.1	Pso137929	Phytophthora sojae	584	1	0.296657
PITG_01399.1	Pra76495	Phytophthora ramorum	581	89	0.179161
PITG_01399.1	Pra76494	Phytophthora ramorum	584	92	0.296927
PITG_01399.1	Pso137929	Phytophthora sojae	584	92	0.179278
PITG_01430.1	Pso137886	Phytophthora sojae	579	11	0.289673
PITG_01431.1	Pra76529	Phytophthora ramorum	593	7	0.132569
PITG_01431.1	Pra39549	Phytophthora ramorum	52	534	0.250368
PITG_01431.1	Pso137885	Phytophthora sojae	596	10	0.14599
PITG_01462.1	Pra94544	Phytophthora ramorum	571	3	0.164002
PITG_01462.1	Pra39549	Phytophthora ramorum	52	522	0.179059
PITG_01462.1	Pso137835	Phytophthora sojae	575	1	0.158009
PITG_01471	Pra86970	Phytophthora ramorum	328	142	0.258491
PITG_01484.1	Pra94544	Phytophthora ramorum	571	36	0.166645
PITG_01484.1	Pra39549	Phytophthora ramorum	52	483	0.179059
PITG_01484.1	Pra83520	Phytophthora ramorum	543	8	0.293908
PITG_01484.1	Pra83519	Phytophthora ramorum	545	10	0.297374
PITG_01484.1	Pso137835	Phytophthora sojae	575	40	0.156025
PITG_01604	Pra71166	Phytophthora ramorum	493	49	0.059232
PITG_01604	Pra74011	Phytophthora ramorum	470	26	0.139448
PITG_01604	Pso108183	Phytophthora sojae	468	24	0.041694
PITG_01605	Pra74011	Phytophthora ramorum	470	15	0.11701
PITG_01605	Pra71166	Phytophthora ramorum	493	8	0.224659
PITG_01605	Pso108183	Phytophthora sojae	468	17	0.164627
PITG_01760.1	Pra73895	Phytophthora ramorum	851	171	0.19206
PITG_01760.1	Pso144622	Phytophthora sojae	1060	380	0.156588
PITG_01765.1	Pra73896	Phytophthora ramorum	815	695	0.220269
PITG_01765.1	Pso127729	Phytophthora sojae	851	731	0.234933
PITG_01855	Pra50011	Phytophthora ramorum	455	136	0.070273
PITG_01855	Pso118482	Phytophthora sojae	453	138	0.077112
PITG_02103	Pra73436	Phytophthora ramorum	808	2	0.141514
PITG_02103	Pra86694	Phytophthora ramorum	300	510	0.203609
PITG_02103	Pso144049	Phytophthora sojae	806	4	0.133545
PITG_02114	Pra71108	Phytophthora ramorum	463	0	0.050847
PITG_02114	Pso109605	Phytophthora sojae	463	0	0.044483
PITG_02266	Pra49663	Phytophthora ramorum	447	593	0.092529
PITG_02266	Pso108279	Phytophthora sojae	444	596	0.088134

PITG_02901	Pra80534	Phytophthora ramorum	280	0	0.068008
PITG_02901	Pra80529	Phytophthora ramorum	280	0	0.231225
PITG_02901	Pso136281	Phytophthora sojae	321	41	0.083316
PITG_02901	Pso136285	Phytophthora sojae	3150	2870	0.278722
PITG_03135	Pra78229	Phytophthora ramorum	792	59	0.189064
PITG_03135	Pso133713	Phytophthora sojae	797	64	0.166372
PITG_03136	Pra78230	Phytophthora ramorum	785	6	0.103204
PITG_03136	Pra78231	Phytophthora ramorum	814	23	0.256236
PITG_03136	Pso133712	Phytophthora sojae	700	91	0.118114
PITG_03136	Pso133711	Phytophthora sojae	816	25	0.231148
PITG_03140	Pra78231	Phytophthora ramorum	814	60	0.107076
PITG_03140	Pra78230	Phytophthora ramorum	785	31	0.209069
PITG_03140	Pra83174	Phytophthora ramorum	778	24	0.292177
PITG_03140	Pso133711	Phytophthora sojae	816	62	0.110472
PITG_03140	Pso133712	Phytophthora sojae	700	54	0.227936
PITG_03141	Pra78232	Phytophthora ramorum	788	361	0.151007
PITG_03141	Pso133710	Phytophthora sojae	787	362	0.145871
PITG_03213	Pra78295	Phytophthora ramorum	776	93	0.213404
PITG_03213	Pso133642	Phytophthora sojae	775	92	0.12464
PITG_03213	Pso133644	Phytophthora sojae	379	304	0.165237
PITG_03213	Pso133645	Phytophthora sojae	1020	337	0.182889
PITG_03213	Pso145415	Phytophthora sojae	581	102	0.263726
PITG_03411	Pra76113	Phytophthora ramorum	765	4	0.176591
PITG_03411	Pso134170	Phytophthora sojae	890	129	0.168277
PITG_03425	Pra76099	Phytophthora ramorum	583	25	0.121153
PITG_03425	Pso134157	Phytophthora sojae	580	22	0.10969
PITG_03460.1	Pra71383	Phytophthora ramorum	455	0	0.06488
PITG_03460.1	Pso108754	Phytophthora sojae	455	0	0.060155
PITG_03465.1	Pra71382	Phytophthora ramorum	283	124	0.012174
PITG_03465.1	Pso108753	Phytophthora sojae	283	124	0.012154
PITG_03508.1	Pra81354	Phytophthora ramorum	796	306	0.193858
PITG_03508.1	Pra81355	Phytophthora ramorum	751	261	0.287589
PITG_03508.1	Pso144044	Phytophthora sojae	127	363	0.138985
PITG_03508.1	Pso134768	Phytophthora sojae	341	149	0.205543
PITG_03508.1	Pso144769	Phytophthora sojae	137	353	0.242009
PITG_03508.1	Pso144768	Phytophthora sojae	699	209	0.269629

PITG_03508.1	Pso134766	Phytophthora sojae	742	252	0.287492
PITG_03511.1	Pra81354	Phytophthora ramorum	796	1	0.112203
PITG_03511.1	Pra81355	Phytophthora ramorum	751	46	0.23075
PITG_03511.1	Pso144044	Phytophthora sojae	127	670	0.101357
PITG_03511.1	Pso144045	Phytophthora sojae	182	615	0.13236
PITG_03511.1	Pso134768	Phytophthora sojae	341	456	0.169608
PITG_03511.1	Pso144768	Phytophthora sojae	699	98	0.194227
PITG_03511.1	Pso144769	Phytophthora sojae	137	660	0.2003
PITG_03512.1	Pra81355	Phytophthora ramorum	751	103	0.162619
PITG_03512.1	Pra81354	Phytophthora ramorum	796	148	0.285545
PITG_03512.1	Pso144045	Phytophthora sojae	182	466	0.245558
PITG_03512.1	Pso144768	Phytophthora sojae	699	51	0.274343
PITG_03512.1	Pso144044	Phytophthora sojae	127	521	0.278466
PITG_03533.1	Pra81368	Phytophthora ramorum	2213	1663	0.248852
PITG_03533.1	Pra81371	Phytophthora ramorum	929	379	0.25007
PITG_03533.1	Pra81385	Phytophthora ramorum	929	379	0.276119
PITG_03533.1	Pso145612	Phytophthora sojae	666	116	0.23594
PITG_03533.1	Pso134784	Phytophthora sojae	776	226	0.265374
PITG_03533.1	Pso134785	Phytophthora sojae	932	382	0.271855
PITG_03535.1	Pra81371	Phytophthora ramorum	929	50	0.234675
PITG_03535.1	Pso145612	Phytophthora sojae	666	213	0.213025
PITG_03535.1	Pso134785	Phytophthora sojae	932	53	0.261193
PITG_03535.1	Pso134784	Phytophthora sojae	776	103	0.299172
PITG_03554.1	Pra81385	Phytophthora ramorum	929	269	0.162299
PITG_03554.1	Pra81371	Phytophthora ramorum	929	269	0.22184
PITG_03554.1	Pra81368	Phytophthora ramorum	2213	1553	0.233949
PITG_03554.1	Pso145612	Phytophthora sojae	666	6	0.258136
PITG_03554.1	Pso134784	Phytophthora sojae	776	116	0.274803
PITG_03554.1	Pso134785	Phytophthora sojae	932	272	0.284213
PITG_03750.1	Pra75292	Phytophthora ramorum	757	158	0.137608
PITG_03750.1	Pso136482	Phytophthora sojae	757	158	0.130663
PITG_04124	Pra74256	Phytophthora ramorum	455	189	0.244103
PITG_04124	Pso131140	Phytophthora sojae	391	125	0.247816
PITG_04135	Pra74264	Phytophthora ramorum	289	1	0.185324
PITG_04135	Pra86478	Phytophthora ramorum	168	120	0.256172
PITG_04135	Pra74265	Phytophthora ramorum	289	1	0.296774

PITG_04135	Pso131083	Phytophthora sojae	289	1	0.140922
PITG_04135	Pso131089	Phytophthora sojae	103	185	0.247467
PITG_04135	Pso145618	Phytophthora sojae	103	185	0.247467
PITG_04135	Pso145591	Phytophthora sojae	182	106	0.29977
PITG_04141	Pra74267	Phytophthora ramorum	296	4	0.228543
PITG_04141	Pra74266	Phytophthora ramorum	245	55	0.265627
PITG_04141	Pso145618	Phytophthora sojae	103	197	0.161759
PITG_04141	Pso131089	Phytophthora sojae	103	197	0.161759
PITG_04141	Pso131091	Phytophthora sojae	113	187	0.163374
PITG_04141	Pso145591	Phytophthora sojae	182	118	0.172768
PITG_04141	Pso131095	Phytophthora sojae	300	0	0.19174
PITG_04158	Pra74267	Phytophthora ramorum	296	3	0.165522
PITG_04158	Pra74266	Phytophthora ramorum	245	54	0.253053
PITG_04158	Pso131091	Phytophthora sojae	113	186	0.182715
PITG_04158	Pso131095	Phytophthora sojae	300	1	0.228176
PITG_04158	Pso145591	Phytophthora sojae	182	117	0.25354
PITG_04207	Pra52439	Phytophthora ramorum	528	189	0.199015
PITG_04207	Pra81639	Phytophthora ramorum	542	203	0.288411
PITG_04207	Pra81640	Phytophthora ramorum	542	203	0.29357
PITG_04207	Pso108522	Phytophthora sojae	541	202	0.178173
PITG_04255	Pra52439	Phytophthora ramorum	528	13	0.180463
PITG_04255	Pra81639	Phytophthora ramorum	542	1	0.23846
PITG_04255	Pra81640	Phytophthora ramorum	542	1	0.239281
PITG_04255	Pso108522	Phytophthora sojae	541	0	0.156363
PITG_04255	Pso130485	Phytophthora sojae	455	86	0.267273
PITG_04255	Pso130491	Phytophthora sojae	532	9	0.279045
PITG_04273	Pra74319	Phytophthora ramorum	392	179	0.220757
PITG_04273	Pso131015	Phytophthora sojae	406	193	0.134684
PITG_05011	Pra73539	Phytophthora ramorum	543	0	0.122861
PITG_05011	Pso136107	Phytophthora sojae	543	0	0.115012
PITG_05066	Pra79077	Phytophthora ramorum	789	85	0.065818
PITG_05066	Pra79076	Phytophthora ramorum	789	85	0.065818
PITG_05066	Pra78296	Phytophthora ramorum	788	84	0.14614
PITG_05066	Pra86922	Phytophthora ramorum	788	84	0.14614
PITG_05066	Pra78297	Phytophthora ramorum	788	84	0.14614
PITG_05066	Pso133932	Phytophthora sojae	316	388	0.255061

PITG_05097.1	Pra82838	Phytophthora ramorum	696	42	0.165738
PITG_05097.1	Pso132256	Phytophthora sojae	696	42	0.149995
PITG_05097.1	Pso132248	Phytophthora sojae	530	124	0.17475
PITG_05373.1	Pra46388	Phytophthora ramorum	165	222	0.049591
PITG_05373.1	Pra72089	Phytophthora ramorum	393	6	0.098987
PITG_05373.1	Pso108408	Phytophthora sojae	389	2	0.070303
PITG_05705.1	Pra84162	Phytophthora ramorum	210	715	0.202166
PITG_05705.1	Pra86539	Phytophthora ramorum	268	657	0.220652
PITG_05939.1	Pra71963	Phytophthora ramorum	248	0	0.123575
PITG_05939.1	Pso109590	Phytophthora sojae	249	1	0.086251
PITG_06563	Pra78296	Phytophthora ramorum	788	351	0.230542
PITG_06563	Pra86922	Phytophthora ramorum	788	351	0.230542
PITG_06563	Pra78297	Phytophthora ramorum	788	351	0.230542
PITG_06563	Pra79076	Phytophthora ramorum	789	352	0.265551
PITG_06563	Pra79077	Phytophthora ramorum	789	352	0.265551
PITG_06756	Pra71652	Phytophthora ramorum	336	22	0.169051
PITG_06756	Pso109322	Phytophthora sojae	338	24	0.149727
PITG_06788	Pra72061	Phytophthora ramorum	488	185	0.214107
PITG_06788	Pra53490	Phytophthora ramorum	452	221	0.265842
PITG_06788	Pso108891	Phytophthora sojae	539	134	0.21738
PITG_06808	Pra82922	Phytophthora ramorum	863	223	0.274246
PITG_06808	Pso135687	Phytophthora sojae	646	6	0.245285
PITG_06820	Pra82934	Phytophthora ramorum	750	49	0.197633
PITG_06820	Pso108895	Phytophthora sojae	577	124	0.097468
PITG_06864.1	Pra79858	Phytophthora ramorum	896	132	0.274081
PITG_06864.1	Pso135747	Phytophthora sojae	1331	567	0.268456
PITG_06962	Pra79939	Phytophthora ramorum	436	30	0.28365
PITG_06962	Pso138787	Phytophthora sojae	427	21	0.254504
PITG_07365	Pra53914	Phytophthora ramorum	452	118	0.171134
PITG_07365	Pso117121	Phytophthora sojae	449	121	0.173005
PITG_08000	Pra81296	Phytophthora ramorum	680	0	0.150849
PITG_08000	Pso138214	Phytophthora sojae	162	518	0.210475
PITG_08001	Pra81297	Phytophthora ramorum	678	4	0.184365
PITG_08001	Pra81300	Phytophthora ramorum	676	2	0.191556
PITG_08001	Pso138213	Phytophthora sojae	677	3	0.183977
PITG_08002	Pso138217	Phytophthora sojae	684	98	0.175869

PITG_08004	Pra81297	Phytophthora ramorum	678	39	0.27397
PITG_08004	Pra81300	Phytophthora ramorum	676	37	0.278842
PITG_08004	Pso138213	Phytophthora sojae	677	38	0.268814
PITG_08027	Pra81321	Phytophthora ramorum	791	81	0.199088
PITG_08027	Pso138254	Phytophthora sojae	777	67	0.206256
PITG_08191	Pso130499	Phytophthora sojae	540	170	0.174937
PITG_08191	Pso135397	Phytophthora sojae	416	46	0.23559
PITG_08435	Pra93781	Phytophthora ramorum	546	8	0.158292
PITG_08435	Pso158003	Phytophthora sojae	510	28	0.192129
PITG_08468.1	Pra72095	Phytophthora ramorum	335	0	0.125087
PITG_08468.1	Pra72356	Phytophthora ramorum	335	0	0.128294
PITG_08468.1	Pra83258	Phytophthora ramorum	335	0	0.152797
PITG_08468.1	Pso109219	Phytophthora sojae	335	0	0.088538
PITG_08610	Pra83874	Phytophthora ramorum	584	5	0.10206
PITG_08610	Pso132180	Phytophthora sojae	587	8	0.199426
PITG_08611	Pra87384	Phytophthora ramorum	662	79	0.231867
PITG_08611	Pso145267	Phytophthora sojae	124	459	0.275639
PITG_08612	Pso145267	Phytophthora sojae	124	451	0.140538
PITG_08612	Pso132177	Phytophthora sojae	592	17	0.189047
PITG_08612	Pso129514	Phytophthora sojae	132	443	0.235897
PITG_08613	Pra87384	Phytophthora ramorum	662	79	0.102797
PITG_08613	Pra83876	Phytophthora ramorum	457	126	0.197363
PITG_08613	Pso145267	Phytophthora sojae	124	459	0.218914
PITG_08613	Pso129514	Phytophthora sojae	132	451	0.239781
PITG_08842.1	Pra72388	Phytophthora ramorum	356	133	0.22397
PITG_08842.1	Pra86733	Phytophthora ramorum	356	133	0.228398
PITG_08842.1	Pra46413	Phytophthora ramorum	191	32	0.277979
PITG_08842.1	Pso109744	Phytophthora sojae	356	133	0.19439
PITG_08842.1	Pso109585	Phytophthora sojae	355	132	0.224919
PITG_08842.1	Pso108331	Phytophthora sojae	356	133	0.236926
PITG_08843.1	Pra72388	Phytophthora ramorum	356	1	0.198629
PITG_08843.1	Pra86733	Phytophthora ramorum	356	1	0.205258
PITG_08843.1	Pra46413	Phytophthora ramorum	191	164	0.268897
PITG_08843.1	Pso109744	Phytophthora sojae	356	1	0.189749
PITG_08843.1	Pso108331	Phytophthora sojae	356	1	0.215127
PITG_08843.1	Pso109585	Phytophthora sojae	355	0	0.217615

PITG_08843.1	Pso126876	Phytophthora sojae	47	308	0.280009
PITG_08843.1	Pso109686	Phytophthora sojae	356	1	0.294152
PITG_08844.1	Pra72388	Phytophthora ramorum	356	9	0.248768
PITG_08844.1	Pra86733	Phytophthora ramorum	356	9	0.25589
PITG_08844.1	Pso109744	Phytophthora sojae	356	9	0.239595
PITG_08844.1	Pso108331	Phytophthora sojae	356	9	0.267289
PITG_08844.1	Pso109585	Phytophthora sojae	355	10	0.273666
PITG_08844.1	Pso126876	Phytophthora sojae	47	318	0.280009
PITG_08845.1	Pra72388	Phytophthora ramorum	356	17	0.208381
PITG_08845.1	Pra86733	Phytophthora ramorum	356	17	0.211532
PITG_08845.1	Pra46413	Phytophthora ramorum	191	148	0.277979
PITG_08845.1	Pso109744	Phytophthora sojae	356	17	0.195326
PITG_08845.1	Pso108331	Phytophthora sojae	356	17	0.221974
PITG_08845.1	Pso109585	Phytophthora sojae	355	16	0.228485
PITG_08845.1	Pso109686	Phytophthora sojae	356	17	0.291192
PITG_08846.1	Pra86733	Phytophthora ramorum	356	0	0.112449
PITG_08846.1	Pra72388	Phytophthora ramorum	356	0	0.118739
PITG_08846.1	Pra46413	Phytophthora ramorum	191	165	0.204297
PITG_08846.1	Pra86732	Phytophthora ramorum	355	1	0.294087
PITG_08846.1	Pso108331	Phytophthora sojae	356	0	0.106522
PITG_08846.1	Pso109744	Phytophthora sojae	356	0	0.133506
PITG_08846.1	Pso109585	Phytophthora sojae	355	1	0.136956
PITG_08846.1	Pso109706	Phytophthora sojae	356	0	0.208037
PITG_08846.1	Pso125904	Phytophthora sojae	131	225	0.210549
PITG_08850.1	Pra86732	Phytophthora ramorum	355	148	0.115879
PITG_08850.1	Pso126876	Phytophthora sojae	47	456	0.066261
PITG_08866.1	Pra46413	Phytophthora ramorum	191	157	0.224675
PITG_08866.1	Pra86733	Phytophthora ramorum	356	8	0.249242
PITG_08866.1	Pra72388	Phytophthora ramorum	356	8	0.256472
PITG_08866.1	Pso109686	Phytophthora sojae	356	8	0.159538
PITG_08866.1	Pso109706	Phytophthora sojae	356	8	0.211344
PITG_08866.1	Pso108331	Phytophthora sojae	356	8	0.245741
PITG_08866.1	Pso109585	Phytophthora sojae	355	7	0.251407
PITG_08866.1	Pso109744	Phytophthora sojae	356	8	0.25491
PITG_08867.1	Pra46413	Phytophthora ramorum	191	253	0.059134
PITG_08867.1	Pra86733	Phytophthora ramorum	356	88	0.234924

PITG_08867.1	Pra72388	Phytophthora ramorum	356	88	0.253422
PITG_08867.1	Pso109706	Phytophthora sojae	356	88	0.066836
PITG_08867.1	Pso125904	Phytophthora sojae	131	313	0.082502
PITG_08867.1	Pso109686	Phytophthora sojae	356	88	0.126473
PITG_08867.1	Pso108331	Phytophthora sojae	356	88	0.221434
PITG_08867.1	Pso109744	Phytophthora sojae	356	88	0.247606
PITG_08869.1	Pra46413	Phytophthora ramorum	191	165	0.059134
PITG_08869.1	Pra71551	Phytophthora ramorum	354	0	0.095868
PITG_08869.1	Pra71553	Phytophthora ramorum	354	0	0.105016
PITG_08869.1	Pra77770	Phytophthora ramorum	239	115	0.202945
PITG_08869.1	Pra77768	Phytophthora ramorum	241	113	0.213908
PITG_08869.1	Pra86733	Phytophthora ramorum	356	0	0.230034
PITG_08869.1	Pra72388	Phytophthora ramorum	356	0	0.248356
PITG_08869.1	Pso155220	Phytophthora sojae	88	266	0.0568
PITG_08869.1	Pso109706	Phytophthora sojae	356	0	0.063474
PITG_08869.1	Pso125904	Phytophthora sojae	131	225	0.072756
PITG_08869.1	Pso109686	Phytophthora sojae	356	0	0.122572
PITG_08869.1	Pso108331	Phytophthora sojae	356	0	0.217601
PITG_08869.1	Pso109744	Phytophthora sojae	356	0	0.242512
PITG_08998	Pra80684	Phytophthora ramorum	372	3	0.194599
PITG_08998	Pso131561	Phytophthora sojae	375	6	0.168616
PITG_08999	Pra80685	Phytophthora ramorum	470	17	0.25768
PITG_08999	Pso131560	Phytophthora sojae	474	21	0.28013
PITG_09003.1	Pra50225	Phytophthora ramorum	366	355	0.125095
PITG_09003.1	Pra41436	Phytophthora ramorum	342	379	0.13123
PITG_09003.1	Pso109723	Phytophthora sojae	367	354	0.123203
PITG_09101	Pso130499	Phytophthora sojae	540	99	0.274222
PITG_09377	Pra40131	Phytophthora ramorum	690	30	0.176065
PITG_09377	Pso141218	Phytophthora sojae	728	8	0.21194
PITG_09760.1	Pra75750	Phytophthora ramorum	1632	142	0.196562
PITG_09791.1	Pra75725	Phytophthora ramorum	504	12	0.120288
PITG_09791.1	Pso144153	Phytophthora sojae	497	5	0.123742
PITG_09872	Pra76972	Phytophthora ramorum	633	7	0.13935
PITG_09872	Pso132075	Phytophthora sojae	640	0	0.167509
PITG_09906	Pso131883	Phytophthora sojae	362	37	0.225765
PITG_09907	Pra76944	Phytophthora ramorum	364	79	0.210183

PITG_09910	Pra76943	Phytophthora ramorum	360	1	0.208123
PITG_09910	Pso131879	Phytophthora sojae	364	5	0.238653
PITG_10027.1	Pra80249	Phytophthora ramorum	805	120	0.232884
PITG_10027.1	Pso131783	Phytophthora sojae	783	98	0.163351
PITG_10028	Pra80248	Phytophthora ramorum	534	0	0.090711
PITG_10028	Pso131782	Phytophthora sojae	515	19	0.124892
PITG_10050.1	Pra95635	Phytophthora ramorum	964	227	0.178696
PITG_10050.1	Pso131764	Phytophthora sojae	997	260	0.153775
PITG_10149	Pra45104	Phytophthora ramorum	451	54	0.298624
PITG_10149	Pso123276	Phytophthora sojae	440	65	0.277947
PITG_10204	Pra71345	Phytophthora ramorum	472	7	0.127543
PITG_10204	Pso109096	Phytophthora sojae	457	8	0.117196
PITG_10255	Pra72132	Phytophthora ramorum	382	2	0.251369
PITG_10255	Pra54527	Phytophthora ramorum	330	50	0.257409
PITG_10255	Pra41820	Phytophthora ramorum	343	37	0.28524
PITG_10255	Pra72133	Phytophthora ramorum	383	3	0.292376
PITG_10255	Pso108849	Phytophthora sojae	406	26	0.196777
PITG_10255	Pso135149	Phytophthora sojae	406	26	0.196777
PITG_10255	Pso135147	Phytophthora sojae	406	26	0.196777
PITG_10255	Pso135151	Phytophthora sojae	410	30	0.229178
PITG_10255	Pso145625	Phytophthora sojae	366	14	0.24231
PITG_10290.1	Pra84285	Phytophthora ramorum	413	2	0.151401
PITG_10290.1	Pra72210	Phytophthora ramorum	402	9	0.273394
PITG_10290.1	Pso109125	Phytophthora sojae	393	18	0.139792
PITG_10290.1	Pso132859	Phytophthora sojae	413	2	0.250673
PITG_10290.1	Pso138265	Phytophthora sojae	413	2	0.251421
PITG_10290.1	Pso132858	Phytophthora sojae	413	2	0.284551
PITG_10292.1	Pra72210	Phytophthora ramorum	402	11	0.093908
PITG_10292.1	Pra84285	Phytophthora ramorum	413	0	0.187535
PITG_10292.1	Pso132859	Phytophthora sojae	413	0	0.066344
PITG_10292.1	Pso138265	Phytophthora sojae	413	0	0.077404
PITG_10292.1	Pso132858	Phytophthora sojae	413	0	0.085549
PITG_10292.1	Pso109125	Phytophthora sojae	393	20	0.193462
PITG_10293.1	Pra72210	Phytophthora ramorum	402	53	0.10727
PITG_10293.1	Pra84285	Phytophthora ramorum	413	42	0.247106
PITG_10293.1	Pso132858	Phytophthora sojae	413	42	0.07653

PITG_10293.1	Pso138265	Phytophthora sojae	413	42	0.135099
PITG_10293.1	Pso132859	Phytophthora sojae	413	42	0.146507
PITG_10293.1	Pso109125	Phytophthora sojae	393	62	0.256807
PITG_10399	Pra82083	Phytophthora ramorum	548	57	0.103187
PITG_10399	Pso138342	Phytophthora sojae	553	62	0.10741
PITG_10714	Pra72059	Phytophthora ramorum	461	181	0.259441
PITG_10846	Pra76244	Phytophthora ramorum	1228	78	0.236224
PITG_10846	Pso141463	Phytophthora sojae	1243	93	0.226793
PITG_10855.1	Pra86733	Phytophthora ramorum	356	98	0.261557
PITG_10855.1	Pra72388	Phytophthora ramorum	356	98	0.286129
PITG_10855.1	Pso109686	Phytophthora sojae	356	98	0.17259
PITG_10855.1	Pso109706	Phytophthora sojae	356	98	0.245238
PITG_10855.1	Pso108331	Phytophthora sojae	356	98	0.272966
PITG_10855.1	Pso109744	Phytophthora sojae	356	98	0.291168
PITG_10880	Pso109521	Phytophthora sojae	443	225	0.280825
PITG_10999.1	Pra74498	Phytophthora ramorum	755	9	0.165482
PITG_10999.1	Pso142277	Phytophthora sojae	751	5	0.172898
PITG_11050.1	Pra74531	Phytophthora ramorum	796	16	0.141715
PITG_11050.1	Pra74534	Phytophthora ramorum	796	16	0.142927
PITG_11050.1	Pso136653	Phytophthora sojae	787	7	0.172183
PITG_11050.1	Pso136654	Phytophthora sojae	787	7	0.172183
PITG_11050.1	Pso133043	Phytophthora sojae	219	561	0.210893
PITG_11050.1	Pso144920	Phytophthora sojae	259	521	0.232815
PITG_11052.1	Pra74531	Phytophthora ramorum	796	101	0.104516
PITG_11052.1	Pra74534	Phytophthora ramorum	796	101	0.105874
PITG_11052.1	Pso136653	Phytophthora sojae	787	92	0.119312
PITG_11052.1	Pso136654	Phytophthora sojae	787	92	0.119312
PITG_11052.1	Pso133043	Phytophthora sojae	219	476	0.210893
PITG_11052.1	Pso142344	Phytophthora sojae	672	23	0.228699
PITG_11052.1	Pso144920	Phytophthora sojae	259	436	0.232815
PITG_11112	Pra73317	Phytophthora ramorum	1021	692	0.183959
PITG_11112	Pso108542	Phytophthora sojae	969	640	0.182846
PITG_11293.1	Pra47831	Phytophthora ramorum	84	276	0.086318
PITG_11293.1	Pra47833	Phytophthora ramorum	352	8	0.164904
PITG_11293.1	Pso115499	Phytophthora sojae	341	19	0.190478
PITG_11293.1	Pso109711	Phytophthora sojae	369	9	0.195981

PITG_11294.1	Pra47832	Phytophthora ramorum	81	297	0.105314
PITG_11294.1	Pso133583	Phytophthora sojae	221	157	0.284454
PITG_11295.1	Pra47833	Phytophthora ramorum	352	10	0.148287
PITG_11295.1	Pra47831	Phytophthora ramorum	84	278	0.165682
PITG_11295.1	Pso115499	Phytophthora sojae	341	21	0.116796
PITG_11295.1	Pso109711	Phytophthora sojae	369	7	0.150061
PITG_11537	Pra53914	Phytophthora ramorum	452	125	0.074993
PITG_11537	Pso117121	Phytophthora sojae	449	128	0.078764
PITG_11623.1	Pra73380	Phytophthora ramorum	354	34	0.086572
PITG_11623.1	Pra72334	Phytophthora ramorum	354	34	0.256038
PITG_11623.1	Pso126091	Phytophthora sojae	96	224	0.021122
PITG_11623.1	Pso108554	Phytophthora sojae	341	21	0.093327
PITG_11623.1	Pso108553	Phytophthora sojae	226	94	0.127569
PITG_11632.1	Pra83050	Phytophthora ramorum	402	31	0.192219
PITG_11632.1	Pso127158	Phytophthora sojae	630	259	0.255555
PITG_11712	Pra73317	Phytophthora ramorum	1021	2	0.144316
PITG_11712	Pso108542	Phytophthora sojae	969	54	0.126554
PITG_11993	Pra78128	Phytophthora ramorum	538	1	0.17611
PITG_11993	Pso144753	Phytophthora sojae	541	2	0.152345
PITG_12344	Pra84834	Phytophthora ramorum	409	92	0.278602
PITG_12785	Pra84527	Phytophthora ramorum	244	0	0.285783
PITG_12785	Pra84525	Phytophthora ramorum	244	0	0.288394
PITG_12785	Pra85786	Phytophthora ramorum	244	0	0.290454
PITG_12785	Pso138868	Phytophthora sojae	244	0	0.26302
PITG_12785	Pso138872	Phytophthora sojae	244	0	0.275919
PITG_12819.1	Pra86419	Phytophthora ramorum	333	0	0.130609
PITG_12819.1	Pra74991	Phytophthora ramorum	333	0	0.130609
PITG_12819.1	Pso143293	Phytophthora sojae	370	37	0.080285
PITG_13052.1	Pra76665	Phytophthora ramorum	924	635	0.216199
PITG_13052.1	Pso140623	Phytophthora sojae	998	709	0.208493
PITG_13322.1	Pra85960	Phytophthora ramorum	796	2	0.159572
PITG_13322.1	Pra83918	Phytophthora ramorum	442	352	0.226282
PITG_13322.1	Pra83917	Phytophthora ramorum	298	496	0.276847
PITG_13322.1	Pso137093	Phytophthora sojae	795	1	0.138858
PITG_13567.1	Pra84172	Phytophthora ramorum	1124	224	0.230154
PITG_13567.1	Pso144699	Phytophthora sojae	826	74	0.256299

PITG_13569.1	Pra84172	Phytophthora ramorum	1124	319	0.254076
PITG_13569.1	Pso144699	Phytophthora sojae	826	21	0.276708
PITG_13571.1	Pra84172	Phytophthora ramorum	1124	341	0.229021
PITG_13571.1	Pso144699	Phytophthora sojae	826	43	0.266198
PITG_13867	Pra76755	Phytophthora ramorum	1089	856	0.229075
PITG_13867	Pso130345	Phytophthora sojae	1090	857	0.165741
PITG_13868	Pra76755	Phytophthora ramorum	1089	280	0.137532
PITG_13868	Pra76757	Phytophthora ramorum	876	67	0.22219
PITG_13868	Pso130345	Phytophthora sojae	1090	281	0.129409
PITG_13868	Pso130344	Phytophthora sojae	910	101	0.175804
PITG_13873	Pra76757	Phytophthora ramorum	876	32	0.273574
PITG_13873	Pra76755	Phytophthora ramorum	1089	245	0.285594
PITG_13873	Pso130344	Phytophthora sojae	910	66	0.268043
PITG_13873	Pso130345	Phytophthora sojae	1090	246	0.284987
PITG_14060	Pso140301	Phytophthora sojae	247	20	0.212454
PITG_14124	Pra83728	Phytophthora ramorum	495	186	0.147967
PITG_14124	Pso138391	Phytophthora sojae	498	189	0.133113
PITG_14138	Pra83722	Phytophthora ramorum	508	162	0.215636
PITG_14138	Pra83721	Phytophthora ramorum	980	634	0.242684
PITG_14138	Pso157826	Phytophthora sojae	1749	1403	0.207453
PITG_14138	Pso138398	Phytophthora sojae	501	155	0.236547
PITG_14138	Pso157827	Phytophthora sojae	501	155	0.240946
PITG_14138	Pso138399	Phytophthora sojae	987	641	0.246658
PITG_14139	Pra83721	Phytophthora ramorum	980	667	0.224751
PITG_14139	Pra83722	Phytophthora ramorum	508	195	0.243604
PITG_14139	Pso138398	Phytophthora sojae	501	188	0.205243
PITG_14139	Pso157827	Phytophthora sojae	501	188	0.216389
PITG_14139	Pso138399	Phytophthora sojae	987	674	0.243651
PITG_14139	Pso157826	Phytophthora sojae	1749	1436	0.25655
PITG_14140	Pra83721	Phytophthora ramorum	980	554	0.240235
PITG_14140	Pra83722	Phytophthora ramorum	508	82	0.261452
PITG_14140	Pso138398	Phytophthora sojae	501	75	0.241094
PITG_14140	Pso138399	Phytophthora sojae	987	561	0.261151
PITG_14140	Pso157827	Phytophthora sojae	501	75	0.267261
PITG_14141	Pra83721	Phytophthora ramorum	980	479	0.1477
PITG_14141	Pso157827	Phytophthora sojae	501	0	0.123788

PITG_14141	Pso142342	Phytophthora sojae	482	19	0.178346
PITG_14141	Pso138399	Phytophthora sojae	987	486	0.272505
PITG_14141	Pso138398	Phytophthora sojae	501	0	0.277953
PITG_14173.1	Pra83712	Phytophthora ramorum	596	228	0.091591
PITG_14173.1	Pra87159	Phytophthora ramorum	501	323	0.16613
PITG_14173.1	Pso138216	Phytophthora sojae	817	7	0.134654
PITG_14237	Pra82584	Phytophthora ramorum	621	3	0.102047
PITG_14237	Pra82583	Phytophthora ramorum	620	2	0.12805
PITG_14237	Pra82582	Phytophthora ramorum	664	46	0.250351
PITG_14237	Pso137802	Phytophthora sojae	621	3	0.108085
PITG_14237	Pso137803	Phytophthora sojae	620	2	0.145416
PITG_14237	Pso143222	Phytophthora sojae	322	296	0.23686
PITG_14238	Pra82582	Phytophthora ramorum	664	3	0.190758
PITG_14238	Pso137804	Phytophthora sojae	640	27	0.247213
PITG_14243	Pra82584	Phytophthora ramorum	621	133	0.200754
PITG_14243	Pra82583	Phytophthora ramorum	620	132	0.205423
PITG_14243	Pso137802	Phytophthora sojae	621	133	0.207239
PITG_14243	Pso137803	Phytophthora sojae	620	132	0.210901
PITG_14243	Pso143222	Phytophthora sojae	322	166	0.278397
PITG_15478	Pso133642	Phytophthora sojae	775	538	0.193607
PITG_15478	Pso133644	Phytophthora sojae	379	142	0.193607
PITG_15640.1	Pra71274	Phytophthora ramorum	298	0	0.119971
PITG_15790.1	Pra71121	Phytophthora ramorum	476	0	0.071682
PITG_15790.1	Pso108504	Phytophthora sojae	476	0	0.066712
PITG_15791.1	Pra73653	Phytophthora ramorum	476	0	0.106956
PITG_15791.1	Pso108505	Phytophthora sojae	478	2	0.115627
PITG_15905	Pso131903	Phytophthora sojae	780	36	0.089198
PITG_15905	Pso145005	Phytophthora sojae	190	554	0.267337
PITG_15905	Pso145416	Phytophthora sojae	314	430	0.271421
PITG_15980	Pra87481	Phytophthora ramorum	172	272	0.094187
PITG_15980	Pra81925	Phytophthora ramorum	444	0	0.100004
PITG_15980	Pra72941	Phytophthora ramorum	444	0	0.119739
PITG_15980	Pra72942	Phytophthora ramorum	713	269	0.143901
PITG_15980	Pra72940	Phytophthora ramorum	303	141	0.186504
PITG_15980	Pso128780	Phytophthora sojae	713	269	0.118894
PITG_15980	Pso128781	Phytophthora sojae	444	0	0.12

PITG_15980	Pso128802	Phytophthora sojae	408	36	0.279735
PITG_16487	Pra79038	Phytophthora ramorum	644	3	0.133882
PITG_16487	Pso135932	Phytophthora sojae	615	26	0.165151
PITG_16558	Pra79076	Phytophthora ramorum	789	56	0.066673
PITG_16558	Pra79077	Phytophthora ramorum	789	56	0.066673
PITG_16558	Pra78297	Phytophthora ramorum	788	55	0.147565
PITG_16558	Pra78296	Phytophthora ramorum	788	55	0.147565
PITG_16558	Pra86922	Phytophthora ramorum	788	55	0.147565
PITG_16558	Pso133932	Phytophthora sojae	316	417	0.25838
PITG_16822	Pra72134	Phytophthora ramorum	362	27	0.109367
PITG_16822	Pra41921	Phytophthora ramorum	125	210	0.158772
PITG_16822	Pra54493	Phytophthora ramorum	364	29	0.200927
PITG_16822	Pra83564	Phytophthora ramorum	363	28	0.204185
PITG_16822	Pra83565	Phytophthora ramorum	363	28	0.211446
PITG_16822	Pso135143	Phytophthora sojae	323	12	0.133177
PITG_16822	Pso116769	Phytophthora sojae	369	34	0.217338
PITG_16822	Pso109634	Phytophthora sojae	305	30	0.280986
PITG_16824	Pra72134	Phytophthora ramorum	362	1	0.113655
PITG_16824	Pra41921	Phytophthora ramorum	125	236	0.148715
PITG_16824	Pra83564	Phytophthora ramorum	363	2	0.224931
PITG_16824	Pra54493	Phytophthora ramorum	364	3	0.228041
PITG_16824	Pra83565	Phytophthora ramorum	363	2	0.231873
PITG_16824	Pso135143	Phytophthora sojae	323	38	0.10023
PITG_16824	Pso135142	Phytophthora sojae	184	177	0.146305
PITG_16824	Pso116769	Phytophthora sojae	369	8	0.244187
PITG_16824	Pso109634	Phytophthora sojae	305	56	0.294793
PITG_16825	Pra72134	Phytophthora ramorum	362	40	0.117407
PITG_16825	Pra41921	Phytophthora ramorum	125	197	0.191505
PITG_16825	Pra83564	Phytophthora ramorum	363	41	0.217555
PITG_16825	Pra54493	Phytophthora ramorum	364	42	0.221676
PITG_16825	Pra83565	Phytophthora ramorum	363	41	0.225142
PITG_16825	Pso135143	Phytophthora sojae	323	1	0.129728
PITG_16825	Pso116769	Phytophthora sojae	369	47	0.22211
PITG_16985	Pra72081	Phytophthora ramorum	241	20	0.266768
PITG_16985	Pso109280	Phytophthora sojae	241	20	0.292829
PITG_16991	Pra83139	Phytophthora ramorum	243	1	0.201868

PITG_16991	Pso140300	Phytophthora sojae	244	0	0.227348
PITG_16991	Pso126103	Phytophthora sojae	133	111	0.286823
PITG_16992	Pso140301	Phytophthora sojae	247	7	0.281448
PITG_17045.1	Pra72334	Phytophthora ramorum	354	190	0.242413
PITG_17045.1	Pso108553	Phytophthora sojae	226	318	0.261187
PITG_17049.1	Pra40732	Phytophthora ramorum	65	1484	0.100031
PITG_17049.1	Pra85408	Phytophthora ramorum	354	1195	0.126805
PITG_17049.1	Pra43284	Phytophthora ramorum	284	1265	0.127432
PITG_17049.1	Pra71933	Phytophthora ramorum	354	1195	0.17425
PITG_17049.1	Pra40722	Phytophthora ramorum	65	1484	0.196357
PITG_17049.1	Pso109532	Phytophthora sojae	280	1269	0.108563
PITG_17049.1	Pso109286	Phytophthora sojae	1265	284	0.199288
PITG_17049.1	Pso143060	Phytophthora sojae	505	1044	0.226998
PITG_17049.1	Pso143049	Phytophthora sojae	955	594	0.228785
PITG_17049.1	Pso139463	Phytophthora sojae	1385	164	0.291002
PITG_17054	Pra85409	Phytophthora ramorum	1362	1002	0.26413
PITG_17054	Pra83134	Phytophthora ramorum	357	3	0.274036
PITG_17054	Pra84765	Phytophthora ramorum	352	8	0.277844
PITG_17054	Pra83132	Phytophthora ramorum	357	3	0.289732
PITG_17054	Pso140369	Phytophthora sojae	384	24	0.175571
PITG_17054	Pso140368	Phytophthora sojae	357	3	0.240108
PITG_17055	Pra85409	Phytophthora ramorum	1362	977	0.271224
PITG_17055	Pra83132	Phytophthora ramorum	357	28	0.294762
PITG_17055	Pra83134	Phytophthora ramorum	357	28	0.295416
PITG_17055	Pso140368	Phytophthora sojae	357	28	0.166685
PITG_17055	Pso140369	Phytophthora sojae	384	1	0.200264
PITG_17496	Pra81632	Phytophthora ramorum	509	228	0.17937
PITG_17496	Pra81633	Phytophthora ramorum	557	276	0.184335
PITG_17496	Pso130504	Phytophthora sojae	563	282	0.133805
PITG_17496	Pso130505	Phytophthora sojae	437	156	0.133805
PITG_17497	Pra81632	Phytophthora ramorum	509	16	0.294607
PITG_17497	Pso130505	Phytophthora sojae	437	88	0.233056
PITG_17497	Pso130504	Phytophthora sojae	563	38	0.27956
PITG_17500	Pra51732	Phytophthora ramorum	492	56	0.165707
PITG_17500	Pso130502	Phytophthora sojae	549	1	0.223137
PITG_17501	Pso130499	Phytophthora sojae	540	0	0.14354

PITG_17501	Pso135397	Phytophthora sojae	416	124	0.18852
PITG_17506	Pra81642	Phytophthora ramorum	591	307	0.155611
PITG_17506	Pso130494	Phytophthora sojae	595	311	0.184796
PITG_17507	Pra51749	Phytophthora ramorum	538	21	0.160109
PITG_17507	Pra51757	Phytophthora ramorum	538	21	0.160109
PITG_17507	Pso130493	Phytophthora sojae	623	106	0.134675
PITG_17508	Pso130492	Phytophthora sojae	542	42	0.11821
PITG_17509	Pra81639	Phytophthora ramorum	542	315	0.129706
PITG_17509	Pra81640	Phytophthora ramorum	542	315	0.133573
PITG_17509	Pra52439	Phytophthora ramorum	528	301	0.172561
PITG_17509	Pso108522	Phytophthora sojae	541	314	0.148189
PITG_17509	Pso130485	Phytophthora sojae	455	228	0.166359
PITG_17509	Pso130496	Phytophthora sojae	510	283	0.212766
PITG_17509	Pso130491	Phytophthora sojae	532	305	0.270638
PITG_17546	Pra83174	Phytophthora ramorum	778	94	0.124139
PITG_17546	Pra84532	Phytophthora ramorum	507	177	0.134957
PITG_17546	Pra78230	Phytophthora ramorum	785	101	0.288407
PITG_17546	Pso143984	Phytophthora sojae	709	25	0.154256
PITG_17546	Pso145641	Phytophthora sojae	327	357	0.172195
PITG_17546	Pso142760	Phytophthora sojae	781	97	0.203295
PITG_17546	Pso133712	Phytophthora sojae	700	16	0.292225
PITG_17592	Pra54464	Phytophthora ramorum	68	824	0.149212
PITG_17592	Pra84565	Phytophthora ramorum	898	6	0.207163
PITG_17592	Pso136691	Phytophthora sojae	906	14	0.178876
PITG_17899	Pra40980	Phytophthora ramorum	332	111	0.144064
PITG_17899	Pra48642	Phytophthora ramorum	331	110	0.246465
PITG_17899	Pso127373	Phytophthora sojae	519	298	0.183048
PITG_17947	Pra71328	Phytophthora ramorum	246	11	0.187889
PITG_17947	Pso142416	Phytophthora sojae	264	29	0.189013
PITG_18069	Pra77835	Phytophthora ramorum	779	14	0.203143
PITG_18069	Pso137272	Phytophthora sojae	776	17	0.202836
PITG_18208.1	Pra71121	Phytophthora ramorum	476	233	0.108331
PITG_18208.1	Pso108504	Phytophthora sojae	476	233	0.110902
PITG_18209.1	Pra73653	Phytophthora ramorum	476	16	0.105517
PITG_18209.1	Pso108505	Phytophthora sojae	478	18	0.112027
PITG_18230.1	Pra73653	Phytophthora ramorum	476	66	0.115299

PITG_18230.1	Pso108505	Phytophthora sojae	478	68	0.119748
PITG_18231.1	Pra71121	Phytophthora ramorum	476	0	0.073866
PITG_18231.1	Pso108504	Phytophthora sojae	476	0	0.066491
PITG_18327	Pra75978	Phytophthora ramorum	389	25	0.201481
PITG_18327	Pso132603	Phytophthora sojae	415	1	0.190152
PITG_18332	Pra75973	Phytophthora ramorum	373	5	0.241053
PITG_18332	Pso132598	Phytophthora sojae	365	3	0.200136
PITG_18333	Pso132597	Phytophthora sojae	928	463	0.276677
PITG_18335	Pra86772	Phytophthora ramorum	487	43	0.290054
PITG_18335	Pra75965	Phytophthora ramorum	487	43	0.293166
PITG_18335	Pso132586	Phytophthora sojae	466	22	0.282328
PITG_18336	Pra75966	Phytophthora ramorum	482	62	0.172305
PITG_18336	Pso132587	Phytophthora sojae	459	39	0.181927
PITG_18337	Pra75967	Phytophthora ramorum	433	198	0.137718
PITG_18337	Pra75965	Phytophthora ramorum	487	252	0.23438
PITG_18337	Pra86772	Phytophthora ramorum	487	252	0.23438
PITG_18337	Pra75966	Phytophthora ramorum	482	247	0.285852
PITG_18337	Pso132588	Phytophthora sojae	334	99	0.15534
PITG_18337	Pso132586	Phytophthora sojae	466	231	0.245725
PITG_18338	Pra75969	Phytophthora ramorum	465	161	0.296952
PITG_19162.1	Pra71463	Phytophthora ramorum	398	26	0.107068
PITG_19162.1	Pso109447	Phytophthora sojae	393	31	0.1388
PITG_19455	Pra72132	Phytophthora ramorum	382	43	0.129842
PITG_19455	Pra54493	Phytophthora ramorum	364	25	0.235023
PITG_19455	Pra41820	Phytophthora ramorum	343	4	0.23559
PITG_19455	Pra83564	Phytophthora ramorum	363	24	0.251878
PITG_19455	Pra83565	Phytophthora ramorum	363	24	0.254617
PITG_19455	Pso145625	Phytophthora sojae	366	27	0.18242
PITG_19455	Pso108850	Phytophthora sojae	375	36	0.184148
PITG_19455	Pso108851	Phytophthora sojae	439	100	0.210685
PITG_19455	Pso109634	Phytophthora sojae	305	34	0.223696
PITG_19455	Pso116769	Phytophthora sojae	369	30	0.227277
PITG_19561.1	Pra73896	Phytophthora ramorum	815	35	0.211927
PITG_19561.1	Pso127729	Phytophthora sojae	851	71	0.208588
PITG_19619	Pra83564	Phytophthora ramorum	363	69	0.197408
PITG_19619	Pra83565	Phytophthora ramorum	363	69	0.200764

PITG_19619	Pra41820	Phytophthora ramorum	343	49	0.20505
PITG_19619	Pra54493	Phytophthora ramorum	364	70	0.242068
PITG_19619	Pra72134	Phytophthora ramorum	362	68	0.287632
PITG_19619	Pso135143	Phytophthora sojae	323	29	0.252458
PITG_19620	Pra72132	Phytophthora ramorum	382	5	0.145306
PITG_19620	Pra41820	Phytophthora ramorum	343	34	0.233452
PITG_19620	Pra54493	Phytophthora ramorum	364	13	0.234013
PITG_19620	Pra83565	Phytophthora ramorum	363	14	0.24969
PITG_19620	Pra83564	Phytophthora ramorum	363	14	0.24969
PITG_19620	Pso145625	Phytophthora sojae	366	11	0.181697
PITG_19620	Pso108850	Phytophthora sojae	375	2	0.188119
PITG_19620	Pso109634	Phytophthora sojae	305	72	0.232175
PITG_19620	Pso116769	Phytophthora sojae	369	8	0.239011
PITG_19620	Pso108849	Phytophthora sojae	406	29	0.240725
PITG_19623	Pra72132	Phytophthora ramorum	382	3	0.275794
PITG_19623	Pra72133	Phytophthora ramorum	383	4	0.29891
PITG_19623	Pso135151	Phytophthora sojae	410	31	0.205268
PITG_19623	Pso108849	Phytophthora sojae	406	27	0.26233
PITG_19623	Pso135149	Phytophthora sojae	406	27	0.26233
PITG_19623	Pso135147	Phytophthora sojae	406	27	0.26233
PITG_19623	Pso145625	Phytophthora sojae	366	13	0.291788
PITG_19624	Pra83568	Phytophthora ramorum	373	0	0.1587
PITG_19624	Pso135150	Phytophthora sojae	574	201	0.207154
PITG_19624	Pso108985	Phytophthora sojae	390	17	0.23438
PITG_19625	Pra54527	Phytophthora ramorum	330	43	0.252761
PITG_19625	Pra72132	Phytophthora ramorum	382	9	0.257311
PITG_19625	Pra72133	Phytophthora ramorum	383	10	0.265146
PITG_19625	Pso135147	Phytophthora sojae	406	33	0.180663
PITG_19625	Pso108849	Phytophthora sojae	406	33	0.180663
PITG_19625	Pso135149	Phytophthora sojae	406	33	0.180663
PITG_19625	Pso135151	Phytophthora sojae	410	37	0.217673
PITG_19625	Pso145625	Phytophthora sojae	366	7	0.270928
PITG_19634.1	Pra72132	Phytophthora ramorum	382	165	0.189175
PITG_19634.1	Pra72133	Phytophthora ramorum	383	166	0.220149
PITG_19634.1	Pra41820	Phytophthora ramorum	343	126	0.281402
PITG_19634.1	Pra83568	Phytophthora ramorum	373	156	0.281704

PITG_19634.1	Pra54493	Phytophthora ramorum	364	147	0.283088
PITG_19634.1	Pso135147	Phytophthora sojae	406	189	0.149728
PITG_19634.1	Pso108849	Phytophthora sojae	406	189	0.149728
PITG_19634.1	Pso135149	Phytophthora sojae	406	189	0.149728
PITG_19634.1	Pso145625	Phytophthora sojae	366	149	0.166139
PITG_19634.1	Pso108850	Phytophthora sojae	375	158	0.18017
PITG_19636	Pra72133	Phytophthora ramorum	383	225	0.187753
PITG_19636	Pra72132	Phytophthora ramorum	382	224	0.253303
PITG_19636	Pra83568	Phytophthora ramorum	373	215	0.296842
PITG_19636	Pso135151	Phytophthora sojae	410	252	0.184077
PITG_19636	Pso145625	Phytophthora sojae	366	208	0.236731
PITG_19636	Pso108850	Phytophthora sojae	375	217	0.245511
PITG_19636	Pso135147	Phytophthora sojae	406	248	0.263866
PITG_19636	Pso108849	Phytophthora sojae	406	248	0.263866
PITG_19637	Pra72132	Phytophthora ramorum	382	128	0.200276
PITG_19637	Pso145625	Phytophthora sojae	366	112	0.235988
PITG_19637	Pso108850	Phytophthora sojae	375	121	0.241737
PITG_19637	Pso108849	Phytophthora sojae	406	152	0.269293
PITG_19637	Pso135149	Phytophthora sojae	406	152	0.269293
PITG_19637	Pso135147	Phytophthora sojae	406	152	0.269293
PITG_19649	Pra72130	Phytophthora ramorum	368	86	0.171348
PITG_19649	Pra83562	Phytophthora ramorum	391	109	0.277482
PITG_19649	Pso116776	Phytophthora sojae	366	84	0.127407
PITG_19653	Pra41921	Phytophthora ramorum	125	124	0.15422
PITG_19653	Pra54493	Phytophthora ramorum	364	115	0.203734
PITG_19653	Pra83565	Phytophthora ramorum	363	114	0.215165
PITG_19653	Pra83564	Phytophthora ramorum	363	114	0.219979
PITG_19653	Pra41820	Phytophthora ramorum	343	94	0.222408
PITG_19653	Pso116769	Phytophthora sojae	369	120	0.191205
PITG_19653	Pso135143	Phytophthora sojae	323	74	0.250895
PITG_19653	Pso109634	Phytophthora sojae	305	56	0.293354
PITG_19782.1	Pra83050	Phytophthora ramorum	402	31	0.191575
PITG_19782.1	Pso127158	Phytophthora sojae	630	259	0.258136
PITG_19939	Pra52439	Phytophthora ramorum	528	53	0.180769
PITG_19939	Pra81639	Phytophthora ramorum	542	39	0.238131
PITG_19939	Pra81640	Phytophthora ramorum	542	39	0.238953

PITG_19939	Pso108522	Phytophthora sojae	541	40	0.156323
PITG_19939	Pso130485	Phytophthora sojae	455	126	0.269402
PITG_19939	Pso130491	Phytophthora sojae	532	49	0.278642
PITG_19985	Pra72130	Phytophthora ramorum	368	85	0.201923
PITG_19985	Pso116776	Phytophthora sojae	366	83	0.157781
PITG_20005.1	Pra86539	Phytophthora ramorum	268	405	0.188224
PITG_20005.1	Pra84162	Phytophthora ramorum	210	463	0.206252
PITG_20005.1	Pra39549	Phytophthora ramorum	52	621	0.290782
PITG_20319	Pso141361	Phytophthora sojae	328	0	0.205987
PITG_20319	Pso145358	Phytophthora sojae	328	0	0.209706
PITG_20320	Pso141360	Phytophthora sojae	334	103	0.293961
PITG_20320	Pso145359	Phytophthora sojae	334	103	0.293961
PITG_20325	Pra71253	Phytophthora ramorum	310	3	0.276915
PITG_20520	Pra82625	Phytophthora ramorum	643	126	0.206383
PITG_20520	Pso142360	Phytophthora sojae	514	3	0.183631
PITG_20676	Pra73436	Phytophthora ramorum	808	2	0.141791
PITG_20676	Pra86694	Phytophthora ramorum	300	510	0.209048
PITG_20676	Pso144049	Phytophthora sojae	806	4	0.133676
PITG_21028	Pra84565	Phytophthora ramorum	898	604	0.21381
PITG_21028	Pso136691	Phytophthora sojae	906	612	0.230136
PITG_21245	Pra72132	Phytophthora ramorum	382	1	0.121025
PITG_21245	Pra41820	Phytophthora ramorum	343	38	0.237192
PITG_21245	Pra54493	Phytophthora ramorum	364	17	0.237609
PITG_21245	Pra83565	Phytophthora ramorum	363	18	0.253373
PITG_21245	Pra83564	Phytophthora ramorum	363	18	0.253373
PITG_21245	Pso145625	Phytophthora sojae	366	15	0.181605
PITG_21245	Pso108850	Phytophthora sojae	375	6	0.188026
PITG_21245	Pso116769	Phytophthora sojae	369	12	0.235848
PITG_21245	Pso109634	Phytophthora sojae	305	76	0.236672
PITG_21245	Pso135149	Phytophthora sojae	406	25	0.238255
PITG_21247	Pra54527	Phytophthora ramorum	330	32	0.221091
PITG_21247	Pso135145	Phytophthora sojae	283	79	0.187505
PITG_21247	Pso108848	Phytophthora sojae	362	0	0.29008
PITG_21433	Pra79077	Phytophthora ramorum	789	498	0.100538
PITG_21433	Pra79076	Phytophthora ramorum	789	498	0.100538
PITG_21433	Pra78296	Phytophthora ramorum	788	497	0.16646

PITG_21433	Pra86922	Phytophthora ramorum	788	497	0.16646
PITG_21433	Pra78297	Phytophthora ramorum	788	497	0.16646
PITG_21554	Pra86970	Phytophthora ramorum	328	142	0.258491
PITG_21660	Pra82934	Phytophthora ramorum	750	49	0.205182
PITG_21660	Pso108895	Phytophthora sojae	577	124	0.099548
PITG_21743.1	Pra46413	Phytophthora ramorum	191	166	0.236612
PITG_21743.1	Pra86733	Phytophthora ramorum	356	1	0.252508
PITG_21743.1	Pra72388	Phytophthora ramorum	356	1	0.257001
PITG_21743.1	Pso109686	Phytophthora sojae	356	1	0.169841
PITG_21743.1	Pso109706	Phytophthora sojae	356	1	0.218077
PITG_21743.1	Pso108331	Phytophthora sojae	356	1	0.246524
PITG_21743.1	Pso109585	Phytophthora sojae	355	2	0.248337
PITG_21743.1	Pso109744	Phytophthora sojae	356	1	0.252045
PITG_21744.1	Pra46413	Phytophthora ramorum	191	165	0.064686
PITG_21744.1	Pra86733	Phytophthora ramorum	356	0	0.22995
PITG_21744.1	Pra72388	Phytophthora ramorum	356	0	0.248265
PITG_21744.1	Pso109706	Phytophthora sojae	356	0	0.066221
PITG_21744.1	Pso125904	Phytophthora sojae	131	225	0.072756
PITG_21744.1	Pso109686	Phytophthora sojae	356	0	0.125315
PITG_21744.1	Pso108331	Phytophthora sojae	356	0	0.217521
PITG_21744.1	Pso109744	Phytophthora sojae	356	0	0.242421
PITG_21925	Pra41921	Phytophthora ramorum	125	114	0.163091
PITG_21925	Pra83564	Phytophthora ramorum	363	124	0.183919
PITG_21925	Pra83565	Phytophthora ramorum	363	124	0.187848
PITG_21925	Pra41820	Phytophthora ramorum	343	104	0.191786
PITG_21925	Pra54493	Phytophthora ramorum	364	125	0.238319
PITG_21925	Pso135143	Phytophthora sojae	323	84	0.247965
PITG_21925	Pso116769	Phytophthora sojae	369	130	0.296272
PITG_21986	Pra72030	Phytophthora ramorum	230	127	0.130219
PITG_21986	Pra52767	Phytophthora ramorum	226	123	0.139635
PITG_21986	Pso109681	Phytophthora sojae	242	139	0.162269
PITG_22085.1	Pra73896	Phytophthora ramorum	815	372	0.197507
PITG_22085.1	Pso127729	Phytophthora sojae	851	408	0.21604
PITG_22095	Pra83174	Phytophthora ramorum	778	219	0.136547
PITG_22095	Pra84532	Phytophthora ramorum	507	52	0.139678
PITG_22095	Pra78230	Phytophthora ramorum	785	226	0.275259

PITG_22095	Pra78231	Phytophthora ramorum	814	255	0.294432
PITG_22095	Pso145641	Phytophthora sojae	327	232	0.165122
PITG_22095	Pso142760	Phytophthora sojae	781	222	0.208161
PITG_22095	Pso143984	Phytophthora sojae	709	150	0.249866
PITG_22095	Pso133712	Phytophthora sojae	700	141	0.267858
PITG_22095	Pso133711	Phytophthora sojae	816	257	0.283988
PITG_22202	Pra72030	Phytophthora ramorum	230	127	0.130219
PITG_22202	Pra52767	Phytophthora ramorum	226	123	0.139635
PITG_22202	Pso109681	Phytophthora sojae	242	139	0.162269
