# Supplementary material for: The CAZyome of Phytophthora spp.: A comprehensive analysis of the gene complement coding for carbohydrate-active enzymes in species of the genus Phytophthora
Source: BMC Genomics. 2010 Sep 28;11:525. doi: 10.1186/1471-2164-11-525 (PMC2997016; doi:10.1186/1471-2164-11-525)
Supplement: Additional file 6 — Phytophthora GT orthologs. Phytophthora GT orthologs as determined using the Phylogenetic Resources for the Interpretation of Genomes (PHRINGE). In most cases, more than one orthologous gene was found in each of the three Phytophthora species. A total of 72 members had orthologs in both, P. sojae and P. ramorum genomes while three had orthologs in P. sojae only. [file 1471-2164-11-525-S6.PDF]

GLYCOSYL TRANSFERASES

Gene Identifier	Matching Gene	Source Organism	Length	Difference in Length	Distance Score
PITG_00632.1	Pra72878	Phytophthora ramorum	885	34	0.043103
PITG_00632.1	Pso140975	Phytophthora sojae	889	38	0.067918
PITG_00983.1	Pra94933	Phytophthora ramorum	1204	150	0.260135
PITG_00983.1	Pso155230	Phytophthora sojae	1246	108	0.264699
PITG_01031.1	Pra82490	Phytophthora ramorum	678	4	0.019948
PITG_01031.1	Pso127165	Phytophthora sojae	686	4	0.027085
PITG_01416.1	Pra71427	Phytophthora ramorum	510	0	0.034436
PITG_01416.1	Pso109079	Phytophthora sojae	510	0	0.02666
PITG_01869.1	Pra73809	Phytophthora ramorum	800	34	0.474243
PITG_01869.1	Pso137445	Phytophthora sojae	872	38	0.322403
PITG_02050.1	Pra77229	Phytophthora ramorum	1026	112	0.099825
PITG_02050.1	Pso143614	Phytophthora sojae	914	0	0.082237
PITG_02294.1	Pra95737	Phytophthora ramorum	599	62	0.164688
PITG_02294.1	Pso128422	Phytophthora sojae	574	37	0.159075
PITG_02307.1	Pra80488	Phytophthora ramorum	1171	42	0.092558
PITG_02307.1	Pso128418	Phytophthora sojae	1266	137	0.082283
PITG_02432.1	Pra45645	Phytophthora ramorum	372	198	0.093507
PITG_02432.1	Pso110975	Phytophthora sojae	337	233	0.065797
PITG_02575.1	Pra77528	Phytophthora ramorum	999	36	0.177892
PITG_02575.1	Pso142040	Phytophthora sojae	999	36	0.169617
PITG_02952.1	Pra50162	Phytophthora ramorum	393	35	0.017523
PITG_02952.1	Pso136217	Phytophthora sojae	1417	989	0.025228
PITG_03060.1	Pso139395	Phytophthora sojae	398	32	0.256665
PITG_03335.1	Pra76180	Phytophthora ramorum	2641	9	0.122059
PITG_03335.1	Pso134235	Phytophthora sojae	2657	25	0.110106
PITG_03335.1	Pso134234	Phytophthora sojae	1913	719	0.126803
PITG_03348.1	Pra76168	Phytophthora ramorum	1165	2	0.063071
PITG_03348.1	Pra76170	Phytophthora ramorum	1062	105	0.10778
PITG_03348.1	Pso145066	Phytophthora sojae	943	224	0.036018
PITG_03473.1	Pra76056	Phytophthora ramorum	2060	33	0.07368
PITG_03473.1	Pra76057	Phytophthora ramorum	2042	15	0.374117
PITG_03473.1	Pso134121	Phytophthora sojae	2026	1	0.065687
PITG_03473.1	Pso134122	Phytophthora sojae	2150	123	0.358178
PITG_03587.1	Pra81409	Phytophthora ramorum	1374	269	0.179716

PITG_03587.1	Pso134825	Phytophthora sojae	201	904	0.17449
PITG_04610.1	Pra38947	Phytophthora ramorum	717	15	0.158164
PITG_04610.1	Pso116260	Phytophthora sojae	730	2	0.147434
PITG_04917.1	Pra40970	Phytophthora ramorum	476	156	0.090867
PITG_04917.1	Pso158535	Phytophthora sojae	1118	486	0.154166
PITG_05079.1	Pra82372	Phytophthora ramorum	2248	0	0.076573
PITG_05079.1	Pra77994	Phytophthora ramorum	2224	24	0.464962
PITG_05079.1	Pso139198	Phytophthora sojae	2247	1	0.07016
PITG_05079.1	Pso141900	Phytophthora sojae	2229	19	0.471209
PITG_05541.1	Pra43914	Phytophthora ramorum	193	336	0.120038
PITG_05541.1	Pso108422	Phytophthora sojae	476	53	0.126094
PITG_05763.1	Pra75925	Phytophthora ramorum	1593	28	0.114825
PITG_05763.1	Pso132515	Phytophthora sojae	1604	17	0.128308
PITG_06358.1	Pra80843	Phytophthora ramorum	2401	44	0.133343
PITG_06358.1	Pso143054	Phytophthora sojae	2456	11	0.150783
PITG_06428.1	Pra77350	Phytophthora ramorum	2283	4	0.087929
PITG_06428.1	Pso134329	Phytophthora sojae	2279	8	0.08552
PITG_06491.1	Pra77385	Phytophthora ramorum	1189	9	0.240495
PITG_06491.1	Pso134371	Phytophthora sojae	875	305	0.223165
PITG_06828.1	Pra82938	Phytophthora ramorum	1150	3	0.094011
PITG_06828.1	Pso135706	Phytophthora sojae	1186	33	0.091298
PITG_06984.1	Pra79961	Phytophthora ramorum	302	4	0.141347
PITG_06984.1	Pso144888	Phytophthora sojae	301	3	0.123557
PITG_06984.1	Pso138740	Phytophthora sojae	301	3	0.123557
PITG_07104.1	Pra83795	Phytophthora ramorum	535	141	0.114846
PITG_07104.1	Pso143495	Phytophthora sojae	554	160	0.105863
PITG_07201.1	Pra94673	Phytophthora ramorum	1496	137	0.20855
PITG_07201.1	Pso109593	Phytophthora sojae	69	1564	0.426269
PITG_07702.1	Pra96131	Phytophthora ramorum	548	32	0.121067
PITG_07702.1	Pso157350	Phytophthora sojae	778	198	0.120818
PITG_09296.1	Pra80101	Phytophthora ramorum	2424	228	0.194465
PITG_09296.1	Pra40606	Phytophthora ramorum	441	1755	0.292097
PITG_09296.1	Pra96916	Phytophthora ramorum	551	1645	0.383303
PITG_09296.1	Pso141158	Phytophthora sojae	2215	19	0.183224
PITG_09296.1	Pso141209	Phytophthora sojae	535	1661	0.49346
PITG_09367.1	Pra84481	Phytophthora ramorum	1364	38	0.276617

PITG_09367.1	Pra40606	Phytophthora ramorum	441	961	0.276841
PITG_09367.1	Pra96916	Phytophthora ramorum	551	851	0.32523
PITG_09367.1	Pra87277	Phytophthora ramorum	1095	307	0.331875
PITG_09367.1	Pso145323	Phytophthora sojae	336	1066	0.188465
PITG_09367.1	Pso141209	Phytophthora sojae	535	867	0.19595
PITG_09367.1	Pso141210	Phytophthora sojae	991	411	0.279517
PITG_09367.1	Pso141208	Phytophthora sojae	495	907	0.34122
PITG_09389.1	Pra84498	Phytophthora ramorum	1209	16	0.100609
PITG_09389.1	Pso141232	Phytophthora sojae	1280	55	0.076493
PITG_09412.1	Pra40606	Phytophthora ramorum	441	878	0.129628
PITG_09412.1	Pra96916	Phytophthora ramorum	551	768	0.170441
PITG_09412.1	Pso142345	Phytophthora sojae	4995	3676	0.198084
PITG_09412.1	Pso145323	Phytophthora sojae	336	983	0.252494
PITG_09412.1	Pso141209	Phytophthora sojae	535	784	0.293263
PITG_09412.1	Pso137758	Phytophthora sojae	1515	196	0.328399
PITG_09412.1	Pso137756	Phytophthora sojae	339	980	0.368647
PITG_09413.1	Pra84690	Phytophthora ramorum	1358	152	0.186643
PITG_09413.1	Pra40606	Phytophthora ramorum	441	1069	0.291271
PITG_09413.1	Pra96916	Phytophthora ramorum	551	959	0.409958
PITG_09413.1	Pso142346	Phytophthora sojae	1507	3	0.167687
PITG_09963.1	Pra76908	Phytophthora ramorum	995	87	0.060531
PITG_09963.1	Pra76907	Phytophthora ramorum	1029	53	0.3814
PITG_09963.1	Pso131832	Phytophthora sojae	163	919	0.082725
PITG_09964.1	Pra96524	Phytophthora ramorum	2468	1325	0.050941
PITG_09964.1	Pso158132	Phytophthora sojae	2608	1465	0.057362
PITG_10344.1	Pra96233	Phytophthora ramorum	1994	1249	0.329034
PITG_10344.1	Pso138310	Phytophthora sojae	827	82	0.235342
PITG_11398.1	Pso145635	Phytophthora sojae	293	155	0.465182
PITG_11648.1	Pra46727	Phytophthora ramorum	418	236	0.084247
PITG_11648.1	Pso113542	Phytophthora sojae	407	247	0.094604
PITG_11648.1	Pso131391	Phytophthora sojae	195	459	0.182209
PITG_11723.1	Pra93592	Phytophthora ramorum	1390	4126	0.118481
PITG_11723.1	Pra93591	Phytophthora ramorum	736	4780	0.201565
PITG_11723.1	Pso156256	Phytophthora sojae	2349	3167	0.108007
PITG_11723.1	Pso156257	Phytophthora sojae	750	4766	0.202398
PITG_12110.1	Pra74660	Phytophthora ramorum	1280	612	0.1382

PITG_12110.1	Pso129478	Phytophthora sojae	4201	3533	0.095618
PITG_13172.2	Pra94555	Phytophthora ramorum	315	119	0.277674
PITG_13172.3	Pso155373	Phytophthora sojae	398	36	0.269332
PITG_13291.1	Pra87257	Phytophthora ramorum	274	23	0.090507
PITG_13291.1	Pso137050	Phytophthora sojae	575	278	0.094134
PITG_13721.1	Pra81662	Phytophthora ramorum	1294	86	0.107855
PITG_13721.1	Pso130468	Phytophthora sojae	1825	617	0.098956
PITG_13917.1	Pso136425	Phytophthora sojae	452	71	0.435052
PITG_14473.1	Pra78730	Phytophthora ramorum	606	26	0.188688
PITG_14473.1	Pso133979	Phytophthora sojae	612	32	0.192678
PITG_14473.1	Pso133978	Phytophthora sojae	563	17	0.403678
PITG_14483.1	Pra78738	Phytophthora ramorum	514	17	0.448861
PITG_14483.1	Pso133987	Phytophthora sojae	458	73	0.459584
PITG_14483.1	Pso133975	Phytophthora sojae	301	230	0.480037
PITG_14727.1	Pra77994	Phytophthora ramorum	2224	5	0.072232
PITG_14727.1	Pra82372	Phytophthora ramorum	2248	19	0.463365
PITG_14727.1	Pso141900	Phytophthora sojae	2229	0	0.068635
PITG_14727.1	Pso139198	Phytophthora sojae	2247	18	0.466264
PITG_15882.1	Pra71457	Phytophthora ramorum	505	500	0.12417
PITG_15882.1	Pso120536	Phytophthora sojae	470	535	0.02828
PITG_16984.1	Pra83142	Phytophthora ramorum	1020	0	0.042593
PITG_16984.1	Pso140288	Phytophthora sojae	1023	3	0.047144
PITG_17007.1	Pra96524	Phytophthora ramorum	2468	1325	0.050941
PITG_17007.1	Pso158132	Phytophthora sojae	2608	1465	0.057362
PITG_17013.1	Pra83116	Phytophthora ramorum	564	315	0.079164
PITG_17013.1	Pra83117	Phytophthora ramorum	289	590	0.367993
PITG_17013.1	Pso140327	Phytophthora sojae	560	319	0.08225
PITG_17661.1	Pra95021	Phytophthora ramorum	536	83	0.195785
PITG_17661.1	Pso130863	Phytophthora sojae	683	64	0.229046
PITG_17663.1	Pra78113	Phytophthora ramorum	624	2	0.108256
PITG_17663.1	Pso130858	Phytophthora sojae	625	3	0.105143
PITG_17664.1	Pra78112	Phytophthora ramorum	598	64	0.219384
PITG_17664.1	Pso130862	Phytophthora sojae	626	92	0.203204
PITG_17928.1	Pra75562	Phytophthora ramorum	1013	35	0.053756
PITG_17928.1	Pso143341	Phytophthora sojae	1015	37	0.063223
PITG_18015.1	Pra74432	Phytophthora ramorum	521	1	0.114547

PITG_18015.1	Pso140071	Phytophthora sojae	526	6	0.124897
PITG_18105.1	Pra94968	Phytophthora ramorum	1947	1418	0.164853
PITG_18105.1	Pso137236	Phytophthora sojae	493	36	0.182772
PITG_18106.1	Pra94968	Phytophthora ramorum	1947	2587	0.090499
PITG_18106.1	Pra77868	Phytophthora ramorum	463	4071	0.194004
PITG_18106.1	Pso157557	Phytophthora sojae	1721	2813	0.10896
PITG_18106.1	Pso157556	Phytophthora sojae	444	4090	0.164873
PITG_18255.1	Pso130784	Phytophthora sojae	475	72	0.420637
PITG_18258.1	Pra73627	Phytophthora ramorum	612	10	0.32413
PITG_18258.1	Pso130785	Phytophthora sojae	627	5	0.354468
PITG_18266.1	Pra73621	Phytophthora ramorum	337	43	0.357632
PITG_18266.1	Pso130803	Phytophthora sojae	582	202	0.354623
PITG_18270.1	Pra93659	Phytophthora ramorum	588	190	0.268714
PITG_18270.1	Pso130801	Phytophthora sojae	597	199	0.243728
PITG_18271.1	Pra73619	Phytophthora ramorum	1770	1205	0.283997
PITG_18271.1	Pso130800	Phytophthora sojae	576	11	0.325083
PITG_18272.1	Pra73619	Phytophthora ramorum	1770	1188	0.286829
PITG_18272.1	Pso130799	Phytophthora sojae	1100	518	0.290481
PITG_18275.1	Pra73616	Phytophthora ramorum	1708	1049	0.301711
PITG_18275.1	Pso130790	Phytophthora sojae	1656	997	0.300267
PITG_18276.1	Pra73615	Phytophthora ramorum	747	204	0.178308
PITG_18276.1	Pso130791	Phytophthora sojae	740	197	0.212093
PITG_18277.1	Pra73614	Phytophthora ramorum	653	44	0.268479
PITG_18277.1	Pso130792	Phytophthora sojae	647	38	0.266273
PITG_18279.1	Pra73611	Phytophthora ramorum	882	276	0.281565
PITG_18279.1	Pra73612	Phytophthora ramorum	367	791	0.311092
PITG_18279.1	Pso130793	Phytophthora sojae	371	787	0.284849
PITG_18279.1	Pso130794	Phytophthora sojae	867	291	0.297923
PITG_20927.1	Pra85749	Phytophthora ramorum	1426	600	0.084625
PITG_20927.1	Pso144346	Phytophthora sojae	1646	820	0.061996
PITG_21507.1	Pra96916	Phytophthora ramorum	551	490	0.162951
PITG_21507.1	Pra40606	Phytophthora ramorum	441	600	0.358469
PITG_21507.1	Pso137758	Phytophthora sojae	1515	474	0.299474
PITG_21507.1	Pso145323	Phytophthora sojae	336	705	0.431254
PITG_21507.1	Pso141209	Phytophthora sojae	535	506	0.442921
PITG_21507.1	Pso142345	Phytophthora sojae	4995	3954	0.463349

PITG_21941.1	Pra77928	Phytophthora ramorum	388	150	0.356991
PITG_21941.1	Pso139395	Phytophthora sojae	398	160	0.25966
PITG_21962.1	Pra73809	Phytophthora ramorum	800	102	0.113243
PITG_21962.1	Pso137445	Phytophthora sojae	872	30	0.083168
PITG_22471.1	Pra84481	Phytophthora ramorum	1364	1169	0.154849
PITG_22471.1	Pra96916	Phytophthora ramorum	551	356	0.305719
PITG_22471.1	Pra80101	Phytophthora ramorum	2424	2229	0.346419
PITG_22471.1	Pra84690	Phytophthora ramorum	1358	1163	0.377005
PITG_22471.1	Pra40606	Phytophthora ramorum	441	246	0.383551
PITG_22471.1	Pso141209	Phytophthora sojae	535	340	0.229982
PITG_22471.1	Pso141210	Phytophthora sojae	991	796	0.229982
PITG_22471.1	Pso141158	Phytophthora sojae	2215	2020	0.298714
PITG_22471.1	Pso137758	Phytophthora sojae	1515	1320	0.305214
PITG_22471.1	Pso145323	Phytophthora sojae	336	141	0.309629
PITG_22649.1	Pra46727	Phytophthora ramorum	418	248	0.259922
PITG_22649.1	Pso113542	Phytophthora sojae	407	237	0.291717
