# Supplementary material for: The CAZyome of Phytophthora spp.: A comprehensive analysis of the gene complement coding for carbohydrate-active enzymes in species of the genus Phytophthora
Source: BMC Genomics. 2010 Sep 28;11:525. doi: 10.1186/1471-2164-11-525 (PMC2997016; doi:10.1186/1471-2164-11-525)
Supplement: Additional file 7 — Phytophthora PL orthologs. Phytophthora PL orthologs. Phytophthora PL orthologs as determined using the Phylogenetic Resources for the Interpretation of Genomes (PHRINGE). In most cases, more than one orthologous gene was found in each of the three Phytophthora species. A total of 56 (out of 59) P. infestans PL-coding genes had orthologs either in both, P. sojae and P. ramorum (53), or in P. ramorum only (3). [file 1471-2164-11-525-S7.PDF]

POLYSACCHARIDE LYASES

Gene Identifier	Matching Gene	Source Organism	Length	Difference in Length	Distance Score
PITG_00872.1	Pra45942	Phytophthora ramorum	343	173	0.329325
PITG_02332.1	Pra84662	Phytophthora ramorum	583	35	0.251769
PITG_02332.1	Pra84661	Phytophthora ramorum	541	7	0.481419
PITG_02332.1	Pso128396	Phytophthora sojae	592	44	0.239043
PITG_02368.1	Pra72397	Phytophthora ramorum	542	55	0.226467
PITG_02368.1	Pra86292	Phytophthora ramorum	545	58	0.335096
PITG_02368.1	Pra84663	Phytophthora ramorum	545	58	0.336011
PITG_02368.1	Pso128367	Phytophthora sojae	541	54	0.192023
PITG_04668.1	Pra84933	Phytophthora ramorum	300	6	0.211527
PITG_07680.1	Pra75227	Phytophthora ramorum	441	137	0.403984
PITG_07680.1	Pra75226	Phytophthora ramorum	451	147	0.458804
PITG_07680.1	Pso145395	Phytophthora sojae	442	138	0.462361
PITG_07680.1	Pso144173	Phytophthora sojae	427	123	0.467838
PITG_07680.1	Pso144196	Phytophthora sojae	457	153	0.473064
PITG_07680.1	Pso144197	Phytophthora sojae	457	153	0.473064
PITG_08555.1	Pra85622	Phytophthora ramorum	420	145	0.300793
PITG_08555.1	Pra85623	Phytophthora ramorum	939	664	0.37215
PITG_08555.1	Pra86921	Phytophthora ramorum	513	238	0.447537
PITG_08555.1	Pso140272	Phytophthora sojae	974	699	0.319686
PITG_08555.1	Pso132225	Phytophthora sojae	974	699	0.319686
PITG_08555.1	Pso145391	Phytophthora sojae	824	549	0.336769
PITG_08555.1	Pso140268	Phytophthora sojae	444	169	0.337903
PITG_08555.1	Pso141647	Phytophthora sojae	402	127	0.3397
PITG_08561.1	Pra86921	Phytophthora ramorum	513	297	0.223551
PITG_08561.1	Pra85622	Phytophthora ramorum	420	204	0.237572
PITG_08561.1	Pra77010	Phytophthora ramorum	874	658	0.309834
PITG_08561.1	Pra85623	Phytophthora ramorum	939	723	0.341126
PITG_08561.1	Pso132227	Phytophthora sojae	485	269	0.077832
PITG_08561.1	Pso145391	Phytophthora sojae	824	608	0.139363
PITG_08561.1	Pso140272	Phytophthora sojae	974	758	0.214237
PITG_08561.1	Pso132225	Phytophthora sojae	974	758	0.214237
PITG_08561.1	Pso140268	Phytophthora sojae	444	228	0.221687
PITG_08563.1	Pra85626	Phytophthora ramorum	380	63	0.307744
PITG_08563.1	Pso132222	Phytophthora sojae	385	68	0.165292
PITG_08563.1	Pso132218	Phytophthora sojae	421	104	0.229336
PITG_08563.1	Pso133199	Phytophthora sojae	425	108	0.24012
PITG_08563.1	Pso145592	Phytophthora sojae	203	114	0.280435

PITG_08564.1	Pra85626	Phytophthora ramorum	380	3	0.233428
PITG_08564.1	Pso132222	Phytophthora sojae	385	2	0.219886
PITG_08564.1	Pso132218	Phytophthora sojae	421	38	0.227943
PITG_08564.1	Pso133199	Phytophthora sojae	425	42	0.253179
PITG_08632.1	Pra86169	Phytophthora ramorum	629	424	0.123838
PITG_08632.1	Pra77024	Phytophthora ramorum	653	448	0.123975
PITG_08632.1	Pra44512	Phytophthora ramorum	223	18	0.130105
PITG_08632.1	Pra43398	Phytophthora ramorum	218	13	0.13398
PITG_08632.1	Pra87411	Phytophthora ramorum	557	352	0.139593
PITG_08632.1	Pso144923	Phytophthora sojae	656	451	0.121146
PITG_08632.1	Pso132161	Phytophthora sojae	255	50	0.129092
PITG_08632.1	Pso132158	Phytophthora sojae	255	50	0.149847
PITG_08632.1	Pso132157	Phytophthora sojae	701	496	0.182476
PITG_08632.1	Pso132156	Phytophthora sojae	701	496	0.182476
PITG_08633.1	Pra44446	Phytophthora ramorum	181	24	0.424012
PITG_08633.1	Pra44487	Phytophthora ramorum	219	62	0.449207
PITG_08633.1	Pra44483	Phytophthora ramorum	226	69	0.449207
PITG_08633.1	Pra87411	Phytophthora ramorum	557	400	0.493836
PITG_08633.1	Pra77024	Phytophthora ramorum	653	496	0.498539
PITG_08633.1	Pso132156	Phytophthora sojae	701	544	0.415948
PITG_08633.1	Pso132157	Phytophthora sojae	701	544	0.415948
PITG_08633.1	Pso140239	Phytophthora sojae	396	239	0.46864
PITG_08633.1	Pso140241	Phytophthora sojae	369	212	0.46864
PITG_08633.1	Pso114189	Phytophthora sojae	193	36	0.478374
PITG_08634.1	Pra44446	Phytophthora ramorum	181	27	0.18586
PITG_08634.1	Pra44487	Phytophthora ramorum	219	65	0.189152
PITG_08634.1	Pra44483	Phytophthora ramorum	226	72	0.189152
PITG_08634.1	Pra42333	Phytophthora ramorum	168	14	0.193966
PITG_08634.1	Pra77022	Phytophthora ramorum	679	525	0.213085
PITG_08634.1	Pso132156	Phytophthora sojae	701	547	0.162353
PITG_08634.1	Pso132157	Phytophthora sojae	701	547	0.162353
PITG_08634.1	Pso114189	Phytophthora sojae	193	39	0.185393
PITG_08634.1	Pso140239	Phytophthora sojae	396	242	0.21729
PITG_08634.1	Pso140241	Phytophthora sojae	369	215	0.21729
PITG_08638.1	Pra44485	Phytophthora ramorum	180	6	0.080932
PITG_08638.1	Pra77022	Phytophthora ramorum	679	505	0.153695
PITG_08638.1	Pra43398	Phytophthora ramorum	218	44	0.153695
PITG_08638.1	Pra44512	Phytophthora ramorum	223	49	0.208454
PITG_08638.1	Pra87411	Phytophthora ramorum	557	383	0.208454

PITG_08638.1	Pso132157	Phytophthora sojae	701	527	0.120853
PITG_08638.1	Pso132156	Phytophthora sojae	701	527	0.120853
PITG_08638.1	Pso144923	Phytophthora sojae	656	482	0.194021
PITG_08638.1	Pso132158	Phytophthora sojae	255	81	0.218232
PITG_08638.1	Pso132161	Phytophthora sojae	255	81	0.219437
PITG_08646.1	Pra42333	Phytophthora ramorum	168	7	0.127023
PITG_08646.1	Pra44446	Phytophthora ramorum	181	6	0.132953
PITG_08646.1	Pra44487	Phytophthora ramorum	219	44	0.151486
PITG_08646.1	Pra77024	Phytophthora ramorum	653	478	0.152083
PITG_08646.1	Pra86169	Phytophthora ramorum	629	454	0.152198
PITG_08646.1	Pso132157	Phytophthora sojae	701	526	0.146325
PITG_08646.1	Pso132156	Phytophthora sojae	701	526	0.146325
PITG_08646.1	Pso114189	Phytophthora sojae	193	18	0.152969
PITG_08646.1	Pso140239	Phytophthora sojae	396	221	0.188097
PITG_08646.1	Pso140241	Phytophthora sojae	369	194	0.188097
PITG_08647.1	Pra44485	Phytophthora ramorum	180	1	0.061809
PITG_08647.1	Pra77022	Phytophthora ramorum	679	498	0.147884
PITG_08647.1	Pra43398	Phytophthora ramorum	218	37	0.150033
PITG_08647.1	Pra44512	Phytophthora ramorum	223	42	0.206968
PITG_08647.1	Pra87411	Phytophthora ramorum	557	376	0.217322
PITG_08647.1	Pso132156	Phytophthora sojae	701	520	0.116421
PITG_08647.1	Pso132157	Phytophthora sojae	701	520	0.116421
PITG_08647.1	Pso144923	Phytophthora sojae	656	475	0.173419
PITG_08647.1	Pso132161	Phytophthora sojae	255	74	0.210843
PITG_08647.1	Pso132158	Phytophthora sojae	255	74	0.228927
PITG_09254.1	Pra71773	Phytophthora ramorum	232	30	0.062101
PITG_09254.1	Pra80133	Phytophthora ramorum	262	0	0.077605
PITG_09254.1	Pra39825	Phytophthora ramorum	253	9	0.254297
PITG_09254.1	Pra74998	Phytophthora ramorum	263	1	0.268959
PITG_09254.1	Pra42333	Phytophthora ramorum	168	94	0.350029
PITG_09254.1	Pso140498	Phytophthora sojae	118	144	0.132516
PITG_09254.1	Pso109550	Phytophthora sojae	233	29	0.304333
PITG_09254.1	Pso143281	Phytophthora sojae	267	5	0.324291
PITG_09254.1	Pso122879	Phytophthora sojae	200	62	0.348734
PITG_09254.1	Pso114347	Phytophthora sojae	158	104	0.352219
PITG_09255.1	Pra80133	Phytophthora ramorum	262	127	0.036979
PITG_09255.1	Pra71773	Phytophthora ramorum	232	97	0.036979
PITG_09255.1	Pra39825	Phytophthora ramorum	253	118	0.184007
PITG_09255.1	Pra74998	Phytophthora ramorum	263	128	0.191239

PITG_09255.1	Pra86169	Phytophthora ramorum	629	494	0.301211
PITG_09255.1	Pso143281	Phytophthora sojae	267	132	0.220416
PITG_09255.1	Pso109550	Phytophthora sojae	233	98	0.230083
PITG_09255.1	Pso140241	Phytophthora sojae	369	234	0.262996
PITG_09255.1	Pso142501	Phytophthora sojae	573	438	0.282629
PITG_09255.1	Pso140239	Phytophthora sojae	396	261	0.285601
PITG_09256.1	Pra80133	Phytophthora ramorum	262	30	0.071278
PITG_09256.1	Pra71773	Phytophthora ramorum	232	0	0.075847
PITG_09256.1	Pra39825	Phytophthora ramorum	253	21	0.270167
PITG_09256.1	Pra74998	Phytophthora ramorum	263	31	0.274281
PITG_09256.1	Pra42333	Phytophthora ramorum	168	64	0.382092
PITG_09256.1	Pso140498	Phytophthora sojae	118	114	0.122385
PITG_09256.1	Pso143281	Phytophthora sojae	267	35	0.314279
PITG_09256.1	Pso109550	Phytophthora sojae	233	1	0.320448
PITG_09256.1	Pso122879	Phytophthora sojae	200	32	0.377757
PITG_09256.1	Pso114347	Phytophthora sojae	158	74	0.388345
PITG_09257.1	Pra71773	Phytophthora ramorum	232	15	0.056795
PITG_09257.1	Pra80133	Phytophthora ramorum	262	15	0.073551
PITG_09257.1	Pra39825	Phytophthora ramorum	253	6	0.260872
PITG_09257.1	Pra74998	Phytophthora ramorum	263	16	0.278872
PITG_09257.1	Pra44445	Phytophthora ramorum	226	21	0.406353
PITG_09257.1	Pso140498	Phytophthora sojae	118	129	0.143775
PITG_09257.1	Pso109550	Phytophthora sojae	233	14	0.311009
PITG_09257.1	Pso122879	Phytophthora sojae	200	47	0.337911
PITG_09257.1	Pso143281	Phytophthora sojae	267	20	0.339019
PITG_09257.1	Pso114189	Phytophthora sojae	193	54	0.341425
PITG_09258.1	Pra80133	Phytophthora ramorum	262	129	0.060911
PITG_09258.1	Pra71773	Phytophthora ramorum	232	99	0.068707
PITG_09258.1	Pra39825	Phytophthora ramorum	253	120	0.233356
PITG_09258.1	Pra74998	Phytophthora ramorum	263	130	0.240558
PITG_09258.1	Pra86169	Phytophthora ramorum	629	496	0.343202
PITG_09258.1	Pso140498	Phytophthora sojae	118	15	0.137554
PITG_09258.1	Pso143281	Phytophthora sojae	267	134	0.262127
PITG_09258.1	Pso109550	Phytophthora sojae	233	100	0.27234
PITG_09258.1	Pso114347	Phytophthora sojae	158	25	0.325624
PITG_09258.1	Pso142500	Phytophthora sojae	195	62	0.331464
PITG_09259.1	Pra80133	Phytophthora ramorum	262	44	0.07631
PITG_09259.1	Pra71773	Phytophthora ramorum	232	14	0.081211
PITG_09259.1	Pra39825	Phytophthora ramorum	253	35	0.286665

PITG_09259.1	Pra74998	Phytophthora ramorum	263	45	0.291042
PITG_09259.1	Pra42333	Phytophthora ramorum	168	50	0.402121
PITG_09259.1	Pso140498	Phytophthora sojae	118	100	0.152307
PITG_09259.1	Pso143281	Phytophthora sojae	267	49	0.328299
PITG_09259.1	Pso109550	Phytophthora sojae	233	15	0.334994
PITG_09259.1	Pso122879	Phytophthora sojae	200	18	0.389244
PITG_09259.1	Pso114347	Phytophthora sojae	158	60	0.409495
PITG_09457.1	Pra75227	Phytophthora ramorum	441	111	0.355923
PITG_09457.1	Pra75226	Phytophthora ramorum	451	121	0.372021
PITG_09457.1	Pra75225	Phytophthora ramorum	454	124	0.430648
PITG_09457.1	Pra39775	Phytophthora ramorum	344	14	0.443047
PITG_09457.1	Pra46219	Phytophthora ramorum	353	23	0.456414
PITG_09457.1	Pso144196	Phytophthora sojae	457	127	0.400614
PITG_09457.1	Pso144197	Phytophthora sojae	457	127	0.400614
PITG_09457.1	Pso144172	Phytophthora sojae	222	108	0.413951
PITG_09457.1	Pso145395	Phytophthora sojae	442	112	0.425286
PITG_09457.1	Pso144173	Phytophthora sojae	427	97	0.426213
PITG_09465.1	Pra75227	Phytophthora ramorum	441	39	0.26459
PITG_09465.1	Pra75226	Phytophthora ramorum	451	49	0.35397
PITG_09465.1	Pra75225	Phytophthora ramorum	454	52	0.398566
PITG_09465.1	Pra39775	Phytophthora ramorum	344	58	0.398954
PITG_09465.1	Pra46219	Phytophthora ramorum	353	49	0.411837
PITG_09465.1	Pso145395	Phytophthora sojae	442	40	0.242538
PITG_09465.1	Pso144173	Phytophthora sojae	427	25	0.247161
PITG_09465.1	Pso144196	Phytophthora sojae	457	55	0.365081
PITG_09465.1	Pso144197	Phytophthora sojae	457	55	0.369281
PITG_09465.1	Pso144172	Phytophthora sojae	222	180	0.395161
PITG_09466.1	Pra39775	Phytophthora ramorum	344	143	0.282225
PITG_09466.1	Pra46219	Phytophthora ramorum	353	152	0.302532
PITG_09466.1	Pra75231	Phytophthora ramorum	401	200	0.331967
PITG_09466.1	Pra75233	Phytophthora ramorum	142	59	0.353389
PITG_09466.1	Pra45942	Phytophthora ramorum	343	142	0.436345
PITG_09466.1	Pso124664	Phytophthora sojae	192	9	0.12035
PITG_09466.1	Pso144178	Phytophthora sojae	363	162	0.411906
PITG_09467.1	Pra46219	Phytophthora ramorum	353	162	0.332206
PITG_09467.1	Pra39775	Phytophthora ramorum	344	153	0.33245
PITG_09467.1	Pra75231	Phytophthora ramorum	401	210	0.333367
PITG_09467.1	Pso144178	Phytophthora sojae	363	172	0.350678
PITG_09467.1	Pso132222	Phytophthora sojae	385	194	0.480626

PITG_09467.1	Pso132218	Phytophthora sojae	421	230	0.491967
PITG_09821.1	Pra86169	Phytophthora ramorum	629	525	0.404464
PITG_09821.1	Pra87411	Phytophthora ramorum	557	453	0.404464
PITG_09821.1	Pra77024	Phytophthora ramorum	653	549	0.404464
PITG_09821.1	Pra44483	Phytophthora ramorum	226	122	0.406889
PITG_09821.1	Pra77022	Phytophthora ramorum	679	575	0.415716
PITG_09821.1	Pso132157	Phytophthora sojae	701	597	0.382034
PITG_09821.1	Pso132156	Phytophthora sojae	701	597	0.382034
PITG_09821.1	Pso140239	Phytophthora sojae	396	292	0.412158
PITG_09821.1	Pso140241	Phytophthora sojae	369	265	0.412158
PITG_10424.1	Pra82590	Phytophthora ramorum	283	82	0.161341
PITG_10424.1	Pra82586	Phytophthora ramorum	282	81	0.166951
PITG_10424.1	Pra46251	Phytophthora ramorum	226	25	0.325554
PITG_10424.1	Pra44445	Phytophthora ramorum	226	25	0.334018
PITG_10424.1	Pra82591	Phytophthora ramorum	1788	1587	0.379615
PITG_10424.1	Pso137800	Phytophthora sojae	187	14	0.253326
PITG_10424.1	Pso137799	Phytophthora sojae	297	96	0.302782
PITG_10424.1	Pso122879	Phytophthora sojae	200	1	0.358895
PITG_10424.1	Pso137796	Phytophthora sojae	278	77	0.370783
PITG_10424.1	Pso114189	Phytophthora sojae	193	8	0.387379
PITG_12828.1	Pra39825	Phytophthora ramorum	253	10	0.131584
PITG_12828.1	Pra74998	Phytophthora ramorum	263	0	0.134842
PITG_12828.1	Pra71773	Phytophthora ramorum	232	31	0.27619
PITG_12828.1	Pra80133	Phytophthora ramorum	262	1	0.286811
PITG_12828.1	Pra42333	Phytophthora ramorum	168	95	0.362146
PITG_12828.1	Pso109550	Phytophthora sojae	233	30	0.107652
PITG_12828.1	Pso143281	Phytophthora sojae	267	4	0.155276
PITG_12828.1	Pso140498	Phytophthora sojae	118	145	0.356683
PITG_12828.1	Pso122879	Phytophthora sojae	200	63	0.393192
PITG_12828.1	Pso114347	Phytophthora sojae	158	105	0.433051
PITG_12829.1	Pra39825	Phytophthora ramorum	253	10	0.131584
PITG_12829.1	Pra74998	Phytophthora ramorum	263	0	0.134842
PITG_12829.1	Pra71773	Phytophthora ramorum	232	31	0.27619
PITG_12829.1	Pra80133	Phytophthora ramorum	262	1	0.286811
PITG_12829.1	Pra42333	Phytophthora ramorum	168	95	0.362146
PITG_12829.1	Pso109550	Phytophthora sojae	233	30	0.107652
PITG_12829.1	Pso143281	Phytophthora sojae	267	4	0.155276
PITG_12829.1	Pso140498	Phytophthora sojae	118	145	0.356683
PITG_12829.1	Pso122879	Phytophthora sojae	200	63	0.393192

PITG_12829.1	Pso114347	Phytophthora sojae	158	105	0.433051
PITG_14167.1	Pra82586	Phytophthora ramorum	282	41	0.256595
PITG_14167.1	Pra82590	Phytophthora ramorum	283	40	0.258934
PITG_14167.1	Pra42333	Phytophthora ramorum	168	155	0.424255
PITG_14167.1	Pra44446	Phytophthora ramorum	181	142	0.484718
PITG_14167.1	Pra82591	Phytophthora ramorum	1788	1465	0.498517
PITG_14167.1	Pso137800	Phytophthora sojae	187	136	0.281317
PITG_14167.1	Pso137799	Phytophthora sojae	297	26	0.409129
PITG_14167.1	Pso114347	Phytophthora sojae	158	165	0.473056
PITG_14168.1	Pra82591	Phytophthora ramorum	1788	1483	0.241871
PITG_14168.1	Pra42333	Phytophthora ramorum	168	137	0.380374
PITG_14168.1	Pra44446	Phytophthora ramorum	181	124	0.387467
PITG_14168.1	Pra44485	Phytophthora ramorum	180	125	0.39602
PITG_14168.1	Pra82586	Phytophthora ramorum	282	23	0.400197
PITG_14168.1	Pso137796	Phytophthora sojae	278	27	0.233183
PITG_14168.1	Pso137800	Phytophthora sojae	187	118	0.401603
PITG_14168.1	Pso114347	Phytophthora sojae	158	147	0.428219
PITG_14168.1	Pso142500	Phytophthora sojae	195	110	0.477958
PITG_14168.1	Pso137799	Phytophthora sojae	297	8	0.481775
PITG_14287.1	Pra44512	Phytophthora ramorum	223	284	0.087251
PITG_14287.1	Pra77029	Phytophthora ramorum	243	264	0.100502
PITG_14287.1	Pra43398	Phytophthora ramorum	218	289	0.111147
PITG_14287.1	Pra87411	Phytophthora ramorum	557	50	0.133411
PITG_14287.1	Pra86169	Phytophthora ramorum	629	122	0.139405
PITG_14287.1	Pso145623	Phytophthora sojae	205	302	0.118694
PITG_14287.1	Pso122873	Phytophthora sojae	157	350	0.124041
PITG_14287.1	Pso144923	Phytophthora sojae	656	149	0.149916
PITG_14287.1	Pso132161	Phytophthora sojae	255	252	0.183481
PITG_14287.1	Pso114189	Phytophthora sojae	193	314	0.191295
PITG_14288.1	Pra44512	Phytophthora ramorum	223	255	0.097535
PITG_14288.1	Pra77029	Phytophthora ramorum	243	235	0.110136
PITG_14288.1	Pra43398	Phytophthora ramorum	218	260	0.120494
PITG_14288.1	Pra87411	Phytophthora ramorum	557	79	0.136072
PITG_14288.1	Pra86169	Phytophthora ramorum	629	151	0.142474
PITG_14288.1	Pso145623	Phytophthora sojae	205	273	0.128489
PITG_14288.1	Pso122873	Phytophthora sojae	157	321	0.136976
PITG_14288.1	Pso144923	Phytophthora sojae	656	178	0.15923
PITG_14288.1	Pso122879	Phytophthora sojae	200	278	0.161262
PITG_14288.1	Pso114189	Phytophthora sojae	193	285	0.191295

PITG_15296.1	Pra82590	Phytophthora ramorum	283	368	0.363832
PITG_15296.1	Pra82586	Phytophthora ramorum	282	369	0.366273
PITG_15296.1	Pra42333	Phytophthora ramorum	168	483	0.442163
PITG_15296.1	Pra44446	Phytophthora ramorum	181	470	0.486711
PITG_15296.1	Pso137800	Phytophthora sojae	187	464	0.272683
PITG_15299.1	Pra82590	Phytophthora ramorum	283	20	0.264475
PITG_15299.1	Pra82586	Phytophthora ramorum	282	19	0.297924
PITG_15299.1	Pso137800	Phytophthora sojae	187	76	0.426766
PITG_15299.1	Pso137799	Phytophthora sojae	297	34	0.443456
PITG_16160.1	Pra44512	Phytophthora ramorum	223	284	0.08756
PITG_16160.1	Pra77029	Phytophthora ramorum	243	264	0.100941
PITG_16160.1	Pra43398	Phytophthora ramorum	218	289	0.11484
PITG_16160.1	Pra87411	Phytophthora ramorum	557	50	0.133606
PITG_16160.1	Pra86169	Phytophthora ramorum	629	122	0.139607
PITG_16160.1	Pso145623	Phytophthora sojae	205	302	0.117585
PITG_16160.1	Pso122873	Phytophthora sojae	157	350	0.122524
PITG_16160.1	Pso144923	Phytophthora sojae	656	149	0.149626
PITG_16160.1	Pso132161	Phytophthora sojae	255	252	0.183268
PITG_16160.1	Pso114189	Phytophthora sojae	193	314	0.191295
PITG_17207.1	Pra44512	Phytophthora ramorum	223	264	0.08756
PITG_17207.1	Pra77029	Phytophthora ramorum	243	244	0.106505
PITG_17207.1	Pra43398	Phytophthora ramorum	218	269	0.11484
PITG_17207.1	Pra87411	Phytophthora ramorum	557	70	0.13188
PITG_17207.1	Pra86169	Phytophthora ramorum	629	142	0.138196
PITG_17207.1	Pso145623	Phytophthora sojae	205	282	0.117585
PITG_17207.1	Pso122873	Phytophthora sojae	157	330	0.122524
PITG_17207.1	Pso144923	Phytophthora sojae	656	169	0.14252
PITG_17207.1	Pso142501	Phytophthora sojae	573	86	0.190691
PITG_17207.1	Pso114189	Phytophthora sojae	193	294	0.192923
PITG_17208.1	Pra87411	Phytophthora ramorum	557	352	0.07338
PITG_17208.1	Pra44512	Phytophthora ramorum	223	18	0.076784
PITG_17208.1	Pra77029	Phytophthora ramorum	243	38	0.078918
PITG_17208.1	Pra86169	Phytophthora ramorum	629	424	0.083158
PITG_17208.1	Pra77024	Phytophthora ramorum	653	448	0.088195
PITG_17208.1	Pso144923	Phytophthora sojae	656	451	0.094428
PITG_17208.1	Pso132161	Phytophthora sojae	255	50	0.104322
PITG_17208.1	Pso145623	Phytophthora sojae	205	0	0.108018
PITG_17208.1	Pso142501	Phytophthora sojae	573	368	0.108018
PITG_17208.1	Pso122873	Phytophthora sojae	157	48	0.109934

PITG_17210.1	Pra44512	Phytophthora ramorum	223	99	0.095154
PITG_17210.1	Pra77029	Phytophthora ramorum	243	79	0.113551
PITG_17210.1	Pra43398	Phytophthora ramorum	218	104	0.12833
PITG_17210.1	Pra87411	Phytophthora ramorum	557	235	0.131245
PITG_17210.1	Pra86169	Phytophthora ramorum	629	307	0.133487
PITG_17210.1	Pso144923	Phytophthora sojae	656	334	0.172254
PITG_17210.1	Pso142501	Phytophthora sojae	573	251	0.194062
PITG_17210.1	Pso114189	Phytophthora sojae	193	129	0.212508
PITG_17210.1	Pso132157	Phytophthora sojae	701	379	0.23355
PITG_17210.1	Pso132156	Phytophthora sojae	701	379	0.23355
PITG_18477.1	Pra77024	Phytophthora ramorum	653	528	0.092207
PITG_18477.1	Pra86169	Phytophthora ramorum	629	504	0.092207
PITG_18477.1	Pra44512	Phytophthora ramorum	223	98	0.094009
PITG_18477.1	Pra87411	Phytophthora ramorum	557	432	0.094009
PITG_18477.1	Pra77029	Phytophthora ramorum	243	118	0.094009
PITG_18477.1	Pso142501	Phytophthora sojae	573	448	0.138171
PITG_18477.1	Pso145623	Phytophthora sojae	205	80	0.138171
PITG_18477.1	Pso132161	Phytophthora sojae	255	130	0.138355
PITG_18477.1	Pso144923	Phytophthora sojae	656	531	0.138355
PITG_18477.1	Pso132158	Phytophthora sojae	255	130	0.142919
PITG_18478.1	Pra87411	Phytophthora ramorum	557	115	0.18782
PITG_18478.1	Pra44446	Phytophthora ramorum	181	261	0.188083
PITG_18478.1	Pra77024	Phytophthora ramorum	653	211	0.188777
PITG_18478.1	Pra86169	Phytophthora ramorum	629	187	0.19453
PITG_18478.1	Pra42333	Phytophthora ramorum	168	274	0.204075
PITG_18478.1	Pso122873	Phytophthora sojae	157	285	0.150589
PITG_18478.1	Pso122879	Phytophthora sojae	200	242	0.166817
PITG_18478.1	Pso145623	Phytophthora sojae	205	237	0.18078
PITG_18478.1	Pso114189	Phytophthora sojae	193	249	0.191295
PITG_18478.1	Pso144923	Phytophthora sojae	656	214	0.215299
PITG_18482.1	Pra44512	Phytophthora ramorum	223	272	0.087646
PITG_18482.1	Pra77029	Phytophthora ramorum	243	252	0.101237
PITG_18482.1	Pra43398	Phytophthora ramorum	218	277	0.114959
PITG_18482.1	Pra87411	Phytophthora ramorum	557	62	0.135386
PITG_18482.1	Pra86169	Phytophthora ramorum	629	134	0.141578
PITG_18482.1	Pso145623	Phytophthora sojae	205	290	0.11771
PITG_18482.1	Pso122873	Phytophthora sojae	157	338	0.122695
PITG_18482.1	Pso144923	Phytophthora sojae	656	161	0.144077
PITG_18482.1	Pso132161	Phytophthora sojae	255	240	0.182643

PITG_18482.1	Pso114189	Phytophthora sojae	193	302	0.191295
PITG_19366.1	Pra44512	Phytophthora ramorum	223	272	0.092437
PITG_19366.1	Pra77029	Phytophthora ramorum	243	252	0.105643
PITG_19366.1	Pra43398	Phytophthora ramorum	218	277	0.119999
PITG_19366.1	Pra87411	Phytophthora ramorum	557	62	0.137632
PITG_19366.1	Pra86169	Phytophthora ramorum	629	134	0.143832
PITG_19366.1	Pso122873	Phytophthora sojae	157	338	0.109321
PITG_19366.1	Pso145623	Phytophthora sojae	205	290	0.112799
PITG_19366.1	Pso144923	Phytophthora sojae	656	161	0.147199
PITG_19366.1	Pso132161	Phytophthora sojae	255	240	0.187221
PITG_19366.1	Pso114189	Phytophthora sojae	193	302	0.192199
PITG_19909.1	Pra75227	Phytophthora ramorum	441	316	0.435554
PITG_20992.1	Pra75227	Phytophthora ramorum	441	319	0.432161
PITG_21462.1	Pra75227	Phytophthora ramorum	441	39	0.26459
PITG_21462.1	Pra75226	Phytophthora ramorum	451	49	0.35397
PITG_21462.1	Pra75225	Phytophthora ramorum	454	52	0.398566
PITG_21462.1	Pra39775	Phytophthora ramorum	344	58	0.398954
PITG_21462.1	Pra46219	Phytophthora ramorum	353	49	0.411837
PITG_21462.1	Pso145395	Phytophthora sojae	442	40	0.242538
PITG_21462.1	Pso144173	Phytophthora sojae	427	25	0.247161
PITG_21462.1	Pso144196	Phytophthora sojae	457	55	0.365081
PITG_21462.1	Pso144197	Phytophthora sojae	457	55	0.369281
PITG_21462.1	Pso144172	Phytophthora sojae	222	180	0.395161
PITG_21463.1	Pra75225	Phytophthora ramorum	454	16	0.324361
PITG_21463.1	Pra75227	Phytophthora ramorum	441	29	0.376793
PITG_21463.1	Pra39775	Phytophthora ramorum	344	126	0.381838
PITG_21463.1	Pra46219	Phytophthora ramorum	353	117	0.404817
PITG_21463.1	Pra75226	Phytophthora ramorum	451	19	0.42687
PITG_21463.1	Pso144172	Phytophthora sojae	222	248	0.25269
PITG_21463.1	Pso144191	Phytophthora sojae	489	19	0.36293
PITG_21463.1	Pso145119	Phytophthora sojae	364	106	0.39309
PITG_21463.1	Pso124664	Phytophthora sojae	192	278	0.413662
PITG_21463.1	Pso145395	Phytophthora sojae	442	28	0.44488
PITG_21499.1	Pra85622	Phytophthora ramorum	420	179	0.289294
PITG_21499.1	Pra85623	Phytophthora ramorum	939	698	0.354172
PITG_21499.1	Pra86921	Phytophthora ramorum	513	272	0.478736
PITG_21499.1	Pso141647	Phytophthora sojae	402	161	0.283517
PITG_21499.1	Pso140268	Phytophthora sojae	444	203	0.341272
PITG_21499.1	Pso140272	Phytophthora sojae	974	733	0.343543

PITG_21499.1	Pso132225	Phytophthora sojae	974	733	0.343543
PITG_21499.1	Pso145391	Phytophthora sojae	824	583	0.343604
PITG_21714.1	Pra80133	Phytophthora ramorum	262	86	0.044989
PITG_21714.1	Pra71773	Phytophthora ramorum	232	56	0.050711
PITG_21714.1	Pra39825	Phytophthora ramorum	253	77	0.186696
PITG_21714.1	Pra74998	Phytophthora ramorum	263	87	0.192068
PITG_21714.1	Pra44446	Phytophthora ramorum	181	5	0.320036
PITG_21714.1	Pso140498	Phytophthora sojae	118	58	0.214536
PITG_21714.1	Pso143281	Phytophthora sojae	267	91	0.224151
PITG_21714.1	Pso109550	Phytophthora sojae	233	57	0.231281
PITG_21714.1	Pso140239	Phytophthora sojae	396	220	0.334632
PITG_21714.1	Pso140241	Phytophthora sojae	369	193	0.334632
PITG_22166.1	Pra44483	Phytophthora ramorum	226	90	0.168941
PITG_22166.1	Pra44487	Phytophthora ramorum	219	83	0.168941
PITG_22166.1	Pra44446	Phytophthora ramorum	181	45	0.174156
PITG_22166.1	Pra42333	Phytophthora ramorum	168	32	0.183388
PITG_22166.1	Pra86169	Phytophthora ramorum	629	493	0.198425
PITG_22166.1	Pso132156	Phytophthora sojae	701	565	0.139292
PITG_22166.1	Pso132157	Phytophthora sojae	701	565	0.139292
PITG_22166.1	Pso114189	Phytophthora sojae	193	57	0.163968
PITG_22166.1	Pso140239	Phytophthora sojae	396	260	0.189474
PITG_22166.1	Pso140241	Phytophthora sojae	369	233	0.189474
PITG_22227.1	Pra71773	Phytophthora ramorum	232	1	0.252838
PITG_22227.1	Pra80133	Phytophthora ramorum	262	29	0.255162
PITG_22227.1	Pra44446	Phytophthora ramorum	181	52	0.359794
PITG_22227.1	Pra42333	Phytophthora ramorum	168	65	0.37533
PITG_22227.1	Pra39825	Phytophthora ramorum	253	20	0.404586
PITG_22227.1	Pso142500	Phytophthora sojae	195	38	0.393146
PITG_22227.1	Pso140498	Phytophthora sojae	118	115	0.412556
PITG_22227.1	Pso143281	Phytophthora sojae	267	34	0.479307
PITG_22227.1	Pso114347	Phytophthora sojae	158	75	0.479811
PITG_22227.1	Pso144923	Phytophthora sojae	656	423	0.479948
PITG_22632.1	Pra71773	Phytophthora ramorum	232	132	0.480138
PITG_22632.1	Pra80133	Phytophthora ramorum	262	162	0.480138
